# Bubble-raft inspired shape-assembly in flying robot swarm for uniform formation and obstacle traversal

**DOI:** 10.1038/s44172-026-00715-3

**Published:** 2026-07-02

**Authors:** Hanxun Li, Jinshu Su, Zhiqiang Li, Yining Zhao, Tingyu Chen, Biao Han

**Affiliations:** College of Computer, National University of Defense Technology, Changsha, China

**Keywords:** Engineering, Mathematics and computing

## Abstract

Natural swarms can move cohesively while continuously reshaping their spatial shape, yet reproducing this behavior with large flying robot swarms in obstacle environments remains difficult, especially in achieving uniformity and safety. Here, we present a distributed 3D shape-assembly controller for flying robot swarms that forms arbitrary target shapes with uniform coverage and safe inter-robot distance. Inspired by bubble-rafts, each robot is associated with a non-overlapping region inside the target shape and updates its velocity by considering region exploration, region balancing and distance fine-tuning. The proposed region-based shape-assembly method is able to drive the swarm toward uniform formations and enable fast shape transformation, as well as resilience to robot joining and failure. For navigation, we design a lightweight velocity-obstacle mechanism that adjusts the nominal shape-assembly velocity to collision-free commands with minimal formation disruption. We validate the proposed methods in extensive simulations and real-world swarm experiments, demonstrating efficient formation of complex 3D shapes, robust maintenance of safety distances, and reliable obstacle traversal.

## Abstract (web summary)

Biao Han and colleagues report a bubble-raft inspired method for flying robot swarms to form 3D shapes with uniform coverage. The proposed method is able to drive the swarm toward uniform formation of complex 3D shapes and enable fast shape transformation, robust maintenance of safety distances, and reliable obstacle traversal.

## Introduction

An autonomous flying robot swarm that can organize itself, navigate through cluttered environments and accomplish complex missions as a single coherent entity has always been a future vision. In nature many animal groups already display this level of collective competence, as bird flocks migrate in cohesive formations^1^, fish schools weave through reefs while splitting and merging^2^, and fire ants assemble floating rafts or bridges when their habitat is flooded^3,4^. A large-scale flying robot swarm that could approach this behavior would greatly extend what robots can do in search and rescue, environmental monitoring^5^, communication relaying and cooperative transportation. Realizing this potential requires attention not only to where each robot goes but also to how the group distributes itself in space while it operates. In disaster response^6,7^, uniform coverage of the affected area determines how quickly victims can be detected. In communication tasks^8^, the geometry of the swarm shapes the quality and robustness of the relay network. In cooperative transportation^9,10^, the relative positions of drones determine whether loads are carried safely. To cope with these demands in realistic obstacle-rich environments, the swarm mainly needs the following complementary capabilities: 1) form task-specific spatial formations at scale according to the mission, 2) move through obstacles while preserving these formations and avoiding collisions among robots and with the environment.

However, achieving these capabilities in large-scale swarms remains challenging. In most existing formation-control frameworks the formation is described as a set of target points, and each robot in the swarm is assigned to a unique goal position^11,12^. For flying robots this mapping-based strategy is usually implemented in two stages, namely target assignment and collision-free path planning^13,14^. A global planner first matches robots to targets using methods including simultaneous assignment^15,16^, graph-similarity^14,17–19^, and optimization^20^, and then computes coordinated trajectories that drive robots from their current positions to the assigned goals without collisions using methods including MIQP^21^, MPC^22^. With access to the full swarm state such methods can generate accurate formations, but both the assignment problem and the joint trajectory planning become much more complex as the number of robots increases. To achieve formation flight at large scale, many works draw inspiration from bird flocks and design flocking controllers based on local repulsion, alignment and attraction, while hundreds of robots can move as a cohesive group without centralized planning^23–25^. However, since collision avoidance relies mainly on repulsive forces, their performance degrades in densely cluttered environments^26^, and the group typically remains a loose crowd rather than forming precise task-dependent formations.

For large-scale swarm formation, the limitations of flocking have motivated a different assignment-free approach known as shape assembly which has attracted considerable attention^27–29^. This approach does not require assigning specific target positions to individual robots. It only needs the outline or topology of the desired shape, and complex formations emerge through the states and positions of neighboring robots^30–33^. Current research on shape assembly mainly focuses on ground robots, which can self-organize into two-dimensional snowflakes, wrench shapes, stars, letters, and more^34,35^. Cheng et al. ^36^ proposed a gas model inspired algorithm, which used a dispersion model to form arbitrary shapes and could repair broken structures after agent failures, although the generated patterns were limited in precision and uniformity. Sun et al. ^37^ further proposed a Mean-shift exploration strategy in which robots interpret the desired shape as a density field, repeatedly give up their current locations and move toward nearby unoccupied regions of higher shape density, enabling large ground swarms to assemble highly non-convex shapes. However, during shape transitions the robots still rely mainly on repulsive interactions with neighbors for collision avoidance, the safety of the swarm cannot be rigorously guaranteed. To achieve dynamic pattern formation in obstacle environments, Li et al. ^38^ combined a GRF-based predictive flocking controller with a Mean-shift style region shape control, allowing the robot swarm to track patterns while passing obstacles. However, the method was only validated in corridor layouts and the distribution in target shape remains nonuniform. Overall, existing shape-assembly methods still lack explicit mechanisms to ensure uniformly filled formations and to enable the swarm to move safely through dense environments using controllers of sufficiently low complexity.

To address the first challenge of forming task-specific formations at scale, this paper presents a uniform three-dimensional shape-assembly method for flying robot swarms, enabling rapid formation of arbitrary 3D shapes and ensuring safe flight in environments with obstacles. Inspired by the uniformly distributed bubble rafts on water^39^, we propose a balanced exploration shape-assembly algorithm grounded in the bubble raft’s physical principles underlying the formation mechanism. The main idea is to assign each robot a non-overlapping region based on distance, within which it exclusively explores. The robot then updates its velocity by combining three terms: a region-exploration term that pulls it toward the center of its region, a region-balancing term that equalizes the sizes of neighboring regions, and a distance fine-tuning term that regulates the average distance to neighboring robots. By jointly adjusting region size and inter-robot spacing in this way, the swarm is driven to fill the target shape with an approximately uniform distribution, while also supporting fast transformation between shapes and natural recovery when robots join or fail. To address the second challenge of moving through an obstacle environment while preserving formation, we further integrate the concept of velocity obstacles^40^ by treating the exploration velocity as a nominal command and slightly adjusting it to the best collision-free velocity with respect to shape preserving and smoothness. We demonstrate the effectiveness of these methods in both simulation and real-world swarm flight experiments.

## Results

### Platforms and experimental setup

In all simulations and experiments, each robot can estimate its own three-dimensional position, velocity and acceleration, and can exchange position and velocity information with neighboring robots within its sensing range via short-range wireless communication. The control law is implemented in a fully distributed manner, and the control input of each robot is computed from its own state, the states of its neighbors and the target shape without a centralized planner. We evaluate the proposed method both in high-fidelity simulation and on real hardware. In simulation we use Unreal Engine^41^ with the AirSim framework^42^, where each virtual robot is equipped with a 3D LiDAR sensor to perceive nearby obstacles. In real-world experiments we deploy up to 18 Crazyflie 2.1 micro quadrotors^43^ in an indoor arena, and use a Nokov motion-capture system to provide the position and velocity of each drone, which are then fed to the same distributed shape-assembly and obstacle-avoidance controller as in simulation.

### Adaptivity to complex 3D non-convex shape

**Fig. 1:**
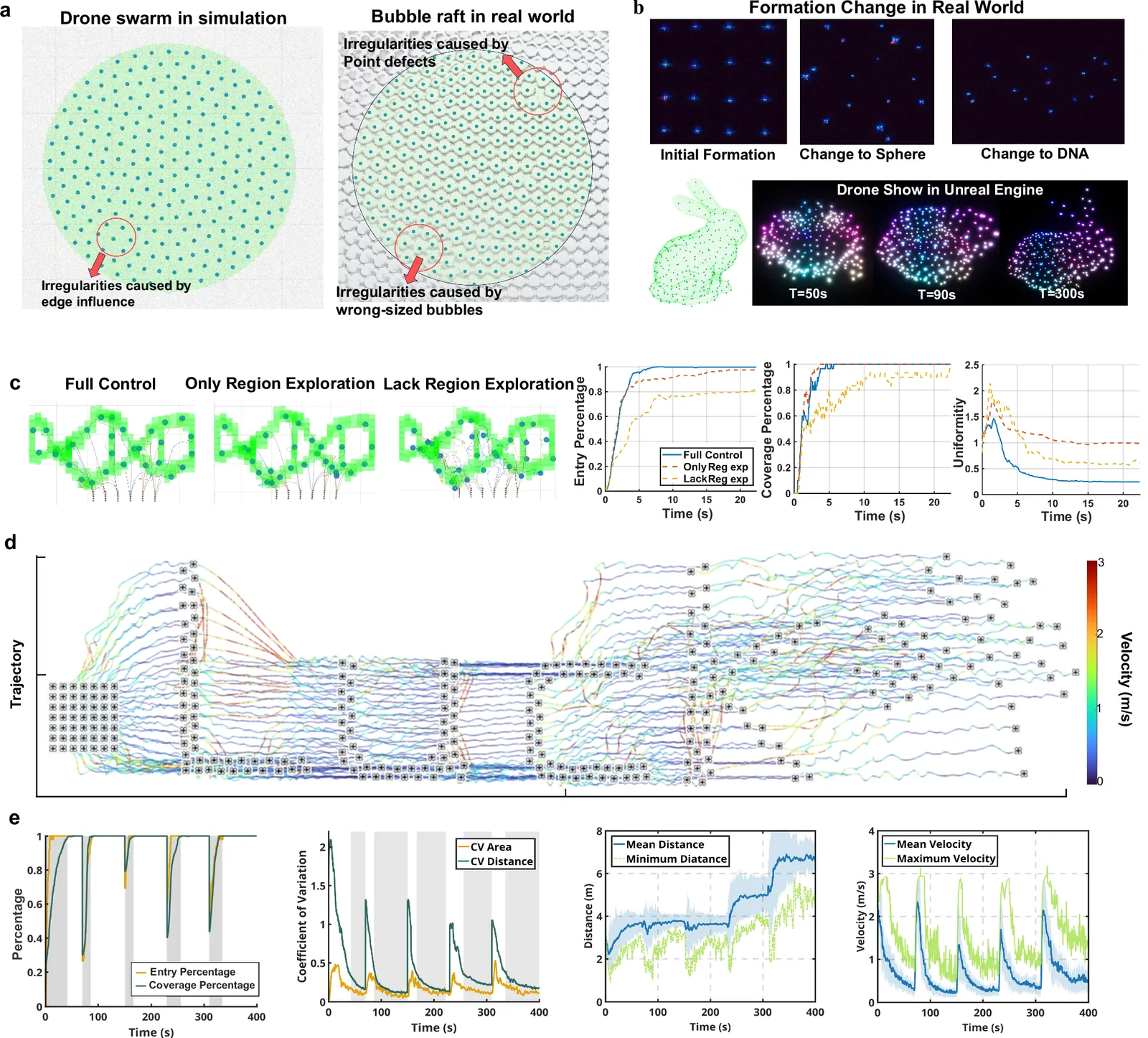
Complex 3D shape assembly. **a** Comparison between bubble rafts in nature and the resulting formation produced by our algorithm **b** In real-world experiments, a swarm of 16 drones changes formation from a square to a sphere and then to a DNA-like double helix (see Supplementary Video 1) and in simulation a swarm of 300 drones forms a 3D bunny. **c** In an ablation experiment, the robot assembles 3D DNA structures under three conditions: full control, without region-exploration, and with region-exploration only. **d** In simulation, 49 robots sequentially form the letters “L”, “U”, “C”, “K” and then assemble into a clover shape while flying at a fixed speed. Another experiment shows the swarm switching between the letters “S”, “W”, “A”, “R”, and “M” (see Supplementary Video 2). **e** Time evolution of key formation metrics during repeated resizing: from left to right, entry percentage and coverage percentage; coefficient of variation of region area and neighbor’s distance; mean and minimum neighbor’s distance; and mean and maximum robot velocity.

Unlike traditional formation control approaches that assign precise coordinates to each robot, we adopt a shape-based formation control strategy, where thousands of target points define a fine-grained geometric shape that serves as the desired swarm configuration. To bridge the gap between this discrete set of target points and the size of physical robots, we take inspiration from bubble rafts. As shown in Fig. 1a, bubbles floating on water adjust their mutual distances and areas until they form a nearly uniform raft. In our method the target points play the role of the water surface, the robots act as bubble centers, and each bubble is abstracted as the region of target points owned by a robot. Following this analogy we introduce a region-balancing term and a distance fine-tuning term that regulate the size of these regions and the spacing between neighboring robots. Simulations show that the swarm arranges itself into an almost uniform packing similar to a bubble raft, and we observe comparable small irregularities caused by point defects near the boundary. More information can be found in Supplementary Information Sec. 1.

Building on this bubble-raft-like behavior, our self-assembly strategy is also capable of rapidly generating complex, non-convex 3D structures. As a canonical example, the DNA double helix consists of two antiparallel strands that entwine and bond to form a twisted three-dimensional shape. We use 16 Crazyflie drones to realize formation transitions from an initial configuration to a sphere and then to a DNA-like double helix (Fig. 1b), achieving collision-free, nearly complete coverage of each target shape with high spatial uniformity (see Supplementary Video 1). To further assess scalability and geometric complexity, we also performed a large-scale simulation with 300 virtual drones assembling into a three-dimensional bunny-shaped formation. More results on the generation of complex shapes are provided in Supplementary Information Section 3.

We further evaluate the contribution of each component of the exploration command using ablation experiments (Fig. 1c). When the region-exploration term is removed, robots lose a clear directional cue from their associate regions and only react to neighbor positions, which leads to oscillatory motion near the boundary and leaves large parts of the shape unfilled. The remaining two terms merely pull robots closer to or push them farther from neighbors and cannot fully explore the interior volume. Removing either the region-balancing term or the distance-fine-tuning term similarly undermines exploration: robots that enter the target tend to accumulate near corners and narrow parts of the shape instead of spreading out, and the final formation exhibits noticeably nonuniform coverage.

Beyond static shapes, our controller also adapts when the target shape changes in size. In an Unreal Engine simulation with AirSim, we demonstrate 49 drones smoothly reconfiguring between differently sized target shapes (Fig. 1d). As shown in Fig. 1e, the exploration and region-balancing terms drive the swarm to quickly spread inside each new shape, the entry percentage and coverage percentage both rise to nearly 1.0 within about 40 s after each change, while the coefficients of variation of region area and neighbor distance drop rapidly. The minimum inter-robot distance stays well above the 0.4 m safety threshold throughout all transitions, showing that the swarm can adapt to differently sized formations without sacrificing safety.

### Shape recovery in dynamic swarm size

**Fig. 2:**
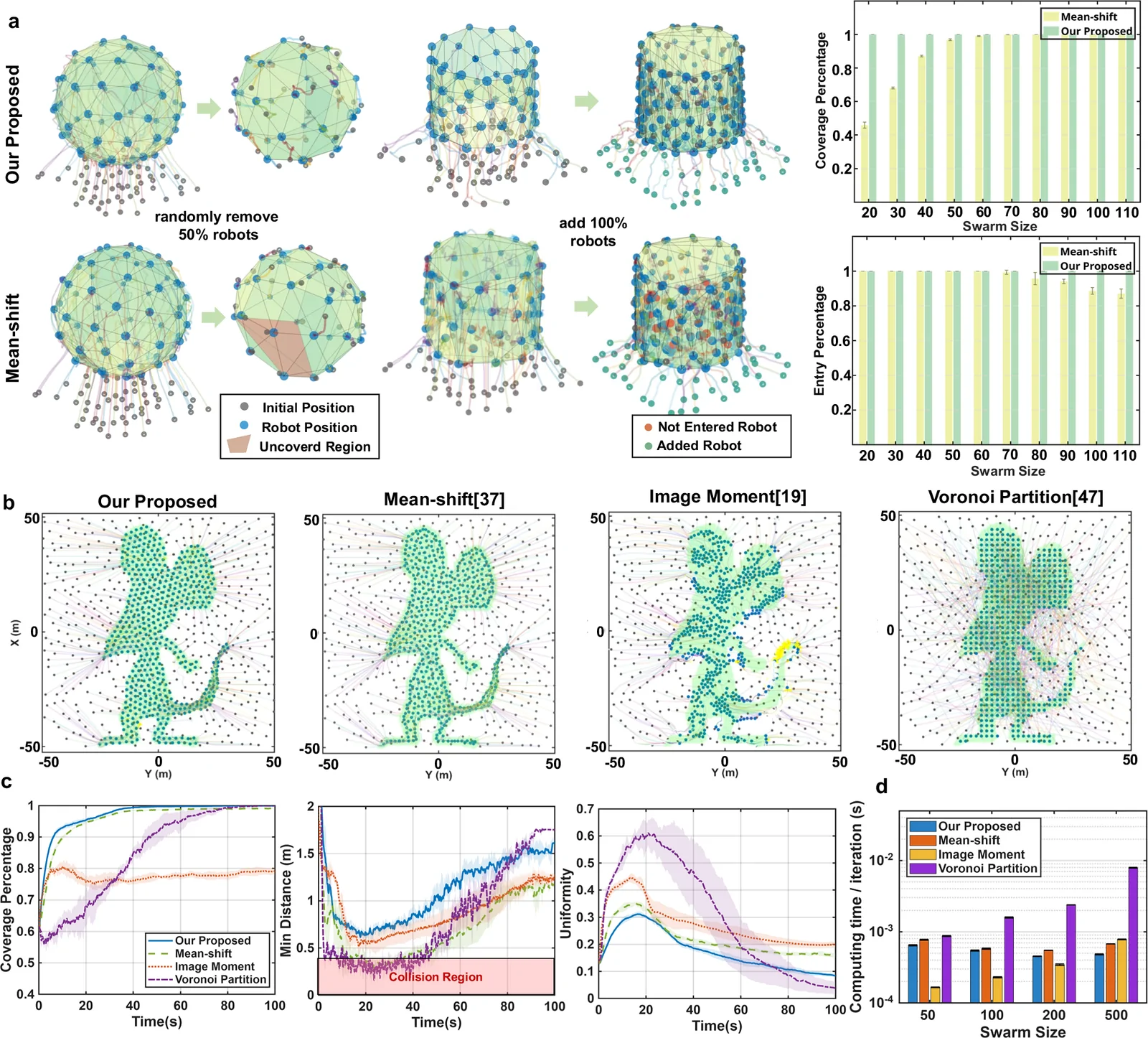
Comparison of formation recovery and shape generation under different methods. **a** Comparison for self-healing ability through robot removal and addition with Mean-shift. **b** Formation for a cartoon mouse shape using 500 robots under our method, Mean-shift, Image Moment and Voronoi Partition. (More comparison can be found in Supplementary Video 4) **c** Changes in coverage percentage, minimum inter-robot distance, and uniformity during the shape assembly process. Shaded regions denote the 90% confidence interval over 10 trials. **d** Computing time per iteration across different swarm sizes.

Shape recovery is a critical capability for aerial robot swarms^36^. Our method preserves the target formation even when robots fail or new robots join. In Fig. 2a, we first arranged 60 robots into a spherical formation, then randomly removed 50 % of them. Under the exploration command, our proposed method successfully repairs the shape and fills in the missing regions. By contrast, the Mean-shift method^37^ targets low-density areas within each robot’s sensing range to cover the shape. However, after we removed half of the robots, the swarm was too sparse, which caused target-point densities to even out, exploration velocities to drop, resulting in large regions remaining uncovered in Mean-shift method. Next, we formed a cylindrical formation with 60 robots and then added another 60 robots. Our method responded by adaptively reducing inter-robots distance so that all robots stayed within the target shape. In Mean-shift, mutual repulsion caused many robots to spill outside the cylinder, reducing the entry rate. Whether we halve or double the swarm size, our algorithm achieves 100 % entry and coverage rates. However in Mean-shift method, as shown in Fig. 2a, coverage falls when the swarm size shrinks by one-third, and entry rate drops when it grows by one-third. We also validated our approach in real-world flights with 18 Crazyflie drones. After forming a spherical formation, we removed them one by one; the remaining drones rapidly reformed the sphere (see Supplementary Video 3).

### Performance comparison with existing formation algorithms

To validate our method’s advantages in coverage of target shape, safety, uniformity and lightweight implementation, we compared it against three formation methods: Mean-shift^37^, Image Moment^19^, and Voronoi Partition^44^. For the Image Moment method, the target formation is given directly as the binary mouse image. For Voronoi Partition, we uniformly sample 500 points inside the black region of this image as target points. For Mean-shift, we use the authors’ open-source implementation to quantize the binary image into a grid of occupied cells as the target formation, and we take the center of each occupied cell as a target point for our method. The sensing range is set to 5 m in four methods.

We evaluated all four methods by forming a cartoon mouse shape in 10 independent runs using 500 robots. In these experiments we measure swarm uniformity using only the coefficient of variation of inter-robot distances since the Voronoi-based baseline doesn’t provide an associate-region definition that enables consistent computation of region-size variation, and we report coverage percentage, minimum distance in robot swarm and uniformity index over time in Fig. 2c. As Fig. 2b shows, our method produces an almost uniform density across the entire shape. Robots that initially cluster near the tail are gradually steered by the region-balancing exploration command to slide along the tail and spread into the body, which is reflected in the rapid increase of coverage. Throughout this process the minimum inter-robot distance curve stays above the collision region, indicating that the robots remain well separated while the shape fills. Mean-shift can also reach a high coverage level, but it struggles along the narrow arms, legs and tail. Each robot only reacts to its local density estimate and stops exploring once the nearby density appears balanced, leaving uncovered regions in the middle part of the shape and the uniformity remains high compared with our method. Local conflicts between target cells further drive neighboring robots toward the same area, leading to collisions between robots.

The Image Moment method uses Legendre and pseudo-Zernike image moments to drive the robots toward the desired shape, however it mainly assembles the outer outline of the mouse and fails to capture its interior details. As a result, its coverage rate in Fig. 2c increases slowly and ends at a relatively low level compared with our method. Voronoi Partition successfully forms the desired shape, however a few robots in the central region still fail to reach their target points and the formation converges more slowly than with other methods. This is because the method partitions the space into Voronoi cells based on robots’ position and relies on the exchanging targets between neighboring regions to resolve conflicts. These frequent exchanges cause large amounts of ineffective motion, and some robots are assigned to opposing target points, which induces conflicting motion directions and repeated collision-avoidance maneuvers, resulting in slow convergence and occasional failure to reach the assigned targets. More detailed comparisons and analysis with Mean-shift are provided in Supplementary Information Section 4.

We also measured the computing time per iteration across varying swarm sizes, as shown in Fig. 2d. Our method, along with Mean-shift and Image Moment, shows only minor changes in computation time, while Voronoi Partition becomes increasingly inefficient as the swarm size grows, due to repeated global Voronoi partitioning and destination switching among neighboring robots that introduce substantial additional computation.

### Collision avoidance and formation keeping in unknown environments

To evaluate the formation-based obstacle avoidance capabilities of our swarm formation control method, we constructed a dense forest environment in Unreal Engine and 25 robots arranged in a square formation to navigate through this cluttered space at a speed of 2 m/s. Each robot was equipped with LiDAR sensors to perceive obstacles.

**Fig. 3:**
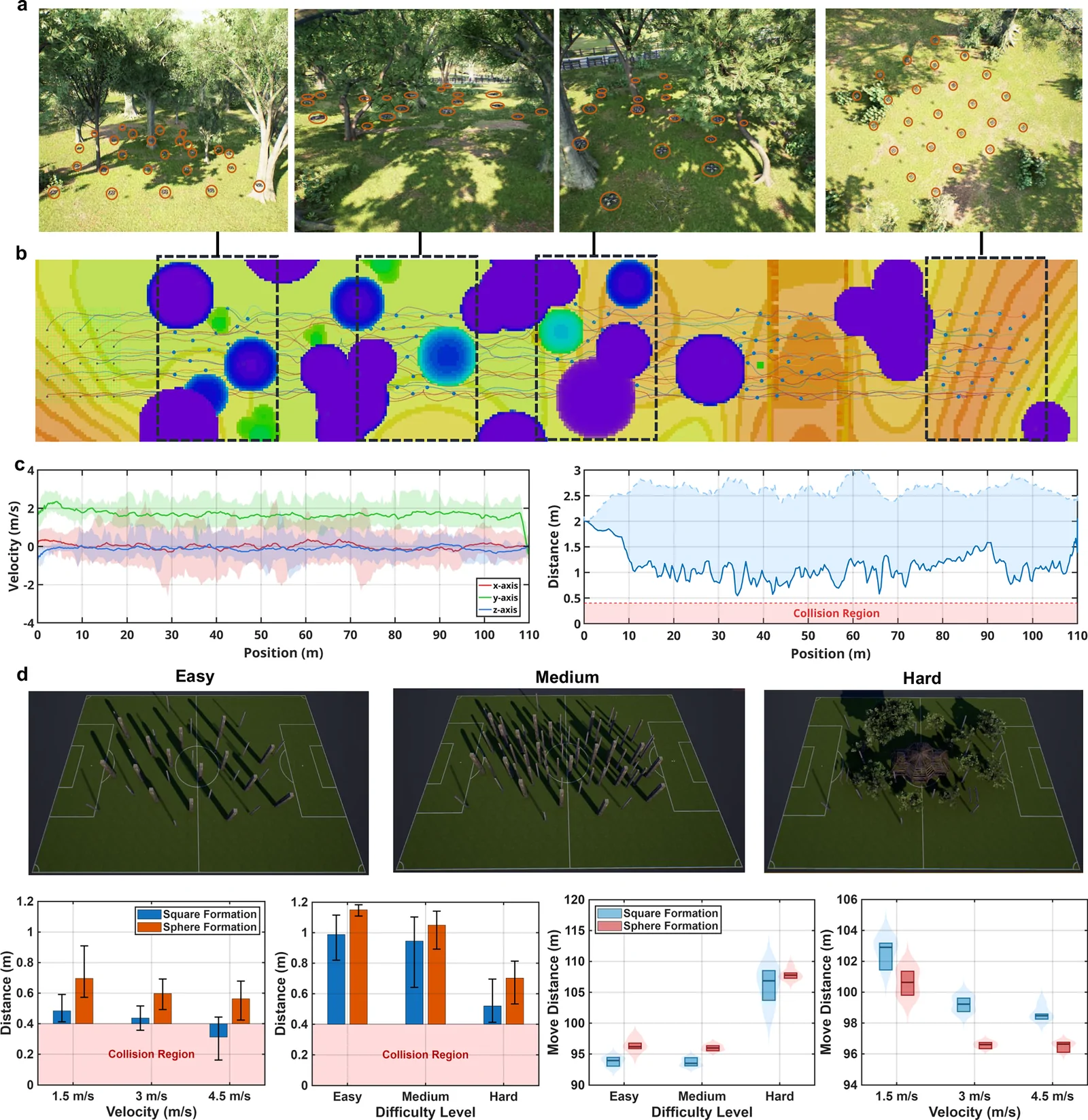
Swarm formation control in dense environment. **a** Snapshots of a swarm flying through obstacles in the Unreal Engine simulation, corresponding to marked positions in b. **b** The swarm maintains a square formation while traversing an 85 m-long obstacle-filled environment. (See Supplementary Video 5) **c** Velocity profiles along all three axes during flight, shaded regions represent the maximum and minimum values. **d** The minimum distance and move distance at different densities and speeds. For the two plots on the left, the error bars indicate the minimum and maximum values over 10 experimental runs. For the two plots on the right, the inner box and horizontal line indicate the interquartile range and the median, respectively.

As shown in Fig. 3b, the swarm deviates laterally to avoid obstacles and then gradually regroups to restore the original formation. A key feature of our method is that the velocity obstacles are decomposed into contributions from the environment and from neighboring robots. Treating nearby robots as obstacles prevents the group from collapsing onto one side of an obstacle, and subtracting obstacle-occupied portions from each robot’s region avoids assigning unreachable or unsafe target points. Figure 3d confirms that the minimum distances between robots and between robots and obstacles remain above the 0.4 m safety threshold throughout the flight. The swarm can also traverse narrow corridors under the same controller, as detailed in Supplementary Information Section 5.2.

We further evaluated our method in three environments of increasing difficulty, where the average distance between obstacles was 15 m, 8 m, and 4 m, respectively (Fig. 3d). In each setting, both square and

Spherical formations were flown 10 times through the obstacle zone at different speeds. As shown in Fig. 3d, the spherical formation is more robust in terms of safety, even at 4.5 m/s the swarm in a spherical formation keeps the minimum distance to obstacles above the 0.4 m safety threshold, while the square formation starts to experience collisions under the same condition. In terms of moving distance, the spherical formation has a shorter displacement distance at high speeds. These results are directly related to the geometry of the two formations. In square formation all target points lie on a single plane, when the swarm encounters obstacles robots can only adjust their positions within that plane and many of them are forced to compete for the same narrow lateral gaps. In contrast, the spherical formation distributes target points throughout a three-dimensional volume, which allows each robot to adjust its altitude as well as its horizontal position while still staying close to its associate region. Parts of the formation can pass over or under obstacles instead of squeezing through one layer, so the shape is better preserved and the minimum distances between robots and obstacles remain larger, leading to safer and smoother trajectories.

### Comparison with existing methods in obstacle environments

**Fig. 4:**
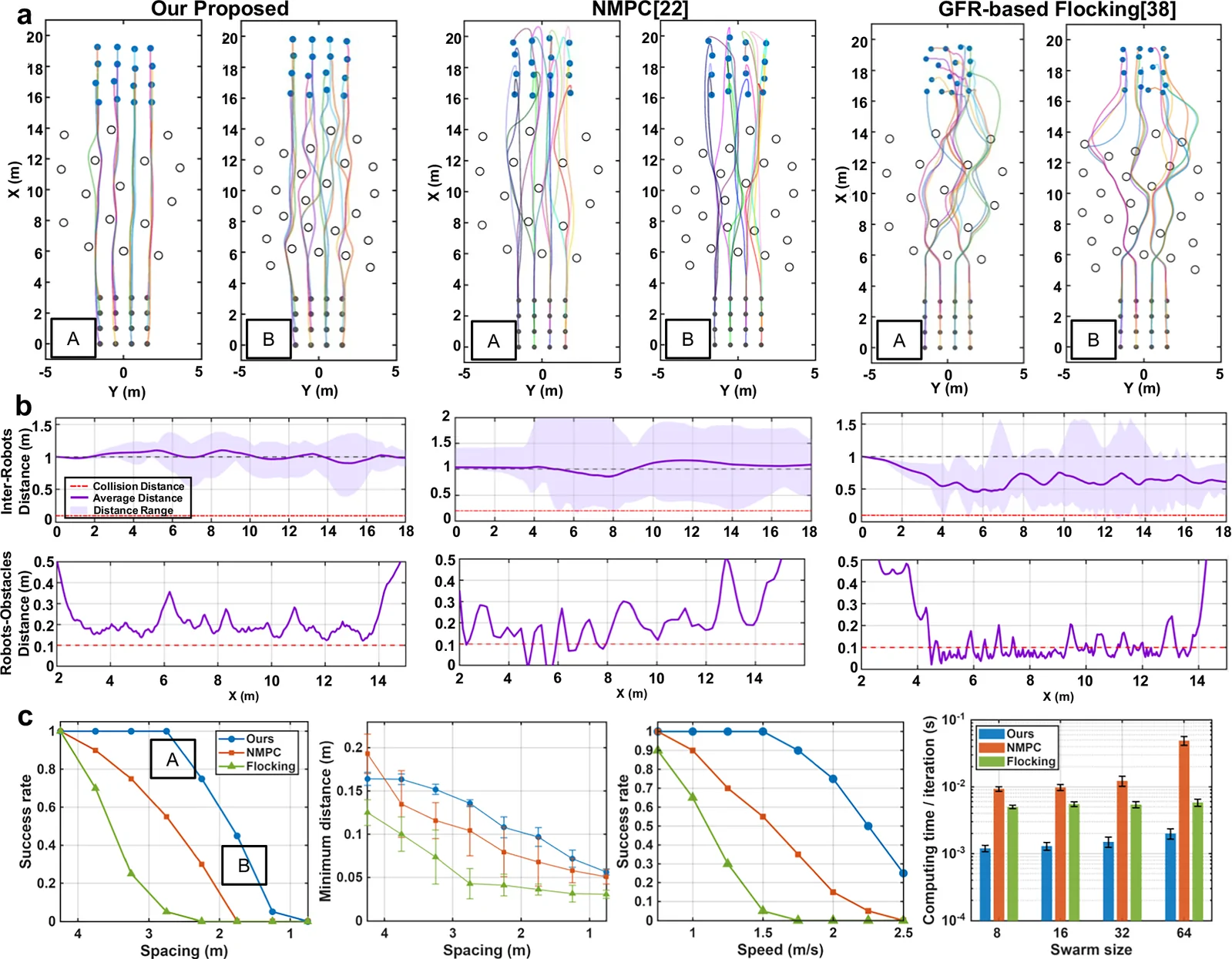
Comparison between different obstacles traversal methods. **a** Trajectories of the three methods in different density obstacles, the spacing between the obstacles for A and B are 2.75 and 1.75 respectively. **b** Inter-robots distance and robots-obstacles distance under case B in 3 methods, the shaded region denotes the range between the maximum and minimum distances during flight. **c** Success rate and minimum distance at 1.5 m/s under various spacing. Success rate in case A under various speed, error bar represents the upper and lower limits over 10 runs. Computing time per iteration in various swarm sizes.

In obstacle environments we compared our controller with NMPC^22^ and GRF-based flocking^38^. Each swarm flew in a forest-like field with many cylindrical obstacles, and the desired speed of all robots was set to 1.5 m/s. For NMPC we use 16 fixed target points and let the controller plan motions for each robot to reach its own target. For our method and for flocking we instead specified a desired formation shape together with its center, and robots tried to keep this shape while moving through the obstacles. As shown in Fig. 4a, we show the trajectories produced by the three methods when the average obstacle spacing is 2.75 m and 1.75 m, with our method the swarm bends smoothly around the obstacles and the formation stays roughly intact, while NMPC and GRF-based flocking produce more twisted paths and the group shape is harder to recognize.

As shown in Fig. 4b, we further examine in environment B how the distances between robots and between robots and obstacles change along the flight path. In the desired formation the nominal spacing between neighbors is 1 m, and under our controller the inter-robot distance stays close to this value with only small fluctuations, while the distance to obstacles also remains well above the 0.1 m safety limit. This comes from the region-based design in which robots are evenly distributed over the target shape, each robot owns a region of similar size and performs obstacle avoidance mainly inside its own region. Robots therefore do not need to make large mutual detours around obstacles, they interfere with each other much less during avoidance. In contrast, under NMPC each robot is assigned a fixed target and all trajectories are optimized together. When obstacles are dense the optimizer has to bend many paths to let every robot reach its own target, which squeezes some robots toward obstacles and others toward their neighbors and finally leads to collisions. The GRF-based flocking method moves the robots through gradients generated by neighbors, obstacles and a soft shape potential. In dense obstacles this gradient pulls robots toward the same gaps around obstacles, the formation is easily distorted and robots crowd together, leading to small inter-robot distances and more collisions.

In Fig. 4c we summarize results over random obstacle fields. For each obstacle spacing and each flight speed we generate ten different layouts and run all three methods. As spacing decreases, our controller keeps a success rate close to 100%, while NMPC starts to fail at larger gaps and flocking drops to almost zero in dense cases. The minimum-distance plot shows the same trend: our method maintains the largest clearance to obstacles, NMPC is in the middle and flocking has the smallest margins. When we increase the speed, our method still keeps most runs collision-free, whereas the success rates of NMPC and flocking fall quickly. The computing-time bars on the right further show that our per-iteration cost grows slowly with swarm size and remains much lower than that of NMPC.

### Flying through obstacles in real world

**Fig. 5:**
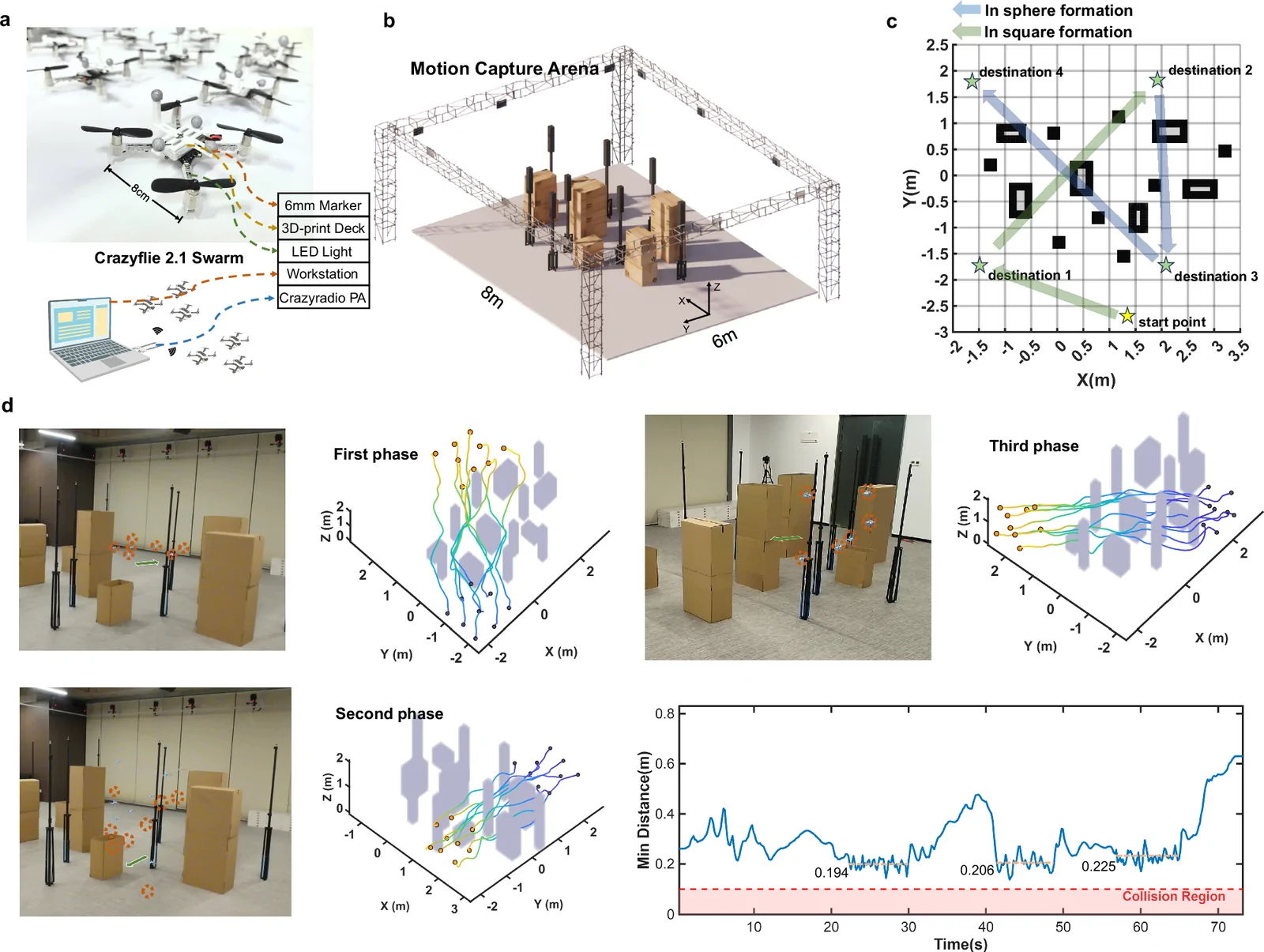
Swarm obstacle avoidance in a real-world environment. **a** The structure of Crazyflie drones. **b** The motion capture arena, measuring 6 by 8 meters, is filled with obstacles made from cardboard boxes and camera poles. **c** Top-down view of the arena showing three flight phases with different formation strategies. (See Supplementary Video 6) **d** First phase: the swarm maintains a square formation while navigating through obstacles. Second phase: the swarm starts in a square formation and transitions to a spherical formation. Third phase: the swarm traverses the obstacle field while maintaining a spherical formation and the minimum distance in three phases.

We conducted additional experiments in a real-world cluttered environment to further evaluate the effectiveness of our algorithm. As shown in Fig. 5a, the experiment involved nine Crazyflie drones, each equipped with four reflective markers for precise localization via the Nokov motion capture system. A central workstation processed the tracking data and communicated control commands to the swarm using Crazyradio PA. The layout of the flight arena and obstacle distribution is illustrated in Fig. 5b and Fig. 5c. The environment featured densely arranged obstacles, with the minimum gap between two objects measuring just 0.4 m. In this setup, the swarm flew at a speed of 0.4 m/s. As illustrated in Fig. 5d, the experiment included three obstacle-crossing tasks: (1) traversal in a square formation, (2) mid-flight transition from square to spherical formation, and (3) traversal entirely in a spherical formation. The corresponding trajectories and the minimum-distance curves also show that the spherical formation generates safer paths and keeps the robots farther away from obstacles compared with the square formation. Practically, this suggests that using a compact 3D formation can be advantageous for traversing highly constrained environments. The results demonstrate the capability of the proposed shape-assembly method to achieve reliable formation switching and safe navigation in highly constrained, real-world environments. Detailed visual record of the experiment can be found in Supplementary Video 6.

## Discussion

This article shows that bubble-raft-inspired shape assembly and formation-preserving obstacle avoidance together provide a feasible route toward scalable and autonomous aerial swarms. The balanced exploration controller uses only local sensing and neighbor communication, yet it drives hundreds of flying robots to fill complex three-dimensional shapes with nearly uniform density, recovers quickly when robots join or fail and supports rapid switching between formations. For obstacle traversal we steer the swarm by selecting safe velocity that deviates from the nominal shape-assembly direction and moving direction under velocity-obstacle constraints, which enable the swarm to fly through dense obstacles while keeping the overall formation recognizable. We also provide an additional demonstration of distributed large-language-model (LLM) deployment on the swarm, where collaborative inference across multiple onboard devices enables high-level planning for complex multi-stage missions^45–47^. Details are reported in the Supplementary Material Sec. 6 and Supplementary Video 7.

From a control perspective, the bubble-raft view suggests that assigning space rather than individual target points is a useful abstraction for large swarms. Mapping-based formation control builds one-to-one assignments and coupled trajectory plans, which quickly limits scalability as the number of robots grows^14–16,20^. Flocking and earlier shape-assembly methods rely on local interaction rules and scale more easily, but they do not explicitly regulate density and often produce uneven distributions that increase the risk of local crowding and collisions^25,26^. Voronoi-based decompositions have already been widely used in UAV navigation and region exploration to define collision-free operating regions, yet these regions are typically treated only as safe workspaces. In our framework the relative sizes of the regions become control variables: the controller continuously balances neighboring regions and links their volumes to local robot spacing^48–50^. In this way the method inherits the safety properties of Voronoi partitioning while also exploiting the partition to shape the spatial distribution of the swarm and achieve uniform formations.

Despite these advantages, several limitations remain. Because the bubble-raft mechanism itself admits small defects near boundaries, the final formations can still exhibit slight local non-uniformities^51,52^. Future work could add lightweight refinement steps to further smooth the distribution in these areas. The obstacle-avoidance experiments are carried out mainly with static obstacles under well-estimated states, whereas real environments may contain moving obstacles, stronger disturbances and sensing errors, and the present avoidance strategy will need to be extended and analyzed under such conditions.

## Methods

### The model

Our method is inherently distributed, as it relies on multiple agents that run in parallel and exchange information during each iteration. The robot swarm ${{{\mathscr{U}}}}$ consists of ${n}_{{\mbox{robots}}}$ robots distributed in ${R}^{3}$ space. The position of robot i is denoted as ${p}_{i}\in {R}^{3}$. When two robots satisfy $\left|{p}_{j}-{p}_{i}\right|\le {r}_{{\mbox{sense}}}$, they can establish a communication link and exchange positional information. The interaction set of robot i is defined as ${{{{\mathscr{U}}}}}_{{{{\mathcal{i}}}}}=\left\{j{{{\rm{\epsilon }}}}{{{\mathcal{U}}}}||{p}_{j}-{p}_{i}|\le {r}_{{\mbox{sense}}}\right\}$, where $\left|\cdot \right|$ denotes the Euclidean norm. The target shape G of the swarm is defined by an arbitrary distribution of target points: $G=\left\{{{tar}}_{i}{|i}={{\mathrm{1,2}}},\ldots ,{n}_{{\mbox{points}}}\right\}$ where ${n}_{{\mbox{points}}}$ is the number of target points and satisfies ${n}_{{\mbox{points}}}\ge {n}_{{\mbox{robots}}}$. At initialization, each robot stores the target shape G in the form of a k-dimensional tree (KD-tree), enabling efficient spatial indexing and target point querying within the ${r}_{{\mbox{sense}}}$ range.

We further define a Voronoi-style associate region for each robot. The associate region ${S}_{i}$ of robot i is the set of target points for which robot i is the closest robot:

1 $${S}_{i}=\left\{{{{\rm{tar}}}}\in {G|}\,{||}{{{\rm{tar}}}}-{p}_{i}{||}\le {||}{{{\rm{tar}}}}-{p}_{j}{||},\forall j\in {{{{\mathcal{U}}}}}_{{{{\mathcal{i}}}}}\right\}.$$

**Fig. 6:**
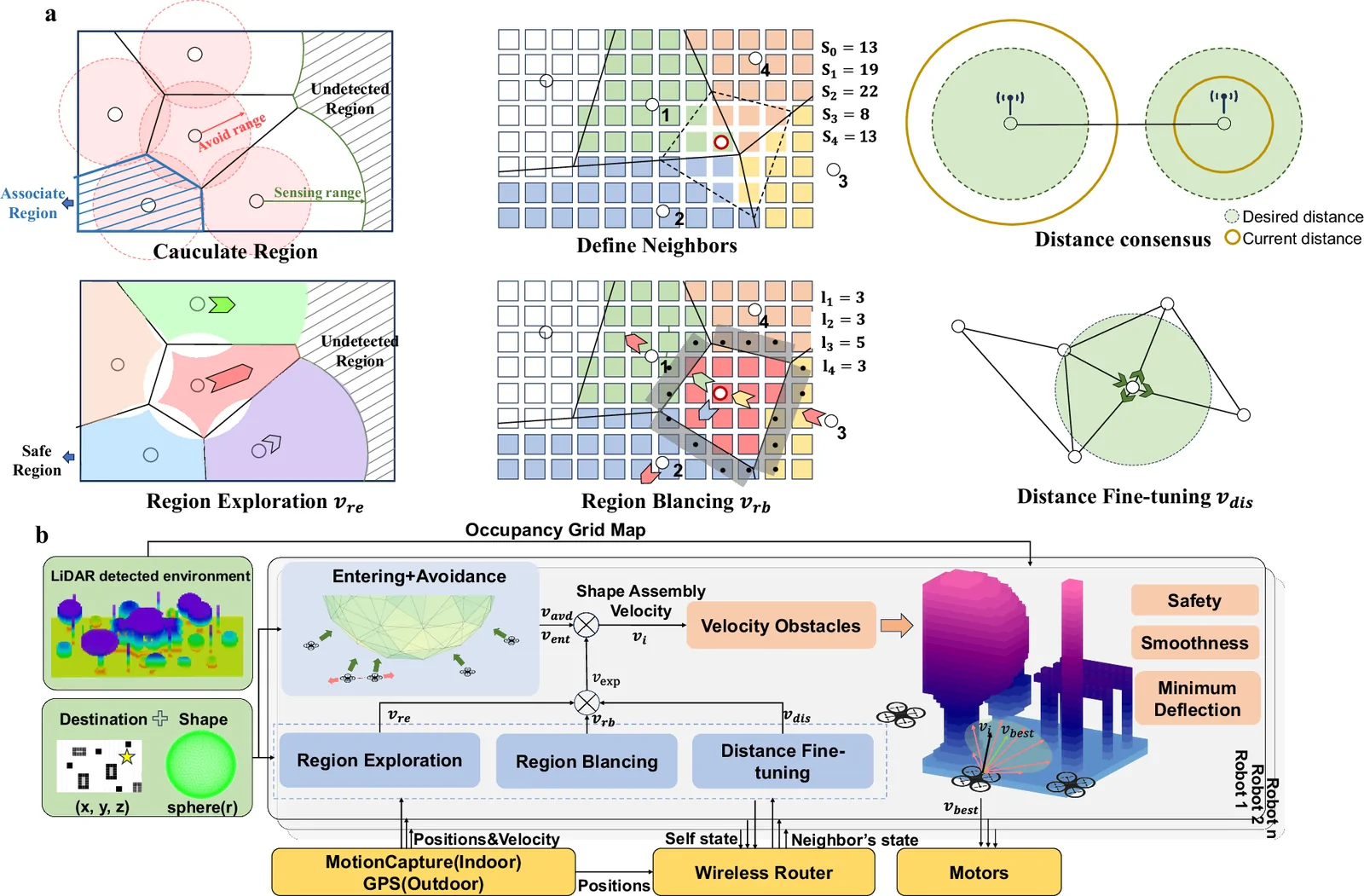
An illustration of the proposed shape-assembly strategy. **a** Mechanisms of region exploration(left), region-balancing(middle) and distance fine-tuning(right). **b** Robots outside the shape use ${v}_{{en}t}$ to approach the formation and ${v}_{{avd}}$ to avoid collisions. Inside the shape, ${v}_{{re}}$, ${v}_{{rb}}$, and ${v}_{{dis}}$ together form ${v}_{\exp }$, driving the swarm to distribute evenly. When obstacles are detected, robot evaluates candidate directions based on safety, smoothness, and turning angle to select the optimal motion.

In other words, each target point is assigned to the robot that is closest to it. Two robots are considered neighbors if their associate regions are adjacent. As illustrated in Fig. 6a, the closest robot within robot i’s interaction set to any target point in robot i’s associate region must be one of its neighbors. Accordingly, the neighbor set can be defined as:

2 $${N}_{i}=\left\{{{{{\rm{argmin}}}}}_{j\in {U}_{i}}{||}{p}_{j}-{tar|\,||tar}\in {s}_{i}\right\},$$

which means that for every target point ${{{\rm{tar}}}}$ in robot i’s associate region ${s}_{i}$, the robot in i’s interaction set ${U}_{i}$ that is closest to ${{{\rm{tar}}}}$ is treated as a neighbor of robot i. This is an implementation-level equivalent of defining neighbors as robots whose associate regions are adjacent.

### Forming the target shape

As shown in Fig. 6b, the shape-assembly velocity that drives the swarm to form the desired shape consists of five parts:

3 $${v}_{i}^{sh{ape}}={v}_{i}^{{ent}}+{v}_{i}^{{avd}}+{v}_{i}^{{re}}+{v}_{i}^{{rb}}+{v}_{i}^{{dis}}.$$

The first two terms mainly guide robots into the target shape without collisions, while the last three terms regulate how the swarm is distributed inside the shape. The entry command is designed to guide the swarm toward the target shape. The entry velocity for robot i is defined as:

4 $${v}_{i}^{{ent}}=\min \left({v}_{\max },{\mu }_{1}{||}{p}_{i}-{ta}{r}_{G}^{i}{||}\right)\cdot \frac{{ta}{r}_{G}^{i}-{p}_{i}}{{||ta}{r}_{G}^{i}-{p}_{i}{||}}.$$

Here, ${ta}{r}_{G}^{i}$ is the nearest target point to robot i in the formation graph G, and ${v}_{\max }$ is a predefined threshold that limits the maximum speed during entry. The gain ${{{{\rm{\mu }}}}}_{1}$ scales the influence of distance. The first term in the equation drives robot i into the formation. To further mitigate the risk of collision during the entry phase, a repulsive velocity is applied between robots i and j if their distance falls below ${r}_{{avoid}}$, which we set to twice the collision distance:

5 $${v}_{i}^{{avd}}=\frac{{r}_{{avoid}}}{{||}{p}_{i}-{p}_{j}{||}}-1.$$

To better drive the swarm to spread within the target shape, we design a region exploration velocity ${v}_{i}^{{re}}$ that makes each robot continuously move toward the center of its associate region. As shown in Fig. 6a, robots that enter the shape earlier initially own larger regions that extend into uncovered parts of the shape which helps the swarm gradually cover the entire target shape. The region exploration velocity is defined as:

6 $${v}_{i}^{{re}}={\mu }_{2}\left(\frac{{\sum}_{j\in {s}_{i}^{{safe}}}{w}_{i,j}\cdot {{{\rm{ta}}}}{{{{\rm{r}}}}}_{j}}{{\sum}_{j\in {s}_{i}^{{safe}}}{w}_{i,j}}-{p}_{i}\right),$$

where ${w}_{i,j}=\exp \left(-\frac{{\left|{{tar}}_{j}-{p}_{i}\right|}^{2}}{2{{{{\rm{\sigma }}}}}^{2}}\right)$ is the bandwidth of the Gaussian kernel, ${{{{\rm{\mu }}}}}_{2}$ is a given gain coefficient, and ${s}_{i}^{{safe}}$ is the safe subset of robot i’s associate region, defined as:

7 $${s}_{i}^{{safe}}=\left\{s\in {s}_{i}|\forall {n}_{j}\in {N}_{i},{||s}-{n}_{j}{||} > {r}_{{avoid}}\right\},$$

here, ${s}_{i}$ and ${N}_{i}$ denote the associate region and neighbor set of robot i, respectively, and ${r}_{{avoid}}$ is the avoidance distance. Using a Gaussian kernel gives higher weights to nearby target points, effectively suppressing the influence of outliers and improving the stability of convergence. Moreover, restricting the computation to the safe subset ${s}_{i}^{{\mbox{safe}}}$ keeps robot i from being attracted to target points that are too close to its neighbors, which reduces unnecessary activation of the repulsive velocity.

However, the region exploration command alone only yields a relatively loose distribution of robots inside the target shape, as illustrated in Fig. 1a. An additional mechanism is therefore needed to further enforce a more uniform distribution. In a bubble raft, each bubble, through local interactions, eventually spreads to occupy the surface with approximately equal size. These bubbles can be viewed as an analogy of the robots’ associate regions. By this analogy, achieving a uniform distribution of robots requires the sizes of all associate regions to be as equal as possible. This objective can be formulated as:

8 $${H}_{1}\left(U\right)={\sum }_{i\in U}{\left({S}_{i}-\bar{S}\right)}^{2},$$

where ${S}_{i}={card}\left({s}_{i}\right)$ represents the number of target points in the region assigned to robot i, and $\bar{S}={\sum }_{i\in U}{S}_{i}/{n}_{{robot}}$ denotes the average size of the regions. For robot i, changing its position only affects the size of its own region and those of its neighbors, so its optimization objective can be simplified as:

9 $${H}_{1}\left({p}_{i}\right)={\left({S}_{i}-\bar{S}\right)}^{2}+{\sum }_{j\in {N}_{i}}{\left({S}_{j}-\bar{S}\right)}^{2}.$$

It can be observed that when robot i moves, the change of the size of neighbor j’s associate region is mainly induced by the motion component along the shared boundary normal. Therefore, the variation of j’s region size can be approximated as $\frac{\partial {S}_{j}}{\partial {p}_{i}}\approx -\frac{{L}_{{ij}}}{2}{\bar{p}}_{{ij}},$ where ${L}_{{ij}}$ is the length of the shared edge and ${\bar{p}}_{{ij}}=({p}_{j}-{p}_{i})/\parallel {p}_{j}-{p}_{i}\parallel$. This is a local first-order approximation and we ignore higher-order geometric effects for simplicity. When evaluating the partial derivatives with respect to ${p}_{i}$, we only account for the effect of an infinitesimal displacement of robot i. This displacement redistributes boundary target points between ${S}_{i}$ and the neighboring regions ${S}_{j}$, the total size ${S}_{i}+{\sum }_{j\in {N}_{i}}{S}_{j}$ remains constant with respect to ${p}_{i}$, we have:

10 $$\frac{\partial {S}_{i}}{\partial {p}_{i}}+{\sum}_{j\in {{{{\mathscr{N}}}}}_{i}}\frac{\partial {S}_{j}}{\partial {p}_{i}}=0.$$

Substituting the above into Eq. (9) and simplifying yields:

11 $$\frac{\partial {H}_{t}}{\partial {p}_{i}}={\sum }_{j\in {N}_{i}}\left({S}_{i}-{S}_{j}\right){L}_{{ij}}{\bar{p}}_{{ij}}.$$

However, obtaining the exact value of ${L}_{{ij}}$ is computationally expensive, so we further simplify its calculation as:

12 $${L}_{{ij}}={{{\rm{card}}}}\left(\left\{{tar}\in {G}_{i}{{{\rm{| }}}}\exists j\in {N}_{i}{{{\rm{s}}}}.{{{\rm{t}}}}.\parallel {tar}-{p}_{i}\parallel -\parallel {tar}-{p}_{j}\parallel \in \left[0,{{{\rm{threshold}}}}\right]\right\}\right),$$

where card(·) denotes the cardinality of the set, and threshold is a given distance threshold, not smaller than the minimum distance between two target points. It is worth mentioning that Eq. (12) is a fuzzy calculation of the neighboring edge length, the denser the target point cloud, the smaller the threshold, and the closer the ratio of neighboring edge lengths to the true values among different neighbors. Therefore, the region balancing command ${v}_{i}^{{rb}}$ can be written as:

13 $${v}_{i}^{{rb}}={\mu }_{3}\frac{{\sum}_{j\in {N}_{i}}-({S}_{i}-{S}_{j}){L}_{{ij}}{\bar{p}}_{{ij}}}{{\sum}_{j\in {N}_{i}}{L}_{{ij}}},$$

where ${{{{\rm{\mu }}}}}_{3}$ is a given gain coefficient. This push–pull interaction between neighboring regions drives robots away from locally dense areas and toward sparsely populated ones, thereby improving the uniformity of the swarm distribution over the target shape.

In the bubble-raft analogy, the forces that drive the bubbles to form a uniform pattern arise not only from the bubble sizes but also from the distances between bubbles, eventually making them tightly packed with equal spacing. Inspired by this mechanism, we design a second objective for uniform distribution:

14 $${{{{\mathcal{H}}}}}_{2}\left({p}_{i}\right)={\sum}_{j\epsilon {N}_{i}}{\left({l}_{{ij}}-\bar{l}\right)}^{2},$$

where $\bar{l}={\sum}_{i\in U}{\sum}_{j\in {N}_{i}}{l}_{{ij}}/{\sum}_{i\in U}{{{\rm{card}}}}({N}_{i})$ denotes the average distance. Since the global average distance $\bar{l}$ is not directly available to each robot, we adopt a consensus mechanism. Each robot computes the average distance to all of its neighbors and exchanges this quantity with them. The updated estimate of the average distance for robot i is given by:

15 $$\bar{l}=\frac{{\bar{l}}_{i}+{\sum}_{j\in {N}_{i}}{\bar{l}}_{j}}{{{{\rm{card}}}}\left({N}_{i}\right)+1},$$

where ${\bar{l}}_{i}={\sum}_{j\in {N}_{i}}{l}_{{ij}}/{card}({N}_{i})$ denotes the local average distance of robot i. As the robots keep exploring the shape, these stored values converge, enabling each robot to obtain a consistent estimate of the global average spacing and to adjust its position accordingly. Based on the estimated average distance, each robot computes a distance-finetuning velocity:

16 $${v}_{i}^{{dis}}={\mu }_{4}{\sum}_{j\in {N}_{i}}({l}_{{ij}}-\bar{l}){\vec{p}}_{{ij}},$$

where ${{{{\rm{\mu }}}}}_{4}$ is a given gain coefficient. This velocity adjusts the spacing between robot i and its neighbor so that neighbor distances gradually converge to the average value. The convergence proof of the above algorithm is provided in Supplementary Information Section 2.

### Collision-free traversal method

Autonomous obstacle avoidance is a crucial technique for maintaining formation movement of the swarm in complex environments. We propose a lightweight, non-trajectory-planning-based obstacle avoidance scheme. To enable the swarm to move as a whole toward a common destination, we introduce a collective migration term ${v}_{i}^{{mig}}$ that is identical for all robots. This velocity drives the whole swarm to move with the same desired speed and direction.

During swarm motion, in order to avoid conflicts between the robot’s local target and obstacles, each robot computes its admissible velocity directions based on the positions of obstacles and neighboring robots within its sensing range. To prevent sudden deviations in motion direction, the admissible velocity directions are further constrained to lie within a cone centered on the current velocity direction, with an angular spread of 90°. Additionally, the robot’s sensing range is limited to ${{{{\rm{\mu }}}}}_{5}v$, where ${{{{\rm{\mu }}}}}_{5}$ is a gain coefficient and v is the current velocity magnitude. It prevents distant obstacles from increasing computational load when moving slowly, and also ensures timely avoidance when flying at high speed by avoiding too small a sensing range. Let B denote the obstacle set, which is constructed from the LiDAR point cloud together with the positions of other robots within the sensing range. The admissibility of a velocity direction can be determined using the following condition:

17 $${||}\vec{v}\times {\vec{u}}_{i}{||}\ge \frac{{r}_{{avoid}}}{{d}_{i}},$$

where ${\vec{u}}_{i}=\frac{{p}_{i}-o{b}_{j}}{{||}{p}_{i}-o{b}_{j}{||}}$, ${d}_{i}={||}{p}_{i}-{p}_{{ob}j}{||}$, and $o{b}_{j}\in B$. This constraint guarantees that the minimal distance between the straight-line path defined by $\vec{v}$ and obstacle $o{b}_{j}$ is at least ${r}_{{\mbox{avoid}}}$, directions that would lead to a closer approach are marked as inadmissible. To maintain the desired formation while avoiding obstacles, we define an evaluation function over all safe velocity directions, balancing the deviation from the desired velocity and from the previous velocity direction. For each safe direction $\vec{{v}_{s}}$, the score is calculated as:

18 $${Scor}{e}_{{v}_{s}}=\alpha \cdot \frac{{\vec{v}}_{s}\cdot {\vec{v}}_{i}}{{||}{\vec{v}}_{s}{||\,||}{\vec{v}}_{i}{||}}+\beta \cdot \frac{{\vec{v}}_{s}\cdot {\vec{v}}_{{last}}}{{||}{\vec{v}}_{s}{||\,||}{\vec{v}}_{{last}}{||}},$$

where ${{{\rm{\alpha }}}}$ and ${{{\rm{\beta }}}}$ are weighting coefficients,$\,{v}_{i}$ is the shape assembly velocity, and ${v}_{{last}}$ is robot i’s control velocity (not the real velocity) at the previous time step. The robot selects the direction with the highest score among all safe velocities as its new velocity direction ${v}_{{best}}$, while maintaining the same speed magnitude.

### Performance metrics

The effectiveness of shape formation is evaluated mainly through three metrics: Entry Percentage, Coverage Percentage, and Uniformity. Entry Percentage represents the proportion of the swarm that is located within the target shape, and is defined as:

19 $${Entry}\,{Percentage}=\frac{{n}_{{enter}}}{{n}_{{robots}}},$$

where ${n}_{{enter}}={{{\rm{card}}}}(\left\{i| {ta}{r}_{G}^{i} < {r}_{{avoid}}\right\})$, we use ${r}_{{\mbox{avoid}}}$ as the entry threshold for consistency with the avoidance clearance. Coverage Percentage measures how well the swarm covers the target shape, and is defined as:

20 $${Coverage}\,{Percentage}=\frac{{n}_{{cover}}}{{n}_{{tar}}},$$

where ${n}_{{cover}}={{{\rm{card}}}}(\left\{{tar}\in {G|}\exists {p}_{i}{{{\rm{s}}}}.{{{\rm{t}}}}.{||tar}-{p}_{i}{||}\le \bar{l}\right\})$, and ${n}_{{tar}}$ denotes the total number of target points. When both the entry percentage and coverage percentage reach 1, the swarm is considered to have successfully formed the target shape. Uniformity of the swarm is quantified using the coefficient of variation (CV) of the robots’ associate region sizes and the average neighbor distances, defined as:

21 $${Uniformity}={{CV}}_{{region}}+{{CV}}_{{distance}}=\sqrt{\frac{{{\sum }_{i=1}^{{n}_{{robots}}}({S}_{i}-\bar{S})}^{2}}{{n}_{{robots}}}}/\bar{s}+\sqrt{\frac{{{\sum }_{i=1}^{{n}_{{robots}}}({\bar{l}}_{i}-\bar{l})}^{2}}{{n}_{{robots}}}}/\bar{l}.$$

To characterize safety margins during formation and obstacle traversal, we also compute the Minimum Distance between robots and obstacles. Let ${d}_{{\mbox{robot}}}={\min }_{i\ne j}\parallel {p}_{i}-{p}_{j}\parallel$ be the minimum inter-robot distance, and ${d}_{{\mbox{obs}}}={\min }_{i,k}{||}{p}_{i}-{o}_{k}{||}$ be the minimum distance from any robot i to any obstacle point ${o}_{k}$. The overall minimum clearance is then defined as ${d}_{\min }=\min ({d}_{{robot}},{d}_{{obs}})$, where the term ${d}_{{\mbox{obs}}}$ is omitted in obstacle-free environments. We report ${d}_{\min }$ and compare it to the collision distance ${d}_{{\mbox{coll}}}$, collisions occur when ${d}_{\min } < {d}_{{\mbox{coll}}}$.

## Supplementary information

Transparent Peer Review file

Supplementary Information

Description of Additional Supplementary Files

Supplementary Video 1

Supplementary Video 2

Supplementary Video 3

Supplementary Video 4

Supplementary Video 5

Supplementary Video 6

Supplementary Video 7

## Data Availability

The data used in this study is publicly available at https://doi.org/10.5281/zenodo.18925237.

## Code availability

The code used in this study is publicly available at https://doi.org/10.5281/zenodo.17921956.
