# Supplementary material for: Bubble-raft inspired shape-assembly in flying robot swarm for uniform formation and obstacle traversal
Source: Commun Eng. 2026 Jul 2;5:178. doi: 10.1038/s44172-026-00715-3 (PMC13696712; doi:10.1038/s44172-026-00715-3)
Supplement: Supplementary file 1 [file 44172_2026_715_MOESM1_ESM.pdf]

nature portfolio

Peer Review File

Bubble-raft Inspired Shape-assembly in Flying Robot Swarm for Uniform Formation and Obstacle Traversal

Corresponding Author: Professor Biao Han

This file contains all reviewer reports in order by version, followed by all author rebuttals in order by version.

Version 0:

Reviewer comments:

Reviewer #1

(Remarks to the Author)
While I found this work interesting an relevant to the robotic swarm field, I felt that many of the methods used are not novel, and that the comapiron to similar exing approaches was lacking. I also felt the LLM component seemed disconnected and not well supported.

Some more detailed thoughts:

What advantage does this work give over other efforts to form shape without specific assignment? For example “Robust and Self-Repairing Formation Control for Swarms of Mobile Agents” does this type of behavior.

There is a lot of similarity to reynolds flocking with migration and local repulsion among other approaches, but no detailed comparison given

A lot of existing multi-robot behaviors using k-means for dispersion, for example: “Canopy Clustering Based Multi Robot Area Exploration” 2014

It would be good to know what capabilities are assumed the robots have before talking about algorithm. For example in the first technical section “control law of shape-assembly” you talk about robots using their position using robot-to-robot sensing, but i dont know how you compute position in a global coordinate system.

In 167 the authors talk about balanced allocation of space, but I dont see any evidence that this is the case. In my past experience, these approaches can end up with an un-equal distribution of area.

The part about using LLM to generate shapes seems pretty disconnected from the rest of the paper and like a last minute addon without much detail or review.

Reviewer #2

(Remarks to the Author)
The paper presents a novel and interesting shape-based formation control strategy for large robot swarms. The idea of decoupling the number of robots from the number of target points addresses a critical issue with scalability in swarm robotics. The bio-inspiration from bubble rafts provides an intuitive physical analogy for the proposed control directives, particularly for achieving uniform distribution.

However, I would like to highlight several questions and issues that should be addressed in the full paper.

Terms Definition
1. It is a bit confusing why the obstacle-avoidance directive is written as v_avg since “avg” subscript does not reflect obstacle avoidance. Was it supposed to be v_avd as in eq. 7?
2. “Avoidance range” is not well defined. It is unclear how exactly it is determined and if it is a fixed or variable parameter.

How is the boundary handled for target points that could be associated with multiple robots? In 155-156, it is mentioned that the regions assigned to robots are non-overlapping; however, in Fig. 1(b) it seems like they do overlap. Are “bubbles” and “associate regions” not the same?
3. As I understood, the bubble potential defined in Section 1 in Supplementary Information is fed into the global optimisation objective in eq. 10. What is the density of the solution when applied to the swarm?

Methodology
1. It is mentioned that the v_kms directive requires each robot to scan all target points within its sensing range. What is the computational complexity of this step? How does the performance scale as the density of target points increases?
2. Can the system get stuck in local minima, similar to the "point defects" mentioned in the bubble raft analogy? The paper admits to observing this in simulations. How are these situations handled? Is there a mechanism to escape them, or are they accepted as a limitation?
3. According to Section 2 in Supplementary Information, the Gaussian-weighted assignment is chosen over the standard K-means hard-partitioned centroid. How is the Gaussian σ parameter chosen? Is the system's performance sensitive to this value?
4. Convergence analysis in Section 2 in the Supplementary Information is conducted with a single-integrator model. However, the experiments use Crazyflie drones, which are usually modelled with double-integrator dynamics. Did you use a single-integrator model in the experiments as well? If not, how does it affect stability?
5. 258: What does "digit-level similarity errors" mean in a quantitative sense?
6. 259-260: it is stated that the shape was anchored with four virtual robots for the Graph Similarity method to handle scale and orientation. Is this a standard practice for this method? If it's a modification, it needs to be clearly justified. Are there other ways to go around this limitation on scale and orientation?
7. The main algorithm takes advantage of its distributed nature, which is inherently robust to the loss of individual robots. Introducing a single leader equipped with the LLM re-introduces a single point of failure. What is the contingency plan if the leader robot fails? Can another robot be elected as the new leader?
8. Running an onboard offline LLM on a small drone such as Crazyflie with very limited computational resources sounds unfeasible. How can it be implemented in real life?

Reviewer #3

(Remarks to the Author)
The proposed method is potentially very interesting, but unfortunately the current writing of the Results and Methods sections makes it impossible to assess the method adequately.

Major comments

The current methods section does not seem to describe a k-means algorithm (being “inspired by k-means” is not the same and is not an adequate reason to call the method a k-means method). Therefore, the authors should do one of the following:
1) Either, if the proposed method does not in fact use a k-means algorithm, the descriptions of the work throughout the paper should be updated accordingly (ideally, all mentions of k-means should be removed if k-means is not in fact used).
2) Or, if the proposed method does indeed use a k-means algorithm, the description of the methods needs to be drastically improved. In this case, the authors also need to add a comparison with a related state-of-the-art method, such as a Voronoi-based method, e.g.:
- Hu J, Wang M, Zhao C, Pan Q, Du C. Formation control and collision avoidance for multi-UAV systems based on Voronoi partition. Science China Technological Sciences. 2020 Jan;63(1):65-72.
- Hu J, Niu H, Carrasco J, Lennox B, Arvin F. Voronoi-based multi-robot autonomous exploration in unknown environments via deep reinforcement learning. IEEE Transactions on Vehicular Technology. 2020 Oct 29;69(12):14413-23.
* Inoue D, Ito Y, Yoshida H. Optimal transport-based coverage control for swarm robot systems: Generalization of the voronoi tessellation-based method. IEEE Control Systems Letters. 2020 Nov 18;5(4):1483-8.

The sections “Shape based formation control” and “Control law of shape-assembly” do not describe results and should not be in the Results section. They describe methods. A high-level intuitive description of these methods can be included in the introduction section, but the majority of the description should be relocated to the Methods section (and when redundancies occur, the redundant text should be removed).

In the section “Comparison with State-of-the-art Methods”, far more details are needed explaining how the existing methods were implemented. For example, the existing Mean-shift method [33] does not use target points in the same way as the proposed method, and the Image moment method [13] does not seem to use target points at all. How were target points combined with these two existing methods such that a fair comparison can be made to the proposed method? Without the implementation details of the existing methods, the results shown e.g. in Figure 3 cannot be adequately assessed. Furthermore, the metrics used in the section “Comparison with State-of-the-art Methods” are not explained anywhere.

The main results given in the Results section are in navigation scenarios, but the navigation portion of the proposed methods is not clearly described in the Methods section and none of the existing methods compared to in the section “Comparison with State-of-the-art Methods” are navigation methods. Also, there are no metrics for assessing navigation performance described in the Methods section. This is a major weakness of the paper, as there are many, many excellent formation control approaches for navigation in the literature. If the main results given in the paper are in navigation scenarios, then a performance comparison needs to be made to existing formation control approaches for navigation.

The Results section also mentions many other parts of the proposed method that are not at all described in the Methods section, such as the LLM-equipped leader robot. Without details about what has been done here, the quality and importance of the results cannot be adequately assessed. (Note: the supplemental information provided does not resolve this problem.)

The Results section also lacks adequate explanation of how the results are assessed:
- In sections “Collision avoidance and formation keeping in unknown environment” and “Flying through obstacles in real world”, the performance does not look better than many, many existing formation control approaches for navigation and existing collision avoidance approaches in the literature. How do these results demonstrate a scientific contribution to the state of the art?
- The section “Flying through obstacles in real world” does not adequately explain why the results should be considered valuable. For example, why is the fact that your method performs better with a sphere formation than with a square formation an interesting or important result?
- The section “Autonomous Swarm Control via LLM-Integrated Shape Assembly Method” does not provided any performance metrics by which the results can be assessed.

The Methods section is not adequately clear. Many of the variables in this section are not defined. The reader can eventually understand most of them from context, but they should all be explicitly defined. This should be corrected, throughout the section. The section is also missing intuitive textual descriptions of many of the equations. Such descriptions should be provided to the reader for all equations that are important to the paper's main contributions. (Some of this problem might be resolved by integrating the methods information that is currently incorrectly located in the Results section, but this will not resolve the problem completely.) This section also has a lot of inconsistencies in textual terminology, where different names are used to refer to the same thing, or the same name is used to refer to different things. All such inconsistencies need to be corrected.

After making all of the above revisions, the Introduction section and abstract should also be revised accordingly.

Supplemental line-by-line comments to help the authors improve the Results and Methods sections:

- 231: These metrics are not related to the performance metrics defined in the Methods section
- 237: Inter-robot distance is not the only component of the Uniformity metric described in the Methods section
- 248: If the graph similarity method was applied with only 100 robots, why are there results shown for the graph similarity method at 200 and 500 robots in Fig 3g ? Extremely confusing.
- 370: No tracking method is described in the Methods section
- 429: Is it exclusively distributed? Need to clarify
- 441: How is a formation graph “explored”?
- 442: Confusing that the definition of "neighbor" is different from the definition of robots with which there is a communication link. Eq 4 also does not seem to match this definition precisely.
- 448-449: This is a way to identify some neighbors, but not all neighbors, according to the earlier definition of "neighbors". This needs to be clarified.
- 452-453: Definitions of "target shape" and "formation area" are not given. Are they the same? Different?
- 459: How is the global velocity of the formation estimated? And is the estimation method distributed?
- 460: Why "during group flight" when this section is about a robot outside the formation area entering the formation area?
- 463: What is described in this section is not a k-means algorithm. Why are the authors calling this k-means exploration?
- 464: Why "further"? Is there spreading occurring during the entry phase?
- 465: Notation of v_kms does not match eq. 8
- 470: The "assigned region" value is called "ownership region" in the previous section
- 472-474: This seems to only be true under certain density conditions (density of robots and density of target points). This should be explained/justified.
- 475: This title is an ungrammatical phrase
- 477: Clusters of what? Clusters have not been referred to in the Methods section before now.
- 478: The term v_kms does not diffuse. What is meant here?
- 480: "assigned region" is called "ownership region" above
- 482: the absolute value notation is not used in eq. 10
- 487-490: This seems like it would only be true under very specific conditions of adjacent regions. Needs to be better explained/justified
- 491: Their combined size will not always remain constant when the robots are moving (i.e.., the region of the neighbor of robot i can change along its adjacency to a different one of its neighbors, not only along its adjacency to i). This statement about remaining constant needs more explanation/justification.
- 506: why sequential?
- 508: Is this formation graph different from the graph G stored at initialization? Are the robots then not trying to occupy a position in graph G? Very confusing; needs better explanation.
- 512: Should already mention that the global average distance is estimated using a consensus mechanism
- 516: This does not seem to be a correct use of "diffusion", as a technical term
- 533: How are the next positions predicted?
- 535: An intuitive textual description of the condition should be given to the reader, in addition to the equation.
- 542: The term "shape assembly velocity" has not been introduced

- 547-548: Avoid calling a metric a "rate" when there is no unit of time incorporated. It is confusing for the reader. If you were calculating the proportion of robots or the proportion of target points per a unit of time, it would be okay. But since there is no unit of time, it is confusing. These two names should be changed to better reflect the metrics.
- 551: Predefined as/according to what? Some details should be given.
- 557: What are the regions? Does this mean the ownership regions? Inconsistent terminology.

Comment about data and code:

The data needs to be publicly available in a permanent database, such as Zenodo or Dryad. Making the code available on GitHub is great. However, since GitHub entries are not necessarily permanent, the code should additionally be made publicly available in a permanent database, such as Zenodo.

Version 1:

Reviewer comments:

Reviewer #2

(Remarks to the Author)
The authors have addressed the comments raised in the previous round of review and conducted satisfactory revisions to the methodology and the additional experiments. However, there are still some issues that need to be resolved.
1. The description of the tensor-parallel partitioning in the MLP block appears to contain an inconsistency. The text states that W_{down} is partitioned column-wise and the results are concatenated. However, in standard tensor parallelism (e.g., Megatron-LM), the second linear layer (W_{down}) is typically partitioned row-wise, and the results are summed (all-reduce), not concatenated, because the input $h^{(p)}$ is already split across the feature dimension. Let's say if $h^{(p)}$ is a partial vector (e.g., size 100) and $W_{down}^{(p)}$ is a column-split matrix (e.g., size Total $\times$ 100), the dimensions don't align for a standard dot product representing the full transformation. If W_{down} is indeed column-split, please clarify how the matrix multiplication dimensions align given that $h^{(p)}$ is a local shard.
2. The manuscript suffers from numerous grammatical errors and awkward phrasing (e.g., 'compare with' instead of 'compared with', 'target not arrive', missing articles). The authors must perform a thorough proofread to correct these language issues before final publication.
3. Several acronyms are used throughout the manuscript without being defined upon their first appearance. For example, while terms like "MLP" are widely recognized in the field, standard academic practice requires that they be formally defined when first introduced. Please carefully scan the text to ensure all abbreviations are spelled out initially to improve accessibility for a broader audience.
4. Caption of Fig 2: "Formation for a cartoon mouse shape using 500 robots under our method, Mean-shift, and Image Moment.(More comparison can be found in Supplementary Video 4)". It seems like you forgot to add the Voronoi Partition.

Reviewer #3

(Remarks to the Author)
I provided a quite long list of comments in the previous round, and the authors have been very thorough in addressing all of my comments. I would like to commend the authors on the quality of their revision. All of my comments have been addressed and I am satisfied with the revision.

I found a few remaining issues:
- The text added at line 202 is nonsensical and needs revision.
- For the code and data repository: At the DOI the authors have provided, the code is available (this is good), but not the data. All experimental data should also be made publicly available in a permanent database (such as Zenodo or Dryad).
- There are some minor typos throughout the paper. Please conduct a thorough proofread.
- In all of the figures, most of the text is far too small. Please increase the font sizes in the figures so that the text can be read easily.
- At lines 300-305, several times you have written "large model" when you meant to write "large-language model".
- The sections on the application of LLMs are much improved in terms of technical soundness. However, they are very tangential to the main topic of the paper, and they do not report any results that are relevant to understanding the performance of the proposed "bubble-raft" algorithm. Therefore, I recommend moving these sections to the supplementary materials.

Version 2:

Reviewer comments:

Reviewer #2

(Remarks to the Author)
The authors have thoroughly addressed my previous comments, and I have no further concerns or suggestions.

Reviewer #3

(Remarks to the Author)
All of my comments have been addressed and I am satisfied with the authors' revision. I support publication of the manuscript.

Open Access This Peer Review File is licensed under a Creative Commons Attribution 4.0 International License, which permits use, sharing, adaptation, distribution and reproduction in any medium or format, as long as you give appropriate credit to the original author(s) and the source, provide a link to the Creative Commons license, and indicate if changes were made.
In cases where reviewers are anonymous, credit should be given to 'Anonymous Referee' and the source.
The images or other third party material in this Peer Review File are included in the article's Creative Commons license, unless indicated otherwise in a credit line to the material. If material is not included in the article's Creative Commons license and your intended use is not permitted by statutory regulation or exceeds the permitted use, you will need to obtain permission directly from the copyright holder.
To view a copy of this license, visit https://creativecommons.org/licenses/by/4.0/

All modifications in the revised manuscript are highlighted in red for clarity and ease of review.

Reviewer #1 (Remarks to the Author):

Comment: While I found this work interesting and relevant to the robotic swarm field, I felt that many of the methods used are not novel, and that the comparison to similar existing approaches was lacking. I also felt the LLM component seemed disconnected and not well supported.

Answer: Thank you for the thoughtful comments and for taking the time to review our paper. We appreciate your concerns about novelty, the strength of the comparisons, and the presentation of the LLM component. In response, we have carefully revised the manuscript and implemented detailed changes to address each point. A point-by-point reply with the specific modifications can be found below, including clarifications, expanded discussion, and additional supporting results.

Some more detailed thoughts:

Comment 1: What advantage does this work give over other efforts to form shape without specific assignment? For example "Robust and Self-Repairing Formation Control for Swarms of Mobile Agents" does this type of behavior.

Answer: We appreciate the reviewer's insightful comment and the pointer to "Robust and Self-Repairing Formation Control for Swarms of Mobile Agents" (SHAPEBUGS). This work is indeed an important precursor on assignment-free shape formation using a contained-gas model in a 2D domain. Our method is inspired by this line of work but targets a different regime and adds several capabilities that are important for aerial swarms.

First our method explicitly first guarantees **uniform spatial distribution** of agents within the target geometry rather than only maintaining the global contour, while the contained-gas rules in SHAPEBUGS disperse agents within the container and equalize a notion of "pressure", the resulting spacing is an emergent consequence of the repulsive radius and cannot be directly specified or regulated. Second, our framework generalizes seamlessly to **both two- and three-dimensional formations**, whereas SHAPEBUGS typically constrain the formation to planar shapes. Third, SHAPEBUGS is evaluated in an obstacle-free, periodic 2D environment and focuses on static shape formation and self-repair, the shape does not need to migrate through obstacles. In our work, the same shape-assembly controller is coupled with a velocity-obstacle-based local planner, enabling the **3D formation to traverse dense obstacles** while preserving its internal geometry and safety distances

To clarify these distinctions, we have added explanations in the Introduction.

Lines 56-58:

Cheng et al.[36] proposed a gas model inspired algorithm, which used dispersion model to form arbitrary shapes and could repair broken structures after agent failures, although the generated patterns were limited in precision and uniformity.

Line 66-68:

Overall, existing shape-assembly methods still lack explicit mechanisms to ensure uniformly filled formations and to enable swarm to move safely through dense environments using controllers of sufficiently low complexity.

We further conducted a systematic review of papers focus on shape generation methods without explicit assignment of robots to target points. For each work we recorded key attributes including whether it is can form specific formation, the scale of the swarm, the uniformity of the final formation, whether contains obstacle avoidance methods, the ability of self-healing. The resulting comparative table summarizes these distinctions and highlights the gaps our method addresses. The results and analyses can be found in **Supplementary Material Section 1.2 Table 1.**

**Paper**	**method**	**specific formation**	**large scale**	**uniformity**	**obstacle traversal**	**self-healing**
[1-2]	mean-shift	√	√	×	×	√
[3]	mean-shift	√	√	√	×	×
[4-5]	flocking	×	√	×	√	√
[6]	flocking*	√	√	×	×	√
[7-9]	Local swapping	√	√	√	×	×
[10]	Image moment	√	√	×	×	√
[11]	NMPC	×	×	×	√	×
[12]	reaction-diffusion	√	√	×	×	√
[13]	artificial light field	√	√	√	×	×
[14]	Voronoi	√	×	×	√	×
[15]	Voronoi	√	×	×	√	√
[16]	Trajectory-planning	√	×	√	√	×
[17]	Trajectory-planning	√	√	√	×	×
**Ours**	**bubble-raft**	√	√	√	√	√

*Example article given by reviewer

Comment 2: There is a lot of similarity to reynolds flocking with migration and local repulsion among other approaches, but no detailed comparison given

Answer: We thank the reviewer for this valuable comment. Indeed, flocking methods such as Reynolds-type control allow a swarm to maintain inter-agent distances and exhibit coordinated motion similar to bird flocks. However, classical flocking generally focuses on velocity alignment and local repulsion, which helps avoid collisions in obstacles environment but cannot maintain or reconstruct a prescribed global shape without additional formation mechanisms.

The shape generation method proposed in this article does indeed share some similarities with flocking in certain aspects, mainly due to the following points:

(1) The **Region Balance** and **Distance Fine-tuning** term can be interpreted as the tendency of adjacent robots to approach or retreat from one another, similar to the attractive and repulsive forces observed in flocking. However, these forces are generated by different mechanisms. The region balance force arises from disparities in the sizes of agents' ownership regions and drives agents to reallocate space until those regions become more even. The distance fine tuning force is produced by a distributed consensus protocol that yields a negotiated target inter-agent spacing which agents then track. This differs from flocking where attraction and repulsion are typically defined by fixed and predefined distance.

(2) The **Region Exploration** term has no direct analogue in Reynolds-type flocking. Instead of using only neighbor positions and velocities, each agent computes the kernel-weighted centroid of its Voronoi-like ownership region in the desired shape and moves toward under-covered

portions of that region. Classical flocking methods, in contrast, do not contain such a shape-aware exploration mechanism. They maintain a cohesive group and avoid collisions, but will not reconstruct a prescribed global shape from arbitrary initial conditions without an additional shape potential or assignment layer.

To provide a clearer comparison with flocking methods in obstacle traversal scenarios, we added an experiment with the GRF-based predictive flocking control, which characterizes inter-agent attraction/repulsion, alignment, obstacle avoidance, motion smoothness and region-attraction as potential energies in a Gibbs random field[1], uses mean-field approximations over predicted future states to form a posterior, and selects each agent's control input by maximizing this posterior in a distributed fashion.

Detailed comparison and analyze can be found in **Fig. 4 and line 247–255:**

As shown in Fig 4a, we show the trajectories produced by the three methods when the average obstacle spacing is 2.75 m and 1.75 m, with our method the swarm bends smoothly around the obstacles and the formation stays roughly intact, while NMPC and GRF-based flocking produce more twisted paths and the group shape is harder to recognize.

line 265-268:

The GRF-based flocking method moves the robots through gradients generated by neighbors, obstacles and a soft shape potential. In dense obstacles this gradient pulls robots toward the same gaps around obstacles, the formation is easily distorted and robots crowd together, leading to small inter-robot distances and more collisions.

[1] Yu C, Zhang D, Zhang Q. GRF-based predictive flocking control with dynamic pattern formation[C]//2024 IEEE International Conference on Robotics and Automation (ICRA). IEEE, 2024: 16788-16794

Comment 3: A lot of existing multi-robot behaviors using k-means for dispersion, for example: "Canopy Clustering Based Multi Robot Area Exploration" 2014

Answer: We thank the reviewer for pointing this out. We agree that the idea of associate regions is common in multi-robot research, K-means is often used for discrete-space partitioning (e.g., in grid maps or point clouds), while Voronoi diagrams are widely applied for continuous-space partitioning. Many existing works utilize these partitions for area exploration, shape generation, or path planning. However, these k-means/Voronoi-based methods are typically designed to divide a workspace among robots so that each robot is responsible for a different sub-area, many prior methods use such non overlapping regions to let each robot operate independently, which naturally lowers collision risk. We keep that benefit and extend the idea so that regions can further guide exploration: robots assigned larger regions are given a broader exploration space and therefore contribute more to covering sparse areas, while robots with smaller regions follows the robot with larger area to balance the region size.

Regarding the specific work cited by the reviewer, *"Canopy Clustering Based Multi Robot Area Exploration"*, we view it as representative of k-means-style clustering for **2D area exploration**: canopy clustering is introduced to improve the efficiency of k-means when segmenting the map into exploration regions, and the main metrics are exploration time and coverage performance rather than

shape quality or formation uniformity.

To give a clearer comparison with region-partition based formation methods, we have added an experiment with Hu et al. [2] as mentioned by reviewer 3. Their method partitions the workspace into nonoverlapping Voronoi task regions, confines each UAV's movement to its assigned region, and uses conventional artificial potential fields together with a destination-switching scheme to resolve local equilibria and guarantee collision-free convergence. Relevant results can be found in **Fig 2 and line 197-202:**

Voronoi Partition successfully forms the desired shape, however a few robots in the central region still fail to reach their target points and the formation converges more slowly than with other methods. This is because the method partitions the space into Voronoi cells based on robots' position and relies on the exchanging targets between neighboring regions to resolve conflicts. These frequent exchanges cause large amounts of ineffective motion, and some robots are assigned to target points that face each other across a narrow gap, result in the slow convergence and the target not arrive.

line 205-207:

Voronoi Partition becomes increasingly inefficient as the swarm size grows, due to repeated global Voronoi partitioning and destination switching among neighboring robots that introduce substantial additional computation.

[2] Hu J W, Wang M, Zhao C H, et al. Formation control and collision avoidance for multi-UAV systems based on Voronoi partition[J]. Science China Technological Sciences, 2020, 63(1): 65-72.

Comment 4: It would be good to know what capabilities are assumed the robots have before talking about algorithm. For example in the first technical section "control law of shape-assembly" you talk about robots using their position using robot-to-robot sensing, but I don't know how you compute position in a global coordinate system.

Answer: We thank the Reviewer for this critical comment and apologize for not making these assumptions explicit in the previous version. In our framework, all robots share a common world frame in which the target formation (point cloud) is defined. Each robot is assumed to have access to its **own pose estimate** in this global frame, which is a standard assumption in flying robot swarm systems. In outdoor environments, this can be obtained using mature localization stacks such as GNSS fused with IMU and/or visual-inertial odometry; in our indoor experiments we use a motion-capture system, and in simulation we use the estimated pose provided by the simulator. We have clarified these assumptions in the Result's first section to explicitly state the sensing and communication capabilities of the swarm.

line 95-105:

Platforms and experimental setup

In all simulations and experiments each robot can estimate its own three-dimensional position, velocity and acceleration, and can exchange position and velocity information with neighboring robots within its sensing range via short-range wireless communication. The control law is implemented in a fully distributed manner, and the control input of each robot is computed from its own state, the states of its neighbors and the target shape without a centralized planner. We evaluate the proposed method both in high-fidelity simulation and on real hardware. In simulation we use Unreal Engine[44] with the AirSim framework[45], where each virtual robot is equipped with a 3D lidar

sensor to perceive nearby obstacles. In real-world tests we deploy up to 18 Crazyflie 2.1 micro quadrotors46 in an indoor arena, and use a Nokov motion-capture system to provide the position and velocity of each drone, which are then fed to the same distributed shape-assembly and obstacle-avoidance controller as in simulation.

Comment 5: In 167 the authors talk about balanced allocation of space, but I don't see any evidence that this is the case. In my past experience, these approaches can end up with an un-equal distribution of area.

Answer: We thank the Reviewer for raising this point and for sharing experience. We agree that in many region-based methods, simply introducing an exploration term does not necessarily lead to an equal distribution of area in practice. Inspired by the energy of a bubble raft at equilibrium (Supplementary Sec. 1), we define **uniformity** of a formation through two complementary quantities:

(i) the variance of the robots' ownership region areas S_i, and

(ii) the variance of the robots' average neighbor distances $\bar{l}_i$.

Concretely, we introduce the normalized uniformity measure:

$$Uniformity = CV_{region} + CV_{distance} = \sqrt{\frac{\sum_{i=1}^{n_{robots}}(S_i - \bar{S})^2}{n_{robots}}}/\bar{S} + \sqrt{\frac{\sum_{i=1}^{n_{robots}}(\bar{l}_i - \bar{l})^2}{n_{robots}}}/\bar{l}$$

where $\bar{S}$ and $\bar{l}$ denote the mean region area and mean neighbor distance, respectively. A perfectly balanced configuration corresponds to zero variance in both terms (all regions have equal area and all robots have identical average neighbor spacing), while larger values indicate non-uniformity. On this basis we define two potentials,

$$H_1(p_i) = \sum_{i \epsilon U}(S_i - \bar{S})^2 \text{ and } H_2(p_i) = \sum_{j \epsilon N_i}\left(l_{ij} - \bar{l}\right)^2$$

and design the Region Balancing and Distance Fine-tuning control terms as local gradient-descent directions that decrease $H_1$and H_2, respectively. In Supplementary Sec. 2.1 we provide a Lyapunov-based analysis showing that the combined evolution of the swarm decreases these potentials and converges toward configurations with equalized region areas and neighbor distances.

We report the evolution of region areas and neighbor distances over time and robots, and their coefficients of variation, for different formations and swarm sizes (see Fig.1d,e, Fig.2b,c in the revised manuscript and Supplementary Material Section 4). These plots show that the proposed controller rapidly reduces the dispersion in both region areas and neighbor distances and keeps them low throughout the motion, even in a large swarm.

Comment 6: The part about using LLM to generate shapes seems pretty disconnected from the rest of the paper and like a last minute addon without much detail or review.

Answer: We thank the Reviewer for this comment. We agree that in the first version the LLM-based component was only briefly described and not sufficiently motivated, which could make it appear as a last-minute add-on.

In the revised manuscript we have restructured the narrative so that the role of the LLM is clearly tied to the overall goals and challenges of the paper. In the Introduction we now explicitly state that an aerial swarm operating in realistic environments needs three complementary capabilities:

line 33-36:

1) to form task-specific spatial formations at scale according to the mission, 2) to move through obstacles while preserving these formations and avoiding collisions among robots and with the environment, and 3) to autonomously select and update its goals and formations in response to changing tasks and environmental conditions.

The bubble-raft-inspired shape-assembly controller and the formation-preserving obstacle-avoidance scheme address the first two capabilities. The LLM-based component is introduced precisely to address the third, and to tackle the practical challenge:

line 84-93:

While the first two capabilities focus on how a swarm flies, the third concerns how it decides what to do. With the rapid progress of large language models, it is becoming feasible for swarms to interpret high-level human instructions and autonomously plan missions41. Recent works have begun to use large models as centralized planners that map natural language commands to give formation or waypoints for whole robot groups, but these systems still rely on a single powerful model running off-board which is difficult to deploy on resource-constrained flying robots[42,43]. In this work we move toward a more distributed alternative by partitioning the large language model across the swarm and letting robots collaboratively perform inference through local communication, so that no single agent needs to host the entire model. As a result, the swarm can negotiate its own shapes and destinations through a decentralized LLM and carry out multi-stage tasks such as autonomous search and rescue.

We have also substantially expanded the technical description and evaluation of the LLM part. A dedicated section ("Partitioning Large Language Models in Robot Swarms" **line 504-526**) now provides a detailed tensor-parallel partitioning scheme that splits each weight matrix column-wise across robot groups and describes, step by step, how attention and MLP computations are carried out collaboratively with two communication rounds per Transformer block.

In the Results section **line 300-336 and Fig6**, we further add two sets of experiments to demonstrate that this component is more than a conceptual add-on. First, we evaluate distributed inference latency of StableLM-3B, Llama2-7B and Llama2-13B on 5 Raspberry Pi 4B boards and compare our tensor-parallel scheme against a widely used layer-partitioning baseline (Llama.cpp with MPI). These experiments show that, under realistic network conditions, our design roughly halves per-token latency while respecting the compute and memory limits of the robots. Second, we design a five-stage search-and-rescue scenario in AirSim where a 16-robot swarm is controlled via our shape-assembly interface by either Llama2-7B or Llama2-13B partitioned across the swarm. The LLM generates only high-level commands, while the underlying shape-assembly and obstacle-avoidance controller executes these commands on individual robots. We report stage-wise success rates over multiple trials, which show that the distributed framework can complete multi-stage missions and that larger models offer clear benefits for long-horizon planning and decision-making.

Reviewer #2 (Remarks to the Author):

Comment: The paper presents a novel and interesting shape-based formation control strategy for large robot swarms. The idea of decoupling the number of robots from the number of target points addresses a critical issue with scalability in swarm robotics. The bio-inspiration from bubble rafts

provides an intuitive physical analogy for the proposed control directives, particularly for achieving uniform distribution.

Answer: Thank you for your positive assessment and for recognizing the novelty and relevance of our shape-based formation control strategy.

Terms Definition

Comment 1: It is a bit confusing why the obstacle-avoidance directive is written as v_{avg} since "avg" subscript does not reflect obstacle avoidance. Was it supposed to be v_{avd} as in eq. 7?

Answer: We thank the Reviewer for pointing out this notational inconsistency. In the revised manuscript we have corrected the notation and now consistently use v_{avd} to denote the obstacle-avoidance velocity throughout the paper to avoid any confusion.

Comment 2: "Avoidance range" is not well defined. It is unclear how exactly it is determined and if it is a fixed or variable parameter.

Answer: We thank the Reviewer for pointing this out. In our method, the avoidance range is a fixed parameter: we set it to twice the collision distance.

line 409-411:

To further mitigate the risk of collision during the entry phase, a repulsive velocity is applied between robots i and j if their distance falls below r_{avoid}, which we set to twice the collision distance:

Comment 3: How is the boundary handled for target points that could be associated with multiple robots?

Answer: We thank the Reviewer for raising this question. In our method, associate regions are defined in a Voronoi-style manner: a target point is assigned to the robot that is closest to it. Formally, in the revised manuscript we now state this as**(line 383)**

$$S_i = \{\mathrm{tar} \in G \mid \| \mathrm{tar} - p_i \| \leq \| \mathrm{tar} - p_j \|, \forall j \in \mathcal{U}_i\}$$

with a deterministic tie-breaking rule so that each target point belongs to exactly one robot. In continuous space, points that are exactly equidistant to two or more robots lie on Voronoi boundaries and form a set of measure zero, so they do not affect the theoretical derivations. In the discrete point cloud used in our implementation, exact ties almost never occur due to floating-point coordinates, if they do, we break the tie by assigning the point to the robot with the smaller index.

Comment 4: In 155-156, it is mentioned that the regions assigned to robots are non-overlapping; however, in Fig. 1(b) it seems like they do overlap.

Answer: We thank the reviewer to point this out. In Fig. 7a (previous Fig. 1b), the red circles marked are actually composed of the Avoidance range. When a robot falls within this range, a repulsive force is generated to prevent collisions between them. What this figure intends to illustrate is that the target points within the obstacle avoidance range of a certain robot will be excluded from the areas of other robots, as if the other robots move towards these points, they will enter the obstacle avoidance range and have a higher risk of collision. To make this point clearer, we show the annotations in the figure in a clearer manner, as shown in **Fig7 in main paper.**

Comment 5: Are "bubbles" and "associate regions" not the same?

Answer: We thank the reviewer for raising this question. Indeed, "bubbles" and "associate regions" are essentially equivalent concepts in our framework. As illustrated in Equation (4) in Supplementary Material, the formation of bubble rafts primarily depends on two conditions that drive the potential to zero: (1) equal spacing between bubbles and (2) uniform bubble size. These two conditions directly correspond to the two optimization objectives in our method—namely, regional balance and distance fine-tuning. The region can be regarded as an irregularly sized bubble, and the size of the region is the size of the bubble. To make this point clearer, we have provided clarification in **line 111-117:**

In our method the target points play the role of the water surface, the robots act as bubble centers, and each bubble is abstracted as the region of target points owned by a robot. Following this analogy we introduce a region-balancing term and a distance fine-tuning term that regulate the size of these regions and the spacing between neighboring robots. Simulations show that the swarm arranges itself into an almost uniform packing similar to a bubble raft, and we observe comparable small irregularities caused by point defects near the boundary.

Comment 6: As I understood, the bubble potential defined in Section 1 in Supplementary Information is fed into the global optimization objective in eq. 10. What is the density of the solution when applied to the swarm?

Answer: We thank the reviewer for this insightful question. The bubble interaction potential defined in Section 1 of the Supplementary Information is inspirational rather than directly used in the optimization. In the physical bubble raft model, the solution density affects the magnitude of inter-bubble forces (both attraction and repulsion), but it does not change the equilibrium condition—i.e., the stable configuration still corresponds to the minimum of the total potential energy, regardless of the absolute density value.

In our swarm analogy, we do not explicitly model or require the density. Density is implicitly represented by the size of the robot's associate region (i.e., the number of target points in its region). A larger region implies lower local robot density, and vice versa. The gradient-based control terms (v_{rb} and v_{dis}) drive the system toward uniformity by balancing region sizes and neighbor distances, which is equivalent to redistributing robots from high-density to low-density areas. Thus, the exact value of density is neither computed nor needed in our method.

However, from the perspective of an experimental designer, since we can obtain all the information, including the number of robots in the cluster (the robots themselves are unaware of this), we can determine the final density. The final uniform density of the swarm can be estimated as:

$$\rho_{final} = \frac{Volumn\ of\ target\ shape}{Number\ of\ robots}$$

In summary, density is abstracted away by the region-based control structure, which captures the same redistribution behavior as the bubble raft system without explicitly modeling fluid density. We have added a clarification in **Section 1.1 of the Supplementary Material** to emphasize this point.

It should be noted that the bubble potential in Equation (4) is merely for physical intuition and design inspiration. We did not directly substitute the solution density from the physical model into the global optimization in this paper. In bubble model, the force magnitude may be under different density but the process of generating raft remain unchanged. In our control, "local density" is

implicitly represented by the size of the associated area of each robot (i.e., the number of target points within that area): the larger the associated area, the lower the local robot density, and vice versa. The control term achieves the redistribution from high density to low density by balancing the area size and the neighbor distance, and thus does not require explicit calculation or input of fluid density.

Methodology

Comment 1: It is mentioned that the v_{kms} directive requires each robot to scan all target points within its sensing range. What is the computational complexity of this step? How does the performance scale as the density of target points increases?

Answer: We thank the reviewer for raising this important computational concern. The computational complexity of the v_{re}(used to be called v_{kms} is now called v_{re}, which means region exploration's abbreviation) directive per robot is demonstrated in **Supplementary Material Sec 2.1**:

Remark on v_i^{re}: The term v_i^{re} is derived from the optimization objective of complete shape coverage using a Voronoi-based formulation. The computational complexity of this step is as follow:

$$O\left(\log M + \frac{M}{V} \cdot r_{\mathrm{sense}}^3\right)$$

where M is the total number of target points, V is the volume of the target shape, r_{sense} is the sensing range, M/V is the average density of target points. In practice, we use a KD-tree to efficiently query target points within the sensing range, reducing the overhead from linear scan to logarithmic in M plus the number of local points.

Thus, increasing target density (i.e., higher M for fixed V) does increase local computation, but only linearly within the sensing volume, not across the entire space. Moreover, to empirically evaluate the actual impact of sensing range and target density on computational efficiency, we conducted a series of simulation experiments. We systematically varied the number of target points M from 1000 to 100000, and the sensing range r_{sense} from 3m to 20m while measuring the average per-robot computation time per control iteration over 10 runs under each setting. All tests were performed on a single Intel i9-12700H core using our Python implementation with SciPy KD-tree. Results can be found in **Supplementary Material section 4.1.3 and Fig. 18**.

Target-point density sensitivity. We vary the total number of target points $M \in \{1000, 5000, 10000, 50000, 100000\}$ while keeping $r_{\mathrm{sense}} = 10\,\mathrm{m}$ fixed. As shown in Fig. 18(a), the per-iteration time increases from 0.00043 s (1k points) to 0.00579 s (100k points). This trend is consistent with the analysis that the dominant term is proportional to the expected number of local points within the sensing volume, i.e., scaling approximately linearly with the target-point density inside the sensing ball. In practice, across our experiments in main paper, we typically set the number of target points to approximately 5,000, which already provides a sufficiently dense discretization for uniform shape assembly. Even for large-scale swarms with up to 500 robots, 5,000 target points are enough to produce visually and quantitatively uniform formations

Sensing-range sensitivity. We vary the sensing range $r_{\mathrm{sense}} \in \{3,5,10,15,20\}$m while keeping $M = 10{,}000$fixed. Fig. 14(b) shows that the per-iteration time increases from 0.00048s at 3m to 0.00116 s at

20 m. This increase matches the expectation that the number of queried points grows with the sensing volume ($\propto r_{\text{sense}}^3$), while the KD-tree overhead remains logarithmic in M. Overall, the results confirm that v^{re}remains computationally lightweight, and its runtime is primarily governed by the local point count within the sensing range.

Comment 2: Can the system get stuck in local minima, similar to the "point defects" mentioned in the bubble raft analogy? The paper admits to observing this in simulations. How are these situations handled? Is there a mechanism to escape them, or are they accepted as a limitation?

Answer: We appreciate the reviewer for highlighting the issue of local minima. Point defects analogous to those in bubble rafts can appear in the final stage of assembly, yet they do not halt convergence or impair global uniformity. In our observations, these defects are local phenomena and are not common across the overall shape. Even when point defects occur, the overall coverage rate and spacing statistics remain stable, and the swarm continues to satisfy safety constraints. This behavior is consistent with physical bubble rafts, where local defects naturally arise without affecting macroscopic structural order. We acknowledge that eliminating all possible local minima in a fully distributed, target-point-based shape assembly framework remains an open challenge and demonstrate this challenge in **Discussion line 360-362:**

Because the bubble-raft mechanism itself admits small defects near boundaries, the final formations can still exhibit slight local non-uniformities[51,52]. Future work could add lightweight refinement steps to further smooth the distribution in these areas.

Comment 3: According to Section 2 in Supplementary Information, the Gaussian-weighted assignment is chosen over the standard K-means hard-partitioned centroid. How is the Gaussian σ parameter chosen? Is the system's performance sensitive to this value?

Answer: We thank the reviewer for the question. In our implementation, the Gaussian bandwidth σ is chosen relative to the sensing range r_{sense} (i.e., $\sigma = 0.2\, r_{\text{sense}}$), because σ directly controls how far target points can "influence" the robot when computing its local destination. A larger σ includes a wider neighborhood of target points (so the local destination is closer to the center of the associated region) and typically yields a slightly faster formation due to stronger aggregate attraction. A smaller σ emphasizes closer target points, resulting in more local guidance and slightly slower but stable progress.

Regarding sensitivity, our results indicate that performance is not strongly sensitive to σ over a broad range: varying α from small to large values produces similar final shape quality and convergence behavior. The main difference is transient—larger σ can speed up early-stage coverage, while very large σ may occasionally introduce rare local artifacts near non-convex regions (e.g., a small number of robots may end slightly outside the boundary). We demonstrate and quantify this behavior in **Supplementary Material Fig 19 and Section 4.1.4:**

We evaluate the sensitivity of the Gaussian kernel bandwidth σused in the Gaussian-weighted local destination computation. Fig 19 shows results with $\sigma \in \{0.5,1,2,4,8\}$(scaled w.r.t. the sensing range). Overall, the difference across σ is not significant: all settings lead to successful shape formation with similar final coverage and distribution quality, indicating that the system is not highly sensitive to the exact value of σ.

The main effect of σ is on the transient behavior. When σ is larger, a wider set of target points contributes to the local destination, so the destination becomes closer to the center of the robot's associated region. This usually produces a stronger pulling effect (because more contributing points are farther away), and the swarm can reach the target shape slightly faster. However, a very large σ may also make the robot influenced by points across a broader area of a non-convex shape, which can occasionally lead to small local inconsistencies. In our results, for example, when $\sigma = 4$ and $\sigma = 8$, we observe a rare case where one robot ends up slightly outside the target shape. Importantly, this behavior is uncommon and does not change the overall convergence trend or the final macroscopic performance. In all experiment, we usually set it to $0.2 * r_{sense}$.

Comment 4: Convergence analysis in Section 2 in the Supplementary Information is conducted with a single-integrator model. However, the experiments use Crazyflie drones, which are usually modelled with double-integrator dynamics. Did you use a single-integrator model in the experiments as well? If not, how does it affect stability?

Answer: We thank the reviewer for pointing out the modelling gap. We did not use a pure single-integrator model in experiments. In both AirSim and Crazyflie, our algorithm outputs real-time velocity setpoints, and the platform's built-in low-level controller converts these velocity commands into attitude/thrust (and finally motor commands) to track the desired motion. Therefore, the single-integrator model in Supplementary Material Sec. 2.1 is used to analyze the outer-loop kinematic behavior induced by velocity commands, rather than the full vehicle dynamics.

To address the double-integrator nature more explicitly, we also include an extension in the Supplementary Material that models the robot as a double-integrator and represents the onboard controller as a standard velocity tracking loop (e.g., PI-type tracking). The proof follows the same Lyapunov logic as Sec. 2.1: we use the same formation energy and augment it with additional terms capturing the velocity tracking error and the controller's integral state. With appropriate tracking gains, the resulting Lyapunov function is non-increasing, which shows that moving from the single-integrator abstraction to the practical double-integrator-with-velocity-tracking setting does not compromise stability. This is consistent with our AirSim and Crazyflie experiments, where the swarm remains stable and converges under real-time velocity control. For the detailed process, please refer to **Section 2.2 of the supplementary materials**.

Comment 5: 258: What does "digit-level similarity errors" mean in a quantitative sense?

Answer: We agree that the phrase "digit-level similarity errors" was ambiguous and not a quantitative metric. What we intended to convey is that the Image Moment baseline only reproduces the coarse outer contour, while missing fine interior structures, so the resulting formation is visually similar only at a rough, low-detail level rather than matching the target shape's detailed geometry. To avoid this unclear wording, we revised the sentence to explicitly state this limitation in main paper **line 194-196:**

The Image Moment method uses Legendre and pseudo-Zernike image moments to drive the robots toward the desired shape, however it mainly assembles the outer outline of the mouse and fails to capture its interior details.

Comment 6: 259-260: it is stated that the shape was anchored with four virtual robots for the Graph

Similarity method to handle scale and orientation. Is this a standard practice for this method? If it's a modification, it needs to be clearly justified. Are there other ways to go around this limitation on scale and orientation?

Answer: We thank the reviewer for raising this concern. We agree that our previous comparison with the Graph Similarity method cannot be made fully fair in our experimental setting, mainly for the following reasons. First, the Graph Similarity pipeline is essentially centralized and requires global graph construction and matrix operations; in our implementation it does not scale to 500 robots with reasonable computation time, so the comparison would effectively become "500 (ours) vs. 100 (baseline)", which is not an apples-to-apples evaluation. Second, Graph Similarity does not natively handle global scale and orientation, and we previously introduced anchoring with virtual robots to fix these degrees of freedom. This anchoring introduces extra overhead (additional displacement and time for rotation/scale adjustment) that is not part of the original method and makes the baseline disadvantaged in a way that is unrelated to formation quality.

Since we cannot guarantee a fair and reproducible large-swarm comparison under the same assumptions, we remove the Graph Similarity baseline in the revised manuscript. Following the suggestion of Reviewer 3, we instead compare against Voronoi Partition, which is more closely aligned with our problem formulation.

In this method, the desired formation is defined as a set of N distinct destinations and a one-to-one (time-varying) assignment between UAVs and destinations. The workspace is partitioned into non-overlapping Voronoi task regions based on UAV positions, and each UAV is constrained to move inside its own region to guarantee collision avoidance. Motion is driven by an artificial potential field toward the assigned destination with repulsion, and a destination-switch scheme is used to break local equilibria by exchanging destinations under local rules. Importantly, we evaluate Voronoi Partition under the same 500-robot setting as our method, which ensures fairness and reproducibility. We conduct a more detailed explanation and comparison of this method in **main paper Fig 2 and line 197-202:**

Voronoi Partition successfully forms the desired shape, however a few robots in the central region still fail to reach their target points and the formation converges more slowly than with other methods. This is because the method partitions the space into Voronoi cells based on robots' position and relies on the exchanging targets between neighboring regions to resolve conflicts. These frequent exchanges cause large amounts of ineffective motion, and some robots are assigned to target points that face each other across a narrow gap, result in the slow convergence and the target not arrive.

Comment 7: The main algorithm takes advantage of its distributed nature, which is inherently robust to the loss of individual robots. Introducing a single leader equipped with the LLM re-introduces a single point of failure. What is the contingency plan if the leader robot fails? Can another robot be elected as the new leader?

Answer: We thank the reviewer for highlighting this important robustness concern. We agree that our previous design (a single leader robot equipped with the full LLM) indeed introduced a potential single point of failure. In the revised version, we have removed the centralized leader-LLM architecture and replaced it with a distributed inference mechanism to preserve the robustness of the overall distributed swarm system.

Specifically, we partition the swarm into a fixed number of groups and shard the LLM parameters across groups (e.g., each group stores a subset of columns of the model weight matrices). During inference, each group computes its local partial results and exchanges the required intermediate tensors over wireless communication to assemble the full activations needed for the next layer. Importantly, each shard is replicated within a group (i.e., multiple robots in the same group hold the same parameter shard), so the failure of an individual robot does not stop inference: as long as at least one robot per group remains active, the computation and communication required for inference can continue. With this revision, the swarm no longer relies on a unique leader, so a "leader failure" case is no longer critical. Detail of this method can be found in main paper **Fig 7 and line 504-526.**

8. Running an onboard offline LLM on a small drone such as Crazyflie with very limited computational resources sounds unfeasible. How can it be implemented in real life?

We appreciate the reviewer's concern, which strongly motivated us to address the practical challenge of deploying offline LLM capability on resource-limited micro-drones. In the revised paper, we experimentally evaluate the proposed LLM partitioning strategy and group-style deployment scheme described above, to demonstrate feasibility in resource-limited onboard settings.

Specifically, we implement and benchmark our distributed on-board inference pipeline on low-cost embedded hardware. We deploy three representative offline models (StableLM-3B-4E1T, Llama2-7B, and Llama2-13B) on a cluster of five Raspberry Pi 4B devices to emulate group-level compute units in the swarm, and report the per-token decoding latency under different degrees of parallelization. The results quantify the computation–communication trade-off and show that the pipeline can run within a practical real-time loop when the model is partitioned across multiple devices. We further compare against an MPI-based baseline (llama.cpp with MPI) which pipeline-parallelizes inference across devices by splitting layers into stages and demonstrate that our layer-internal sharding achieves lower end-to-end decoding latency under Wi-Fi-style communication.

To demonstrate task-level practicality, we run a five-stage search-and-rescue mission with repeated trials and report success rates, showing that the distributed pipeline supports multi-stage reasoning and decision making in an end-to-end autonomous swarm workflow. Together, these experiments provide concrete evidence that our approach is implementable in real life by distributing both memory and computation across the swarm (or swarm-attached embedded compute units), instead of requiring an infeasible single-drone deployment. We have revised the manuscript accordingly in main paper **Fig 6 and line 300-336**.

Reviewer #3 (Remarks to the Author):

Comment: The proposed method is potentially very interesting, but unfortunately the current writing of the Results and Methods sections makes it impossible to assess the method adequately.

Answer: We thank the reviewer for the careful reading of our manuscript and for the valuable comments. In the revised manuscript, we have substantially improved the clarity and organization of these sections, and the detailed revisions are provided in the point-by-point response below.

Major comments

Comment 1: The current methods section does not seem to describe a k-means algorithm (being "inspired by k-means" is not the same and is not an adequate reason to call the method a k-means method). Therefore, the authors should do one of the following:

1) Either, if the proposed method does not in fact use a k-means algorithm, the descriptions of the work throughout the paper should be updated accordingly (ideally, all mentions of k-means should be removed if k-means is not in fact used).

2) Or, if the proposed method does indeed use a k-means algorithm, the description of the methods needs to be drastically improved. In this case, the authors also need to add a comparison with a related state-of-the-art method, such as a Voronoi-based method, e.g.:

- Hu J, Wang M, Zhao C, Pan Q, Du C. Formation control and collision avoidance for multi-UAV systems based on Voronoi partition. Science China Technological Sciences. 2020 Jan;63(1):65-72.
- Hu J, Niu H, Carrasco J, Lennox B, Arvin F. Voronoi-based multi-robot autonomous exploration in unknown environments via deep reinforcement learning. IEEE Transactions on Vehicular Technology. 2020 Oct 29;69(12):14413-23.

* Inoue D, Ito Y, Yoshida H. Optimal transport-based coverage control for swarm robot systems: Generalization of the voronoi tessellation-based method. IEEE Control Systems Letters. 2020 Nov 18;5(4):1483-8.

Answer: We thank the reviewer for pointing out this important issue. We agree that our previous wording was misleading: while part of our region-exploration term was motivated by the centroidal coverage objective that is written in a K-means–like form, our method does not execute a standard K-means algorithm (i.e., repeated global assignment and centroid-update iterations). In our implementation, target-point association is formed in a Voronoi-like manner, and each robot then computes a local Gaussian-weighted centroid direction to guide exploration. Therefore, describing the method as a "K-means method" is inaccurate. Following the reviewer's suggestion, in the revised manuscript we avoid calling the method K-means and update the terminology throughout accordingly (e.g., renaming the directive previously referred to as v_{kms} to the region-exploration term v_{re}).

In addition, following the reviewer's recommendation to compare against related Voronoi-based approaches, we include a Voronoi Partition baseline (Hu et al., 2020) in the revised experiments. We run this baseline under the same swarm scale (500 robots) and the same sensing-range settings as our method, ensuring a fair comparison. We show the comparison result in main paper **Fig 2 and line 197–202:**

Voronoi Partition successfully forms the desired shape, however a few robots in the central region still fail to reach their target points and the formation converges more slowly than with other methods. This is because the method partitions the space into Voronoi cells based on robots' position and relies on the exchanging targets between neighboring regions to resolve conflicts. These frequent exchanges cause large amounts of ineffective motion, and some robots are assigned to target points that face each other across a narrow gap, result in the slow convergence and the target not arrive.

Comment 2: The sections "Shape based formation control" and "Control law of shape-assembly" do not describe results and should not be in the Results section. They describe methods. A high-level

intuitive description of these methods can be included in the introduction section, but the majority of the description should be relocated to the Methods section (and when redundancies occur, the redundant text should be removed).

Answer: We thank the reviewer for this helpful organizational suggestion. We agree that the sections "Shape based formation control" and "Control law of shape-assembly" are methodological descriptions rather than results. In the revised manuscript, we have relocated these sections to the Methods part (keeping only a brief, high-level intuitive overview where appropriate), and we removed redundant text created by the re-organization. The Results section is now focused exclusively on demonstrating the proposed method's capabilities and reporting quantitative evaluations, including performance tests and baseline comparisons.

Comment 3: In the section "Comparison with State-of-the-art Methods", far more details are needed explaining how the existing methods were implemented. For example, the existing Mean-shift method [33] does not use target points in the same way as the proposed method, and the Image moment method [13] does not seem to use target points at all. How were target points combined with these two existing methods such that a fair comparison can be made to the proposed method? Without the implementation details of the existing methods, the results shown e.g. in Figure 3 cannot be adequately assessed.

Answer: We thank the reviewer for pointing this out. We agree that Mean-shift and Image Moment use different shape representations than our point-based controller, and that the implementation details must be clearly stated to assess fairness. In the revised manuscript, we added an explicit method input and parameter settings to clarify how each method is executed and how we compute the same evaluation metrics under a unified experimental setup.

The Mean-shift method we compare operates on a grid-based shape representation (gray-scale map of the target shape) rather than a continuous set of target points. We therefore use the authors' open-source implementation to quantize the binary mouse image into a grid of gray-scale cells and run the method using its native representation and update rule.

The Image Moment method directly takes the binary image as input and uses Legendre and pseudo-Zernike moments to generate control force, it does not rely on target points. Accordingly, we run this baseline exactly in its image-based form. For coverage, we account for each robot's finite footprint. At each time step, we mark target pixels within a fixed radius around each robot as covered, and report the covered-target-pixel ratio over time.

Voronoi Partition is inherently mapping based we uniformly sample 500 points inside the target shape as the destination set, matching the one-to-one robot-to-destination formulation.

These implementation details have been added to the revised manuscript in main paper **line 167–173:**

To validate our method's advantages in coverage of target shape, safety, uniformity and lightweight implementation, we compared it against three formation methods: Mean-shift[37], Image Moment[19], and Voronoi Partition[47]. For the Image Moment method, the target formation is given directly as the binary mouse image. For Voronoi Partition, we uniformly sample 500 points inside the black region of this image as target points. For Mean-shift, we use the authors' open-source implementation to quantize the binary image into a grid of occupied cells as the target formation, and we take the center of each occupied cell as a target point for our method. The sensing range is set to 5m in four methods.

Comment 4: Furthermore, the metrics used in the section "Comparison with State-of-the-art Methods" are not explained anywhere.

We thank the reviewer for pointing this out. In the revised manuscript, we explicitly define all evaluation metrics used in the "Comparison with State-of-the-art Methods" section in the Methods part, including **entry percentage**, **coverage percentage**, and the **uniformity** metric. We also clarify the previously ambiguous "time" measurement by rewriting it as **computing time per iteration**, which reports the average computation time required for one control iteration. These additions and clarifications are included in main paper **line 175-178:**

In these experiments we measure swarm uniformity using only the coefficient of variation of inter-robot distances, and we report coverage percentage, minimum distance in robot swarm and uniformity index over time in Fig2c.

Comment 5: The main results given in the Results section are in navigation scenarios, but the navigation portion of the proposed methods is not clearly described in the Methods section and none of the existing methods compared to in the section "Comparison with State-of-the-art Methods" are navigation methods. Also, there are no metrics for assessing navigation performance described in the Methods section. **This is a major weakness of the paper**, as there are many, many excellent formation control approaches for navigation in the literature. If the main results given in the paper are in navigation scenarios, then a performance comparison needs to be made to existing formation control approaches for navigation.

Answer: We thank the reviewer for raising this important concern. We agree that our previous "state-of-the-art" comparison focused on shape-assembly baselines and did not include navigation-oriented formation controllers, and we also did not clearly define navigation metrics. To address this major weakness, we added two navigation baselines and a dedicated navigation evaluation to match the scenarios used in our main results.

For navigation comparisons, we add the NMPC swarm controller of Soria et al. This baseline repeatedly plans over a short horizon by predicting the motion of each drone and a fixed number of its nearest neighbors using a discrete model whose state is position and velocity and whose input is acceleration. It then chooses the control sequence that minimizes a cost with separation, propulsion, direction, and control-effort terms, while enforcing safety-distance constraints to both neighboring drones and obstacles, and replans at the next step. We also add the GRF-based predictive flocking controller from Yu et al., which models robot–robot and robot–environment interactions using potential energies inside a Gibbs random field and computes the control by maximizing a posterior distribution, while using a region-based shape/pattern term with a mean-shift mechanism to spread robots inside the desired region. Since the method accept acceleration commands, we follow their practical implementation.

We then evaluated both baselines in the same obstacle-traversal settings as our main navigation results, using identical swarm size, sensing range, obstacle layouts, and initialization. In addition to formation-quality metrics, we added navigation-specific metrics in the Methods section, including traversal success rate in clutter, minimum robot–robot and robot–obstacle clearance over time, and computing time per iteration, so that safety, efficiency can be assessed together. Across randomized forest-like environments with varying obstacle spacing and flight speed, our method consistently

achieves higher traversal success while maintaining the desired formation shape and safe separation. In contrast, the NMPC baseline becomes substantially more expensive per iteration as swarm size increases, and the GRF-based flocking baseline tends to preserve cohesion but shows reduced shape fidelity in narrow passages, leading to lower coverage/uniformity during traversal. We include these comparisons and the corresponding metrics in main paper **Fig 4, line 242–276 and line 541-546**.

Comment 6: The Results section also mentions many other parts of the proposed method that are not at all described in the Methods section, such as the LLM-equipped leader robot. Without details about what has been done here, the quality and importance of the results cannot be adequately assessed. (Note: the supplemental information provided does not resolve this problem.)

Answer: We thank the reviewer for pointing out this gap. We agree that the previous version did not provide enough methodological detail for the LLM component mentioned in Results. In the revised manuscript, we remove the single "LLM-equipped leader" design and instead describe a distributed on-board inference approach, where the swarm is partitioned into multiple groups and the LLM parameters are sharded across groups. Each group computes partial intermediate results and exchanges the required tensors over wireless communication to reconstruct the full activations layer by layer, so that no single drone needs to host the full model.

We also add explicit evaluation metrics to make the LLM-related results assessable. For system feasibility, we report **per-token decoding latency** on low-cost embedded hardware and compare against a pipeline-parallel MPI baseline (llama.cpp with MPI). For task-level effectiveness, we evaluate the LLM in a **five-stage search-and-rescue mission** and report **success rates over repeated trials**. These method descriptions and metric definitions have been added to the **Result section (line 300–336) and Methods section(line 504-526)**.

The Results section also lacks adequate explanation of how the results are assessed:

Comment 7: In sections "Collision avoidance and formation keeping in unknown environment" and "Flying through obstacles in real world", the performance does not look better than many, many existing formation control approaches for navigation and existing collision avoidance approaches in the literature. How do these results demonstrate a scientific contribution to the state of the art?

We thank the reviewer for this important question. We agree that, without navigation-focused baselines, the obstacle-traversal demonstrations in the previous version do not sufficiently show how our performance compares to the state of the art. To address this directly, we added quantitative navigation comparisons against two representative formation-navigation approaches, namely the NMPC swarm controller of Soria et al. and the GRF-based predictive flocking controller of Yu et al., and evaluated all methods in the same obstacle environments.

In the revised experiments, we report navigation-relevant metrics, including **traversal success rate in dense obstacles**, **minimum robot–robot and robot–obstacle clearance**, and **computing time per iteration**. The new results show that our method maintains higher traversal success and safer minimum clearance in denser obstacle fields, while the NMPC baseline becomes significantly more expensive per iteration as swarm size increases and the GRF-based flocking baseline degrades in dense cases and exhibits larger formation distortion in narrow passages. These added comparisons are intended to directly justify the navigation claims relative to existing approaches, and we include them in **Fig 4 and line 242–276.**

Comment 8: The section "Flying through obstacles in real world" does not adequately explain why the results should be considered valuable. For example, why is the fact that your method performs better with a sphere formation than with a square formation an interesting or important result?

Answer: We thank the reviewer for this comment. Our intention in this section is to demonstrate two valuable points. First, the real-world flights validate the simulation results by showing that the proposed approach can achieve safe traversal in dense obstacle fields and can reliably execute online formation transitions, including switching between 2D and 3D formations. Second, the square-versus-sphere study provides a concrete formation-design insight for constrained navigation. As reflected by the trajectories and minimum-distance curves in Fig. 5d, the 3D spherical formation maintains larger clearance from obstacles than the 2D square formation in the same environment, suggesting that 3D formations can offer safer obstacle traversal and may be preferred in a wider range of dense real-world scenarios. We have revised the manuscript accordingly in **line 293–298:**

The corresponding trajectories and the minimum-distance curves also show that the spherical formation generates safer paths and keeps the robots farther away from obstacles compare with the square formation. Practically, this suggests that using a compact 3D formation can be advantageous for traversing highly constrained environments. The results demonstrate the capability of the proposed shape-assembly method to achieve reliable formation switching and safe navigation in highly constrained, real-world environments.

Comment 9: The section "Autonomous Swarm Control via LLM-Integrated Shape Assembly Method" does not provided any performance metrics by which the results can be assessed.

Answer: We thank the reviewer for pointing this out. In the revised manuscript, we add explicit metrics to quantitatively assess the LLM-related results. On the system performance side, we report the per-token latency with the scaling trend as the number of groups changes, which captures the computation communication trade-off. We have added the corresponding metric definitions in **line 547-550:**

To quantify the runtime feasibility of the distributed LLM, we report the per-token decoding latency. let T_{decode} denote the total time to generate N_{tok} output tokens. The average per-token decoding latency is defined as:

$$\text{Latency} = \frac{T_{\text{decode}}}{N_{\text{tok}}}$$

For a baseline comparison, we include llama.cpp with MPI, which implements a pipeline-parallel execution by splitting the model into sequential layer blocks across multiple devices and passing intermediate activations stage-by-stage along the pipeline. We compare our approach against this baseline under the same embedded hardware and Wi-Fi-style communication, and report the corresponding latency and throughput. On the task-level performance side, we evaluate the LLM in a five-stage search-and-rescue mission and report success rate over repeated trials (including stage-wise success), which directly measures whether the LLM-generated plans lead to correct multi-step swarm behaviors. We have added these results in the revised manuscript **line 305–336.**

Comment 10: The Methods section is not adequately clear. Many of the variables in this section are not defined. The reader can eventually understand most of them from context, but they should all be

explicitly defined. This should be corrected, throughout the section. The section is also missing intuitive textual descriptions of many of the equations. Such descriptions should be provided to the reader for all equations that are important to the paper's main contributions. (Some of this problem might be resolved by integrating the methods information that is currently incorrectly located in the Results section, but this will not resolve the problem completely.) This section also has a lot of inconsistencies in textual terminology, where different names are used to refer to the same thing, or the same name is used to refer to different things. All such inconsistencies need to be corrected.
Answer: We thank the reviewer for this thorough feedback, which was very helpful for improving clarity. In the revised manuscript, we have carefully revised the Methods section throughout. Specifically, we (i) explicitly define all variables upon first use, (ii) add intuitive textual explanations for all key equations supporting the main contributions, and (iii) resolve terminology inconsistencies by unifying notation so that the same concept is referred to consistently and no symbol is overloaded. We have also integrated the method descriptions that were previously misplaced in the Results section into the Methods section and removed redundancies. These line-by-line comments provided by reviewer have greatly guided our revisions.

Comment: After making all of the above revisions, the Introduction section and abstract should also be revised accordingly.
Answer: We appreciate this suggestion. Accordingly, we have revised both the Introduction and the abstract to align with the above changes and to ensure consistency with the updated manuscript.

Supplemental line-by-line comments to help the authors improve the Results and Methods sections:

- 231: These metrics are not related to the performance metrics defined in the Methods section
We thank the reviewer for noting that the metrics referenced in this part of the Results section did not correspond to the performance metrics formally defined in the Methods section. We have corrected this by aligning all Results-section metrics with their exact definitions in the Methods section. The revised text is now consistent throughout.
line 167-169:
To validate our method's advantages in coverage of target shape, safety, uniformity and lightweight implementation, we compared it against three formation methods: Mean-shift[37], Image Moment[19], and Voronoi Partition[47].

- 237: Inter-robot distance is not the only component of the Uniformity metric described in the Methods section
We thank the reviewer for pointing this out. Our uniformity metric is originally defined as the sum of two components, namely the coefficient of variation of the associate-region sizes and the coefficient of variation of inter-robot (neighbor) distances. However, for a fair baseline comparison, we report uniformity using only the distance-based term in the comparison section, because our method and Mean-shift can compute the region-size variation, while Voronoi Partition and Image Moment do not provide a consistent notion of "associate region" that allows computing CV_{region} in a comparable way. We have clarified this in the revised manuscript **line 175–177:**
In these experiments we measure swarm uniformity using only the coefficient of variation of inter-

robot distances since the Voronoi-based baseline doesn't provide an associate-region definition that enables consistent computation of region-size variation.

- 248: If the graph similarity method was applied with only 100 robots, why are there results shown for the graph similarity method at 200 and 500 robots in Fig 3g ? Extremely confusing.

We thank the reviewer for pointing out this confusion. In our earlier draft, the Graph Similarity method was practically infeasible to run to completion at 200 and 500 robots because the convergence time became prohibitively large (e.g., the 500-robot case required on the order of ~13 hours to form the final shape in our implementation). We therefore only executed full formation experiments with 100 robots, while the larger-swarm plots (computing time per iteration) were included from partial runs, which indeed makes the figure presentation confusing and the comparison unfair. In addition, Graph Similarity does not natively resolve scale and orientation, and our previous use of anchored virtual robots introduced extra displacement and time overhead, further confounding a fair comparison. For these reasons, in the revised manuscript we remove the Graph Similarity baseline and instead compare against a scalable Voronoi-based method under the same swarm size (500 robots), ensuring a clear and fair evaluation.

- 370: No tracking method is described in the Methods section

We thank the reviewer for pointing this out. In the previous version, we mentioned an additional target-tracking capability for the LLM-controlled swarm, but the corresponding tracking method was not described in the Methods section, which caused inconsistency. In the revised manuscript, we have removed the tracking task and related claims, and we now focus on the search-and-rescue scenario as the primary evaluation of the LLM-enabled swarm control capability.

- 429: Is it exclusively distributed? Need to clarify

We thank the reviewer for this question. Our approach is fully distributed in that each robot computes its control update using only local sensing and neighbor communication, without any centralized planner or global state. The core shape-assembly and obstacle-avoidance components rely on local target-point queries and local exchanges with nearby robots, and the LLM component is also executed in a distributed manner by sharding the model across robot groups and exchanging intermediate tensors during inference. We have clarified this in the revised manuscript **line 373–374:**

Our method is inherently distributed, as it relies on multiple agents that run in parallel and exchange information during each iteration.

- 441: How is a formation graph "explored"?

We thank the reviewer for pointing this out. In the previous draft, the phrase "exploration of the formation graph" was intended to describe the exploration of the target shape during formation, rather than any explicit graph-search procedure. We agree that "formation graph" has a strict graph-theoretic meaning and was an inaccurate term here. In the revised manuscript, we have replaced this wording throughout by consistently referring to target-shape exploration, and we clarified that robots only perform local neighbor communication based on adjacent associate regions. We have revised the text accordingly **(Line 391–398).**

Two robots are considered neighbors if their associate regions are adjacent. As illustrated in Fig 7a ,

the closest robot within robot i's interaction set to any target point in robot i's associate region must be one of its neighbors. Accordingly, the neighbor set can be defined as:

$$N_i = \left\{ \underset{j \in U_i}{\operatorname{argmin}} \|p_j - tar\| \mid tar \in s_i \right\} \quad (4)$$

which means that for every target point tar in robot i's associate region s_i, the robot in i's interaction set U_i that is closest to tar is treated as a neighbor of robot i. This is an implementation-level equivalent of defining neighbors as robots whose associate regions are adjacent.

- 442: Confusing that the definition of "neighbor" is different from the definition of robots with which there is a communication link. Eq 4 also does not seem to match this definition precisely.

We thank the reviewer for pointing out this confusion. In the previous version, we mistakenly used the term "neighbor" to refer to robots within communication range, while elsewhere "neighbor" meant robots whose associate regions are adjacent. In the revised manuscript, we have unified the terminology by consistently defining neighbors as robots with adjacent associate regions, and referring to robots within communication range as the interaction set. we have revised the text accordingly in **line 376-377:**

The interaction set of robot i is defined as $\mathcal{U}_i = \{ j \in \mathcal{U} \mid |p_j - p_i| \le r_{\text{sense}} \}$, where $|\cdot|$ denotes the Euclidean norm.

- 448-449: This is a way to identify some neighbors, but not all neighbors, according to the earlier definition of "neighbors". This needs to be clarified.

We thank the reviewer for pointing this out. This concern was due to our earlier misuse of "neighbors" to mean robots within communication range. In the revised manuscript, "neighbors" refers only to adjacent associate regions, and Eq.2 (previous Eq. 4) is written for this adjacency-based definition (with U_i as the interaction set used to find them).

- 452-453: Definitions of "target shape" and "formation area" are not given. Are they the same? Different?

We thank the reviewer for pointing this out. In the previous version, "target shape" and "formation area" were intended to refer to the same concept, but the inconsistent wording caused confusion. In the revised manuscript, we have unified the terminology and consistently use "target shape" throughout **line 400-405:**

As shown in Fig. 7, the shape-assembly velocity that drives the swarm to form the desired shape consists of five parts:

$$v_i^{shape} = v_i^{ent} + v_i^{avd} + v_i^{re} + v_i^{rb} + v_i^{dis}$$

The first two terms mainly guide robots into the target shape without collisions, while the last three terms regulate how the swarm is distributed inside the shape. The entry command is designed to guide the swarm toward the target shape.

- 459: How is the global velocity of the formation estimated? And is the estimation method distributed?

We thank the reviewer for this question. The "global" formation velocity in our framework is not estimated from observations; it is a commanded collective migration velocity. Specifically, we

introduce a migration term v_i^{mig} that is identical for all robots and is added to each robot's control velocity so the swarm translates with a common desired speed and direction. At the same time, the center of the target shape is advanced using the same migration velocity, so robots track a moving target shape consistently during navigation. The migration velocity can be (i) predefined by the user or mission script, or (ii) generated online as a swarm-level command inferred by our distributed LLM module. We clarify this point in the revised manuscript **line 480–482:**

To enable the swarm to move as a whole toward a common destination, we introduce a collective migration term v_i^{mig} that is identical for all robots. This velocity drives the whole swarm to move with the same desired speed and direction.

- 460: Why "during group flight" when this section is about a robot outside the formation area entering the formation area?

We thank the reviewer for pointing this out. This was a placement mistake in the previous version, where the flight (migration) velocity description was incorrectly included in the shape-entry part. We have move relevant text to navigation part.

- 463: What is described in this section is not a k-means algorithm. Why are the authors calling this k-means exploration?

We thank the reviewer for this comment. We clarify that the exploration directive described in this section is based on each robot identifying its assigned region from nearby target points and moving toward the region center. This mechanism does not involve K-means clustering, and we have removed all references to K-means from the revised manuscript to avoid confusion.

- 464: Why "further"? Is there spreading occurring during the entry phase?

We thank the reviewer for pointing this out. The word "further" in the previous wording was misleading, as it implied a sequential process (enter first, then spread). In our method, the entry behavior and the exploration/spreading behavior occur simultaneously as part of the same control update. We have revised the sentence to reflect this concurrent process in **line 413–414:**

To better drive the swarm to spread within the target shape, we design a region exploration velocity v_i^{re} that makes each robot continuously move toward the center of its assigned region

- 465: Notation of v_{kms} does not match eq. 8

We thank the reviewer for pointing this out. This was a notation inconsistency in the previous version. In the revised manuscript, we rename v^{kms} to the region-exploration term v^{re} and correct Eq.6 (previous Eq,8) to consistently use v_i^{re} in **line 416-418:**

The region exploration velocity is defined as:

$$v_i^{re} = \mu_2 \left(\frac{\sum_{j \in s_i^{safe}} w_{i,j} \cdot \mathrm{tar}_j}{\sum_{j \in s_i^{safe}} w_{i,j}} - p_i \right) \tag{6}$$

- 470: The "assigned region" value is called "ownership region" in the previous section

We thank the reviewer for pointing this out. In the revised manuscript, we have unified the terminology and consistently use **associate region** to refer to this concept.

- 472-474: This seems to only be true under certain density conditions (density of robots and density of target points). This should be explained/justified.

We thank the reviewer for this comment. The statement is not meant to rely on any particular robot/target-point density condition. Our intent is a control-design clarification about how we compute the exploration direction.

In the exploration term, robot i forms a centroid-like attraction direction from target points in its associate region. If some of those points lie inside (or very close to) a neighbor's safety/repulsion zone(within r_{avoid}), then including them would create an attraction component that pulls robot i toward a location where the repulsive velocity may push it away. This attraction–repulsion conflict can cause inefficient "back-and-forth" motion and unnecessary activation of the repulsive term, regardless of global densities. By restricting the centroid computation to the safe subset s_i^{safe}, we simply exclude target points that are too close to neighbors, so the exploration term no longer contains a component that drives i toward neighbors' repulsion regions, which improves motion efficiency and reduces needless repulsive responses. We have revised the manuscript to reflect this more precisely in **line 424-426:**

Moreover, restricting the computation to the safe subset s_i^{safe} keeps robot i from being attracted to target points that are too close to its neighbors, which reduces unnecessary activation of the repulsive velocity.

- 475: This title is an ungrammatical phrase

We thank the reviewer for pointing this out. In the revised manuscript, we removed this ungrammatical subsection title and reorganized the content under the "Form the target shape" section.

- 477: Clusters of what? Clusters have not been referred to in the Methods section before now.

We thank the reviewer for pointing this out. In the previous version, "clusters" was an imprecise analogy and was not properly defined. In the revised manuscript, we removed this wording and instead describe the effect directly, noting that the region-exploration term alone leads to a relatively loose distribution inside the target shape in **line 427-428:**

However, the region exploration command alone only yields a relatively loose distribution of robots inside the target shape, as illustrated in Fig. 1a.

- 478: The term v_{kms} does not diffuse. What is meant here?

We thank the reviewer for pointing this out. The phrase "v_{kms} cannot diffuse" in the previous version was inaccurate and misleading (we did not mean a diffusion process in a graph-theoretic sense). In the revised manuscript, we removed this wording and clarified the intended meaning: the region-exploration term alone tends to produce a relatively loose, non-uniform distribution inside the target shape, so an additional region-balancing mechanism is introduced to enforce uniformity, consistent with the bubble-raft analogy.

- 480: "assigned region" is called "ownership region" above

We thank the reviewer for pointing this out. In the revised manuscript, we unified the terminology and consistently use associate region throughout

- 482: the absolute value notation is not used in eq. 10

We thank the reviewer for this comment. We have corrected the notation in Eq.8 (previous Eq.10) to ensure consistency with the text and removed the absolute value symbol where it was incorrectly used.

- 487-490: This seems like it would only be true under very specific conditions of adjacent regions. Needs to be better explained/justified

We thank the reviewer for this comment. We agree that the relation in the previous draft was written too strongly. The effect we intended to model is a first-order (small-displacement) approximation of how the shared interface between two adjacent associate regions shifts when robot i moves. In general, the region-size change is dominated by the motion component along the interface normal, while tangential motion has negligible first-order effect. Therefore, we revised the manuscript to explicitly state this is a local linearization and we replaced the equality with an approximation using the unit direction $\bar{p}_{ij}$. We have revised the paper to make this more clearly in **line 438-442:**

It can be observed that when robot i moves, the change of the size of neighbor j's associate region is mainly induced by the motion component along the shared boundary normal. Therefore, the variation of j's region size can be approximated as $\frac{\partial S_j}{\partial p_i} \approx -\frac{L_{ij}}{2}\bar{p}_{ij}$, where L_{ij} is the length of the shared edge and $\bar{p}_{ij} = (p_j - p_i)/\| p_j - p_i \|$. This is a local first-order approximation and we ignore higher-order geometric effects for simplicity.

- 491: Their combined size will not always remain constant when the robots are moving (i.e., the region of the neighbor of robot i can change along its adjacency to a different one of its neighbors, not only along its adjacency to i). This statement about remaining constant needs more explanation/justification.

We thank the reviewer for this comment and agree that the statement in the previous version was ambiguous if interpreted along the full motion of the swarm. In Eq.10 (previous Eq.12), the equality is not a claim that the combined region size remains constant over time while all robots move. Rather, it is a local relation for the partial derivative with respect to p_i. When evaluating $\partial(\cdot)/\partial p_i$, we consider an infinitesimal displacement of robot i while keeping other robots fixed, so this perturbation only transfers boundary target points between S_i and its adjacent regions S_j. Under this local perturbation, the total $S_i + \sum_{j \in N_i} S_j$ is invariant with respect to p_i, which yields Eq. (10). We have revised the manuscript to clarify this point explicitly in **line 442–446:**

When evaluating the partial derivatives with respect to p_i, we only account for the effect of an infinitesimal displacement of robot i. This displacement redistributes boundary target points between S_i and the neighboring regions S_j, the total size $S_i + \sum_{j \in N_i} S_j$ remains constant with respect to p_i, we have:

$$\frac{\partial S_i}{\partial p_i} + \sum_{j \in N_i} \frac{\partial S_j}{\partial p_i} = 0. \tag{10}$$

- 506: why sequential?

We thank the reviewer for pointing this out. The word "sequential" in the previous version was an inaccurate and confusing description, since our method does not perform any sequential diffusion process. The region-balancing term acts simultaneously on each robot through local push–pull interactions with its neighboring regions. In the revised manuscript, we removed "sequential diffusion", and revised the description to state that this interaction drives robots away from locally dense areas and toward sparse region , thereby improving uniformity over the target shape. We clarify this point in the revised manuscript **line 458–460:**

This push–pull interaction between neighboring regions drives robots away from locally dense areas and toward sparsely populated ones, thereby improving the uniformity of the swarm distribution over the target shape.

- 508: Is this formation graph different from the graph G stored at initialization? Are the robots then not trying to occupy a position in graph G? Very confusing; needs better explanation.

We thank the reviewer for pointing this out. Here "formation graph" refers to the target shape representation G defined at initialization, not a different graph. We have removed the ambiguous word "formation graph" and consistently use "target shape G" in the revised manuscript, **line 461-463:**

In the bubble-raft analogy, the forces that drive the bubbles to form a uniform pattern arise not only from the bubble sizes but also from the distances between bubbles, eventually making them tightly packed with equal spacing. Inspired by this mechanism, we design a second objective for uniform distribution:

- 512: Should already mention that the global average distance is estimated using a consensus mechanism

We thank the reviewer for pointing this out. In the revised manuscript, we mention earlier that the global average inter-robot distance is estimated via a consensus mechanism, and we revised the text accordingly **line 464–470:**

$$\mathcal{H}_2(p_i) = \sum_{j \in N_i} (l_{ij} - \bar{l})^2$$

where $\bar{l} = \sum_{i \in U} \sum_{j \in N_i} l_{ij} / \sum_{i \in U} \mathrm{card}(N_i)$ denotes the average distance. Since the global average distance $\bar{l}$ is not directly available to each robot, we adopt a consensus mechanism. Each robot computes the average distance to all of its neighbors and exchanges this quantity with them. The updated estimate of the average distance for robot i is given by:

$$\bar{l} = \frac{\bar{l}_i + \sum_{j \in N_i} \bar{l}_j}{\mathrm{card}(N_i) + 1}$$

where $\bar{l}_i = \sum_{j \in N_i} l_{ij} / card(N_i)$ denotes the local average distance of robot i.

- 516: This does not seem to be a correct use of "diffusion", as a technical term

We thank the reviewer for pointing this out. We agree that "diffusion" was an incorrect technical term in this context. In the revised manuscript, we removed this expression.

- 533: How are the next positions predicted?

We thank the reviewer for this question. In the revised manuscript, since the control time step is very short (0.05 s), we no longer predict neighbors' next positions and instead use their current positions directly in the collision-avoidance computation. In the previous version, the next position was predicted by a one-step forward Euler estimate, i.e., current position plus velocity multiplied by the time step. We have clarified this in the revised manuscript **line 490–491:**

Let B denote the obstacle set, which is constructed from the LiDAR point cloud together with the positions of other robots within the sensing range.

- 535: An intuitive textual description of the condition should be given to the reader, in addition to the equation.

We thank the reviewer for this suggestion. In the revised manuscript, we add an intuitive explanation of Eq.17 (previous Eq.19). Specifically, the inequality checks whether moving along direction $\vec{v}$would pass sufficiently far from obstacle ob_j. Since $\| \vec{v} \times \vec{u}_i \|$corresponds to the sine of the angle between the motion direction and the line-of-sight to the obstacle, the condition ensures that the closest distance from the straight-line path defined by $\vec{v}$ to $ob_j$is no smaller than r_{avoid}. Directions that would bring the robot closer than $r_{\text{avoid}}$are therefore marked as inadmissible. We have revised the text accordingly in **line 493-496:**

$$\|\vec{v} \times \vec{u}_i\| \geq \frac{r_{avoid}}{d_i} \tag{17}$$

where $\vec{u}_i = \frac{p_i - ob_j}{\|p_i - ob_j\|}$, $d_i = \|p_i - p_{obj}\|$, and $ob_j \in B$. This constraint guarantees that the minimal distance between the straight-line path defined by $\vec{v}$ and obstacle ob_j is at least r_{avoid}, directions that would lead to a closer approach are marked as inadmissible.

- 542: The term "shape assembly velocity" has not been introduced

We thank the reviewer for pointing this out. In the revised manuscript, we explicitly introduce and define the term "shape-assembly velocity" before it is used, and we have revised the text accordingly in **line 400–402:**

As shown in Fig. 7, the shape-assembly velocity that drives the swarm to form the desired shape consists of five parts:

$$v_i^{shape} = v_i^{ent} + v_i^{avd} + v_i^{re} + v_i^{rb} + v_i^{dis} \tag{3}$$

- 547-548: Avoid calling a metric a "rate" when there is no unit of time incorporated. It is confusing for the reader. If you were calculating the proportion of robots or the proportion of target points per a unit of time, it would be okay. But since there is no unit of time, it is confusing. These two names should be changed to better reflect the metrics.

We thank the reviewer for pointing this out. We agree that "rate" was confusing without an explicit time unit. In the revised manuscript, we renamed these metrics to **Entry Percentage and Coverage**

Percentage to reflect that they are proportions rather than time-based rates.

- 551: Predefined as/according to what? Some details should be given.

We thank the reviewer for pointing this out. In the revised manuscript, we remove the vague "predefined threshold" and explicitly set the entry threshold to r_{avoid}. This choice is made for consistency, since r_{avoid} is the same safety clearance used in our obstacle-avoidance module and thus provides a physically meaningful criterion for whether a robot has entered the target shape. We have revised the text accordingly in **line 531–533:**

$$Entry\ Percentage = \frac{n_{enter}}{n_{robots}} \tag{22}$$

where $n_{enter} = \text{card}(\{i \mid tar_G^i < r_{avoid}\})$, we use r_{avoid} as the entry threshold for consistency with the avoidance clearance.

- 557: What are the regions? Does this mean the ownership regions? Inconsistent terminology.

We thank the reviewer for pointing this out. Here "regions" refers to the robots' associate regions. We have unified the terminology and clarified this in the revised manuscript **line 538-539:**

Uniformity of the swarm is quantified using the coefficient of variation (CV) of the robots' associate region sizes and the average neighbor distances, defined as:

Comment about data and code:

The data needs to be publicly available in a permanent database, such as Zenodo or Dryad. Making the code available on GitHub is great. However, since GitHub entries are not necessarily permanent, the code should additionally be made publicly available in a permanent database, such as Zenodo.

We thank the reviewer for this suggestion. We agree that long-term accessibility is important. In addition to releasing the code on GitHub, we archive the code in Zenodo, and cite the corresponding DOI in the revised manuscript(**10.5281/zenodo.17921957**).

All modifications in the revised manuscript are highlighted in red for clarity and ease of review.

Reviewer #2 (Remarks to the Author):

Comment: The authors have addressed the comments raised in the previous round of review and conducted satisfactory revisions to the methodology and the additional experiments. However, there are still some issues that need to be resolved.

Answer: We thank the reviewer for the careful reading of our revised manuscript and for the positive assessment of the improvements made. We also appreciate the additional comments provided in this round and address them in detail below.

Comment 1: The description of the tensor-parallel partitioning in the MLP block appears to contain an inconsistency. The text states that W_{down} is partitioned column-wise and the results are concatenated. However, in standard tensor parallelism (e.g., Megatron-LM), the second linear layer (W_{down}) is typically partitioned row-wise, and the results are summed (all-reduce), not concatenated, because the input h^p is already split across the feature dimension. Let's say if h^p is a partial vector (e.g., size 100) and is $W_{down}^{(p)}$ a column-split matrix (e.g., size Total × 100), the dimensions don't align for a standard dot product representing the full transformation. If W_{down} is indeed column-split, please clarify how the matrix multiplication dimensions align given that h^p is a local shard.

Answer: We appreciate the reviewer's careful reading and for highlighting this inconsistency. The reviewer is correct that the MLP tensor-parallel description in the manuscript contained an error. The implementation follows the standard tensor-parallel scheme used in Megatron-LM, and this scheme is adapted to the distributed robot-swarm setting in our work.

Concretely, in the MLP, W_{up}, W_{gate} are partitioned column-wise, yielding local shards $u^{(p)}, g^{(p)}$. and therefore $h^{(p)} = \mathrm{SiLU}(g^{(p)}) \odot u^{(p)}$ is also sharded along the feature dimension. Accordingly, W_{down} is **partitioned row-wise**, i.e., each group stores $W_{\mathrm{down}}^{(p)} \in \mathbb{R}^{(d_{\mathrm{ff}}/P)\times d}$, so that the local multiplication $M^{(p)} = h^{(p)} W_{\mathrm{down}}^{(p)}$ is dimensionally consistent and produces a full-sized contribution in $\mathbb{R}^{N\times d}$. The final MLP output is obtained via a **sum-reduction across groups** (implemented as broadcast + summation), rather than concatenation:

$$M = \sum_{p=1} M^{(p)}$$

This description has been corrected in the revised manuscript in **Supplementary Material Section 6**.

The MLP process operates on the full attention output O. It uses three weight matrices $W_{\text{up}}, W_{\text{gate}}, W_{\text{down}}$, where W_{up} and W_{gate} are partitioned column-wise so that group p stores $W_{\text{up}}^{(p)}$ and $W_{\text{gate}}^{(p)}$, while W_{down} is partitioned row-wise so that group p stores $W_{\text{down}}^{(p)}$. Robots in group p compute:

$$u^{(p)} = OW_{\text{up}}^{(p)}, g^{(p)} = OW_{\text{gate}}^{(p)},$$
$$h^{(p)} = \text{SiLU}\left(g^{(p)}\right) \odot u^{(p)}, M^{(p)} = h^{(p)} W_{\text{down}}^{(p)}, \tag{21}$$

where $M^{(p)}$ is the shard-wise MLP output. As in standard tensor parallelism[15], all robots perform a sum-reduction (broadcast + summation) over their local $M^{(p)}$to obtain the full MLP output $M = \sum_{p=1}^{P} M^{(p)}$, which is then used as the input X of the next Transformer block.

Comment 2: The manuscript suffers from numerous grammatical errors and awkward phrasing (e.g., 'compare with' instead of 'compared with', 'target not arrive', missing articles). The authors must perform a thorough proofread to correct these language issues before final publication.

Answer: We thank the reviewer for the helpful suggestion. We have carefully proofread the manuscript and corrected the grammatical errors and awkward phrasing throughout. All revisions are highlighted in red in the revised version.

Comment 3: Several acronyms are used throughout the manuscript without being defined upon their first appearance. For example, while terms like "MLP" are widely recognized in the field, standard academic practice requires that they be formally defined when first introduced. Please carefully scan the text to ensure all abbreviations are spelled out initially to improve accessibility for a broader audience.

Answer: We thank the reviewer for the suggestion. We have revised the manuscript so that all acronyms are defined upon first use. Concretely, we added first-use definitions for: MPI (Message Passing Interface), RMSNorm (Root Mean Square Layer Normalization), RoPE (Rotary Position Embeddings), SiLU (sigmoid linear unit), and KD-tree (k-dimensional tree). All revisions are highlighted in red in the revised version.

Comment 4: Caption of Fig 2: "Formation for a cartoon mouse shape using 500 robots under our method, Mean-shift, and Image Moment.(More comparison can be found in Supplementary Video 4)". It seems like you forgot to add the Voronoi Partition.

Answer: We thank the reviewer for pointing out this omission. The Voronoi Partition baseline has now been added to the caption of Fig. 2 in the revised manuscript.

Reviewer #3 (Remarks to the Author):

Comment: I provided a quite long list of comments in the previous round, and the authors have been very thorough in addressing all of my comments. I would like to commend the authors on the quality of their revision. All of my comments have been addressed and I am satisfied with the revision.

Answer: We sincerely thank the reviewer for the time and effort invested in providing detailed and constructive comments throughout the review process. The previous feedback has been highly valuable in improving the clarity, quality, and overall presentation of the manuscript. We appreciate the reviewer's positive assessment of the revision.

Comment 1: The text added at line 202 is nonsensical and needs revision.

Answer: We thank the reviewer for pointing this out. We have revised the text at line 202 to clarify the intended meaning. In particular, we replaced the ambiguous phrasing with a clearer description. Line 188-191:

These frequent exchanges cause large amounts of ineffective motion, and some robots are assigned to opposing target points, which induces conflicting motion directions and repeated collision-avoidance maneuvers, resulting in slow convergence and occasional failure to reach the assigned targets.

Comment 2: For the code and data repository: At the DOI the authors have provided, the code is available (this is good), but not the data. All experimental data should also be made publicly available in a permanent database (such as Zenodo or Dryad).

Answer: We thank the reviewer for the suggestion. We have now added the experimental data to the repository and made it publicly available in a permanent archive at the provided DOI.

Comment 3: There are some minor typos throughout the paper. Please conduct a thorough proofread.

Answer: We thank the reviewer for pointing this out. We have conducted a thorough proofreading of the manuscript and corrected the minor typos throughout. All changes are highlighted in red in the revised version.

Comment 4: In all of the figures, most of the text is far too small. Please increase the font sizes in the figures so that the text can be read easily.

Answer: We thank the reviewer for the suggestion. We have increased the font sizes across all figures to improve readability.

Comment 5: At lines 300-305, several times you have written "large model" when you meant to write "large-language model".

Answer: We thank the reviewer for pointing this out. The relevant content has been moved to the Supplementary Material, and we have revised the wording throughout that section to use "large language model" consistently instead of "large model."

Comment 6: The sections on the application of LLMs are much improved in terms of technical soundness. However, they are very tangential to the main topic of the paper, and they do not report any results that are relevant to understanding the performance of the proposed "bubble-raft" algorithm. Therefore, I recommend moving these sections to the supplementary materials.

Answer: We thank the reviewer for this insightful comment and agree that the LLM-related sections are tangential to the main focus of the paper and do not directly contribute to evaluating the performance of the proposed bubble-raft algorithm. This part is intended primarily as an application of our framework to distributed robot-swarm inference, and the underlying techniques largely follow standard tensor-parallel deployment practices (Megatron-LM[1]) with engineering-oriented implementation rather than methodological novelty. Given the limited relevance to the bubble-raft results, we have moved these sections to the supplementary materials. We also view this direction as a promising topic for future exploration.

[1] Shoeybi, M., Patwary, M., Puri, R., LeGresley, P., Casper, J., & Catanzaro, B. Megatron-LM: Training Multi-Billion Parameter Language Models Using Model Parallelism. arXiv:1909. 08053 (2020).
