# Supplementary material for: Bubble-raft inspired shape-assembly in flying robot swarm for uniform formation and obstacle traversal
Source: Commun Eng. 2026 Jul 2;5:178. doi: 10.1038/s44172-026-00715-3 (PMC13696712; doi:10.1038/s44172-026-00715-3)
Supplement: Supplementary file 2 [file 44172_2026_715_MOESM2_ESM.pdf]

Supplementary Information for

Bubble-raft Inspired Shape-assembly in Flying Robot Swarm for Uniform Formation and Obstacle Traversal

The PDF files includes:

Supplementary Methods

Supplementary Figures 1 to 29

Supplementary References

Other supplementary material for this manuscript includes the following:

Supplementary Movie 1: Complex shape-assembly for 3D shapes—Transformation from spherical to DNA-shaped formation

Supplementary Movie 2: Formation transition between letters "SWARM"

Supplementary Movie 3: Shape-recovery in dynamic swarm size

Supplementary Movie 4: Comparison with three state-of-the-art methods

Supplementary Movie 5: Flying through unknown dense environment using lidar in simulation

Supplementary Movie 6: Obstacles Avoidance in dense environment in real world

Supplementary Movie 7: Autonomous search and rescue via LLM-Guided swarm shape assembly

1 Additional Information for Bubble-raft Strategy and Related Works

1.1 The Bubble Raft Inspired Shape Assembly Strategy

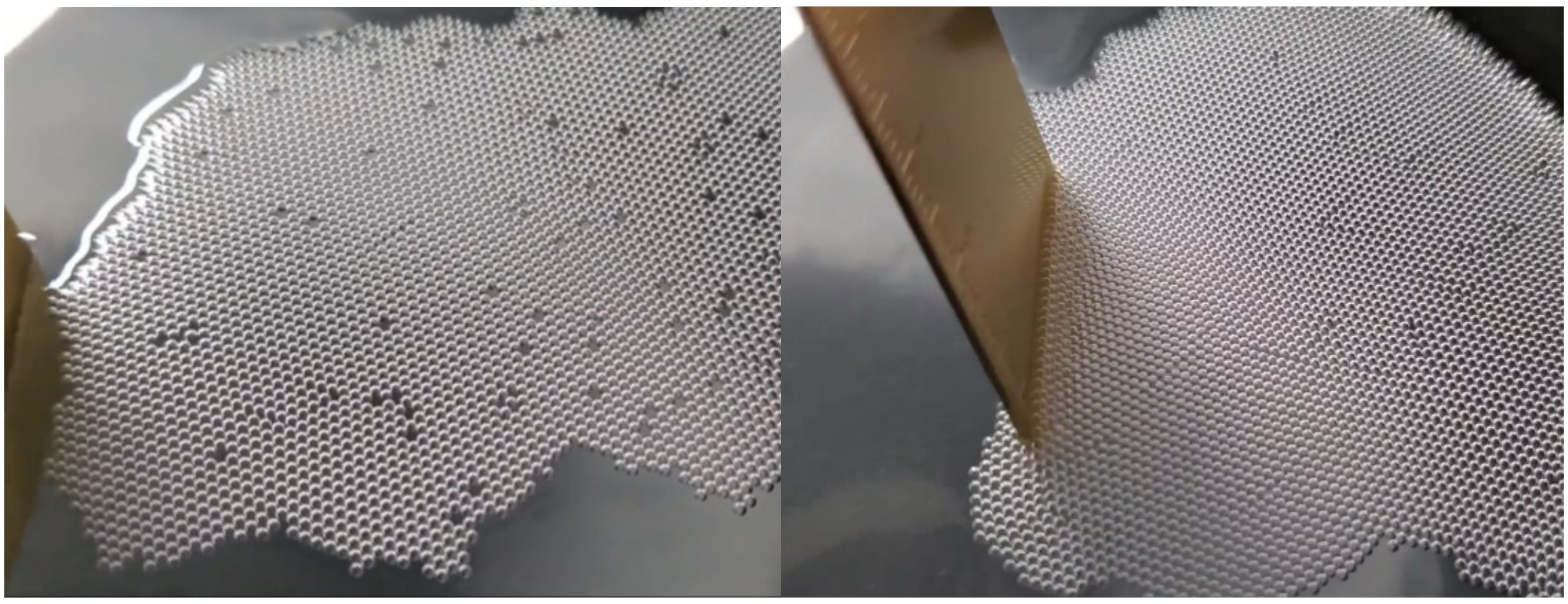

Fig 1 | Bubble raft under mechanical stress, showing size redistribution and positional rearrangement of bubbles due to interfacial and capillary forces.

A two-dimensional bubble raft is a planar array formed by soap bubbles that spontaneously aggregate on the surface of a soap solution, serving as a model for atomic arrangements in solids. Historically, Bragg and others[1] discovered that when a large number of bubbles are blown onto the surface of a solution containing glycerin and surfactants, they spontaneously organize into structures resembling the close-packed crystal planes of metals. In our experiment, we pour soap solution into a shallow dish and continuously blow air onto the surface using a straw or fine needle, causing similarly sized bubbles to accumulate and form a raft. Side lighting is used during the experiment, and the arrangement of the bubble raft is recorded from above using a camera or microscope. When a sufficient number of bubbles are present, the raft forms a macroscopically visible crystalline structure (see Fig. 1), in which defects and grain boundaries are also commonly observed.

In a bubble raft, each bubble is enclosed by a thin liquid film and is subject to surface tension. The surface tension γ tends to minimize the interfacial area of the bubble. For a single spherical bubble, the pressure difference between the inside (P_{in}) and the outside (P_{out}) satisfies the Young–Laplace equation[19]

$$P_{\text{in}} - P_{\text{out}} = \gamma\left(\frac{1}{R_1} + \frac{1}{R_2}\right) \tag{1}$$

For circular bubbles floating on the water surface, the equation simplifies to $P_{\text{in}} - P_{\text{out}} = \frac{2\gamma}{R}$. This implies that smaller bubbles have higher internal pressure, and when bubbles of different sizes are in contact, the shared film curves toward the smaller one to achieve Laplace balance[20].

More importantly for our purpose, **bubbles exhibit effective inter-bubble forces** that lead to near-uniform spacing[21]. These forces consist of

- Short-range repulsion when bubbles are very close (due to compressed liquid films and pressure difference), and
- Longer-range interactions mediated by surface deformations (capillary effects), which may be attractive or repulsive depending on the configuration.

When two soap bubbles of equal size come into contact, they experience an attractive potential due to deformation of the fluid surface, given by:

$$U_{\text{attr}}(\rho) = -\pi R^4 \rho_{\text{solution}} g \left(\frac{\beta}{\alpha}\right)^2 A K_0[\alpha\rho] \quad (2)$$

Here, K_0 is the modified Bessel function of the second kind, and $\rho = r/R$ is the dimensionless distance between bubble centers.

At very short distances, the bubbles begin to overlap, leading to a repulsive potential due to elastic deformation of the interfaces:

$$U_{\text{rep}}(\rho) = \pi R^4 \rho_{\text{solution}} g \frac{(2-\rho)^2}{\alpha^2} \text{for } \rho \leq 2 \quad (3)$$

The full bubble potential combines these two contributions[25]:

$$U(\rho) = -\pi R^4 \rho_{\text{solution}} g \left(\frac{\beta}{\alpha}\right)^2 A K_0(\alpha\rho) + \begin{cases} \pi R^4 \rho_{\text{solution}} g \dfrac{(2-\rho)^2}{\alpha^2}, & \rho \leq 2 \\ 0, & \rho > 2 \end{cases} \quad (4)$$

where

R : bubble radius

ρ_{solution} : density of the solution

g : gravitational acceleration

$\alpha = R/a$: ratio of bubble radius to Laplace constant

$\beta = b/R$: dimensionless contact ring radius

A : constant determined by boundary conditions

K_0 : zeroth-order modified Bessel function of the second kind

$\rho = r/R$: normalized distance between bubble centers

This potential has a well-defined minimum at a finite separation $\rho = \rho_0$, representing the equilibrium distance between neighboring bubbles. At shorter separations, repulsion dominates due to geometric constraints; at larger separations, the attraction decays exponentially. Although this interaction potential does not enforce uniform distances explicitly, it naturally leads to a stable, approximately hexagonal packing when applied to many bubbles[22]. The minimization of the total potential energy under confinement and crowding yields uniform spacing, much like atoms in a crystalline lattice[23]. This emergent order reflects the balance of attractive and repulsive components intrinsic to bubble interactions.

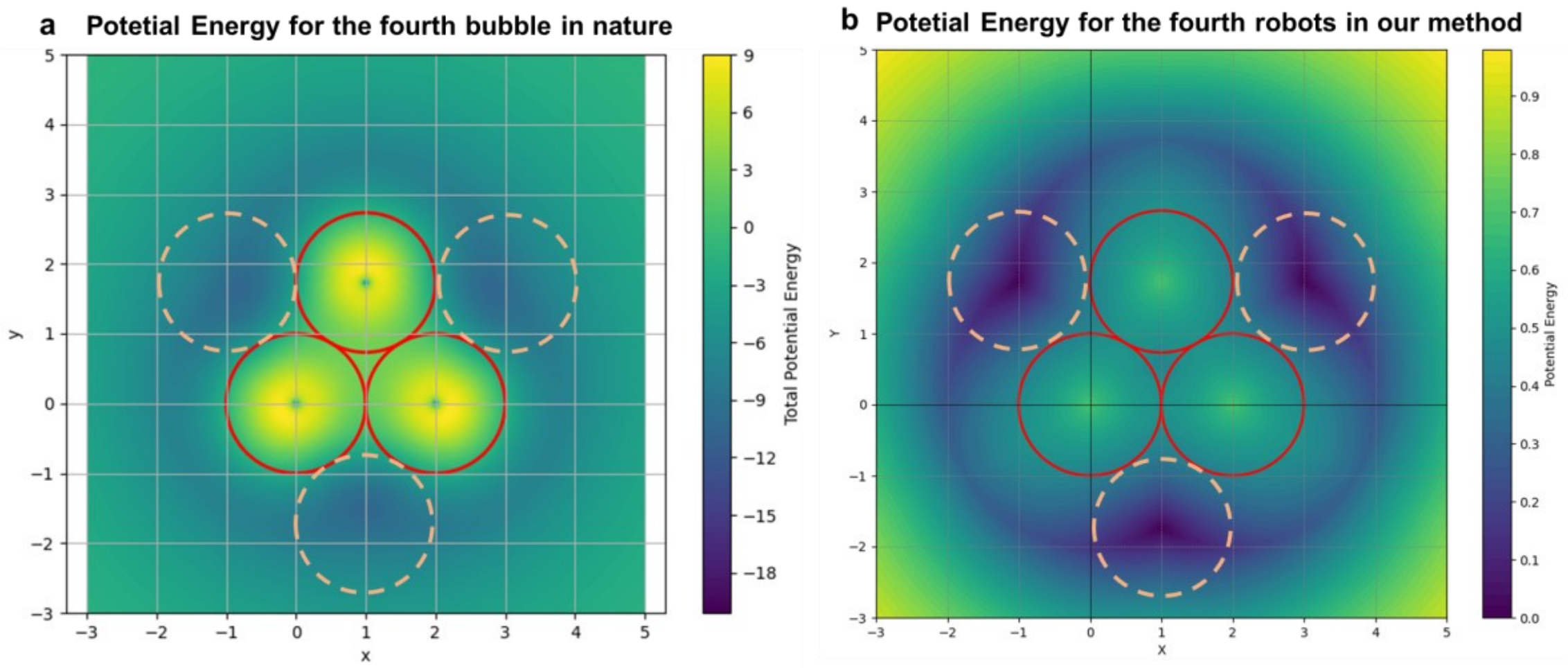

Fig 2 | Comparison between the physical potential energy landscape of a bubble raft and the potential field in our algorithm. Red circles represent the initial tightly packed three-bubble configuration, while orange dashed circles indicate positions where the total potential is minimized. **(a)** is computed using the physical model described in Eq. (4), representing the classical potential energy distribution of interacting bubbles. **(b)** shows the potential field derived from the loss function proposed in this paper. The two potential fields exhibit remarkably similar spatial structures, both forming well-defined minima around regular, close-packed configurations. Both systems favor such arrangements by minimizing a composite interaction energy—mechanical in bubbles, algorithmic in swarms—highlighting how simple local rules can give rise to globally ordered structures.

In essence, the proposed algorithm can be viewed as a discrete optimization process that minimizes a global loss function composed of multiple interaction objectives—namely, uniform spacing, balanced region size, and centroidal assignment. In regional balance, the associate region of the robot can be approximately equivalent to the size of a bubble, the size adjustment between bubbles and the size of the associate region is actually equivalent. This mirrors the total energy minimization process observed in bubble raft systems. As illustrated in Figure 2, the potential energy landscape generated by the interaction of three bubbles reveals distinct energy minima at positions that favor regular, close-packed configurations. These minima arise from the superposition of short-range repulsion and long-range attraction—physical mechanisms that are directly analogous to the artificial control potentials designed in our method.

It should be noted that the bubble potential in Equation (4) is merely for physical intuition and design inspiration. We did not directly substitute the solution density from the physical model into the global optimization in this paper. In bubble model, the force magnitude may be under different density but the process of generating raft remain unchanged. In our control, "local density" is implicitly represented by the size of the associated area of each robot (i.e., the number of target points within that area): the larger the associated area, the lower the local robot density, and vice versa. The control term achieves the redistribution from high density to low density by balancing the area size and the

neighbor distance, and thus does not require explicit calculation or input of fluid density.

This framework allows us to simulate and reproduce energy-minimizing patterns seen in nature—such as close-packed arrangements and spatially uniform distributions—across a wide range of arbitrary target shapes. It provides a principled way to explore how local interactions give rise to global organization, extending the physical intuition of bubble rafts to programmable, shape-aware swarm assemblies in both two and three dimensions.

1.2 Related Works

Table 1 Related works of swarm formation obstacle traversal

Paper	method	specific formation	large scale	uniformity	obstacle traversal	self-healing
[1-2]	mean-shift	√	√	×	×	√
[3]	mean-shift	√	√	√	×	×
[4-5]	flocking	×	√	×	√	√
[6]	flocking	√	√	×	×	√
[7-9]	Local swapping	√	√	√	×	×
[10]	Image moment	√	√	×	×	√
[11]	NMPC	×	×	×	√	×
[12]	reaction-diffusion	√	√	×	×	√
[13]	artificial light field	√	√	√	×	×
[14]	Voronoi	√	×	×	√	×
[15]	Voronoi	√	×	×	√	√
[16]	Trajectory-planning	√	×	√	√	×
[17]	Trajectory-planning	√	√	√	×	×
**Ours**	**bubble-raft**	√	√	√	√	√

Mean-shift–based methods treat the desired shape as a density field and let robots repeatedly give up their current locations and move toward nearby regions of higher "shape density," avoiding explicit target assignment. Sun et al. [1] first used this idea for large-scale shape assembly of ground swarms, combining a distance-field representation of the target pattern with distributed mean-shift exploration and simple local interactions. This enables assignment-free formation of highly non-convex 2-D shapes and basic self-healing, but uniformity and safety are mainly handled implicitly through repulsion. Li et al. [2] extend this framework to handle changes in swarm size, so that the same pattern can be maintained as robots join or leave. Zhang et al. [3] further refine the control law by improving how local density is estimated, which reduces conflicts between robots and significantly speeds up convergence compared with assignment-based baselines. Overall, mean-shift methods achieve complex planar formations at large scale without target assignment, but they are not designed for precise uniform filling or guaranteed collision-free traversal in cluttered 3-D environments.

Flocking-based methods draw on local attraction, alignment and repulsion rules so that each robot reacts only to nearby neighbors yet the group moves as a cohesive swarm. Vásárhelyi et al. [4] tune such local rules through optimization and demonstrate large groups of quadrotors flying in confined

spaces and around static obstacles, achieving impressive scalability and robustness but only loose flock-like formations rather than precise shapes. Cheng and Nagpal [5] add mechanisms for robustness and self-repair, so that a lattice-like formation can close gaps when agents fail, again using local flocking interactions rather than explicit shape specification. Yu et al. [6] introduce a GRF-based predictive flocking controller that can track time-varying patterns and negotiate obstacles by shaping the desired velocity field, with validation in corridor-like environments. Overall, flocking controllers are well suited for large cohesive swarms and offer some robustness and obstacle negotiation, but they typically yield diffuse crowds instead of task-specific, uniformly filled formations and do not provide strong guarantees for dense swarms in cluttered environments.

Local-swapping methods address shape formation by letting robots iteratively exchange goal locations with nearby teammates instead of solving a global assignment once and for all. Wang and Rubenstein [8] propose a local task swapping algorithm in which each robot first picks a nearby target inside the desired shape and then repeatedly negotiates swaps with neighbors whenever exchanging goals shortens the total path length and reduces potential collisions. This yields efficient, collision-free motions and has been demonstrated with up to 1024 simulated robots and 100 physical robots. Zhang and Piao [7] extend this idea with vein-based coalitions, organizing target locations into vein-like groups and coordinating robots within and across these groups to improve scalability and path quality. Chu et al. [9] further study massive shape formation on grids, combining local coalition mechanisms with structured fields to support very large populations. Overall, local-swapping approaches can realize precise target-assignment–based formations at large scale, but the reliance on discrete local swaps makes it difficult to design smooth obstacle-avoidance controllers that preserve formation during flight.

Voronoi-based methods use geometric partitions to achieve spatial distribution of robots. Breitenmoser et al. [14] study Voronoi coverage in non-convex environments, where each robot maintains its Voronoi cell and moves toward the cell centroid, yielding a centroidal Voronoi tessellation that achieves coverage of target shape while a local path planner handles non-convex obstacles. Cao et al. [15] build on centroidal Voronoi tessellations for multi-robot formation control, combining a CVT-based controller with a “health optimization” mechanism so that robots maintain a desired formation while balancing workload and robot condition. These approaches provide elegant geometric tools for coverage and 2-D formation, but they are not designed for dense 3-D or 2-D swarms that must both uniformly fill arbitrary shapes and move safely through dense obstacle environments.

2 Convergence Analysis

2.1 Theoretical Analysis

We provide a Lyapunov-based[24] convergence analysis of the proposed bubble-raft-inspired

swarm formation algorithm. The goal is to rigorously demonstrate that, under the designed distributed control laws, the system will converge to a stable configuration with uniform robot distribution.

Let the swarm consist of N robots with positions $x_i \in R^3$ for $i = 1, \dots, N$. Each robot adjusts its velocity based on the following control law:

$$\dot{x}_i = v_i = v_i^{\text{re}} + v_i^{\text{rb}} + v_i^{\text{dis}} \tag{5}$$

Each term in the velocity update represents a gradient descent behavior on a corresponding energy function:

- v_i^{re}: moves robot toward the centroid of its assigned region.
- v_i^{rb}: reduces area difference between neighboring regions.
- v_i^{dis}: equalizes distance between neighboring robots.

Remark on $\boldsymbol{v_i^{\text{re}}}$: The term v_i^{re} is derived from the optimization objective of complete shape coverage using a Voronoi-based formulation. The computational complexity of this step is as follow:

$$O\left(\log M + \frac{M}{V} \cdot r_{\text{sense}}^3\right)$$

where M is the total number of target points, V is the volume of the target shape, r_{sense} is the sensing range, M/V is the average density of target points. In practice, we use a KD-tree to efficiently query target points within the sensing range, reducing the overhead from linear scan to logarithmic in M plus the number of local points.

In particular, this region exploration term is equivalent to minimizing the Centroidal Voronoi loss function over the set of target points $\mathcal{T} = t_1, \dots, t_M$:

$$J = \sum_{i=1}^{N} \sum_{t_j \in s_i} |t_j - x_i|^2 \tag{6}$$

where s_i is the subset of target points assigned to robot i via Voronoi or K-means partitioning. This objective aims to minimize the total squared distance between robots and their assigned points, thereby encouraging each robot to act as a centroid.

This formulation is consistent with the goal of the well-known **Floyd centroidal Voronoi tessellation (CVT)**[25] objective in the continuous domain. In our discrete setup, the loss function becomes a sum over discrete target points instead of an integral:

$$J_{\text{CVT}} = \sum_{i=1}^{N} \int_{s_i} |x - x_i|^2 dx \tag{7}$$

We now derive the velocity term v_i^{kms} by computing the gradient of the discrete K-means loss function. Taking the gradient of J with respect to x_i gives:

$$\frac{\partial J}{\partial x_i} = \sum_{t_j \in s_i} \frac{\partial}{\partial x_i} \parallel t_j - x_i \parallel^2 = \sum_{t_j \in s_i} -2(t_j - x_i) = -2\left(\sum_{t_j \in s_i} t_j - |\mathrm{s}_i| x_i\right) \tag{8}$$

Define the centroid of region s_i as $c_i = \frac{1}{|\mathrm{s}_i|} \sum_{t_j \in s_i} t_j$, then the gradient becomes:

$$\frac{\partial J}{\partial x_i} = -2|\mathrm{s}_i|(c_i - x_i) \tag{9}$$

While our implementation uses a Gaussian-weighted centroid formulation:

$$v_i^{\mathrm{re}} = \mu_2 \cdot \frac{\sum_{j\in s_i^{\mathrm{safe}}} w_{i,j}\left(\mathrm{tar}_j - p_i\right)}{\sum_{j\in s_i^{\mathrm{safe}}} w_{i,j}} \tag{10}$$

where $w_{i,j} = \exp\left(-\frac{||\mathrm{tar}_j - p_i||^2}{2\sigma^2}\right)$ is a Gaussian kernel function, this form is mathematically equivalent to a soft version of the K-means update[26]**.** Specifically, classical K-means assigns each point to its nearest cluster center (hard partitioning), while our approach performs soft assignment: each target point contributes to the centroid calculation proportionally to its proximity, determined by the Gaussian weight. In the limit as $\sigma \to 0$, this soft centroid converges to the hard centroid used in K-means. Therefore, the update direction in our implementation still approximates the negative gradient of the original K-means loss function with the centroid c_i replaced by a **smoothed estimate** from nearby target points.

To analyze the system's stability, we define a composite Lyapunov function $V(x)$ over the full state $x = [x_1^\top, \dots, x_N^\top]^\top$:

$$V(x) = \alpha_1 V_1(x) + \alpha_2 V_2(x) + \alpha_3 V_3(x) \tag{11}$$

where:

- $V_1(x) = \sum_{i=1}^N |x_i - c_i|^2$: total squared distance from robot positions to the centroids of their assigned regions.
- $V_2(x) = \sum_{i=1}^N \sum_{j\in\mathcal{N}_i}\left(|x_i - x_j| - \bar{l}\right)^2$: deviation from uniform neighbor distances.
- $V_3(x) = \sum_{i=1}^N (|\mathrm{s}_i| - \bar{\omega})^2$: deviation in region sizes.

Here, $\bar{l}$ is the consensus target inter-robot distance, $|\mathrm{s}_i|$ is the number of target points in robot i's region, and $\bar{\omega} = M/N$ is the average. Each $V_k(x)$ is positive semi-definite, so the composite function satisfies $V(x) \geq 0$ for all x.

To prove stability, we analyze the time derivative $\dot{V}(x)$ along the system trajectory:

$$\dot{V}(x) = \sum_{i=1}^{N} \nabla_{x_i} V(x)^\top \dot{x}_i \tag{12}$$

Each component is designed as a negative gradient flow:

- $v_i^{\mathrm{re}} = -k_c \nabla_{x_i} |x_i - c_i|^2$
- $v_i^{\mathrm{rb}} = -k_s \nabla_{x_i} (|s_i| - \bar{\omega})^2$
- $v_i^{\mathrm{dis}} = -k_l \nabla_{x_i} \sum_{j\in\mathcal{N}_i}\left(|x_i - x_j| - \bar{l}\right)^2$

Thus,

$$\dot{V}(x) = \sum_{i=1}^{N}\left(-k_c|\nabla_{x_i}V_1|^2 - k_l|\nabla_{x_i}V_2|^2 - k_s|\nabla_{x_i}V_3|^2\right) \leq 0 \tag{13}$$

This implies that $V(x)$ is non-increasing over time. Since $V(x)$ is bounded below (by 0), and

$\dot{V}(x) \leq 0$, the function converges.

2.2 Convergence Under Double-Integrator Dynamics

In Sec. 2.1, we analyzed the convergence of the proposed formation law under a single-integrator kinematic model. In both AirSim and Crazyflie experiments, however, we command velocity setpoints. The onboard low-level controller then generates thrust/attitude commands to track the desired velocity, which is naturally modeled at the outer-loop level as a double-integrator with a velocity PI tracking loop. In this section, we provide a rigorous convergence analysis under such a model.

We consider the double-integrator translational model:

$$\dot{x}_i = v_i, \dot{v}_i = a_i. \tag{14}$$

We define the composite "formation energy" (same structure as Sec. 2.1):

$$\Phi(x) \triangleq \alpha_1 V_1(x) + \alpha_2 V_2(x) + \alpha_3 V_3(x). \tag{15}$$

To track the commanded velocity $v_i^*(x)$, we consider a standard PI tracking law in acceleration form:

$$a_i = \dot{v}_i^*(x) - k_p v_i - v_i^*(x) - k_i z_i, \dot{z}_i = v_i - v_i^*(x). \tag{16}$$

where $k_p > 0$ and $k_i > 0$ are PI gains, and $z_i \in \mathbb{R}^d$ is the integrator state. Define the velocity tracking error $e_i \triangleq v_i - v_i^*(x)$. Then, using $\dot{e}_i$ and the above PI law, we get the closed-loop error dynamics:

$$\dot{e}_i = -k_p e_i - k_i z_i, \dot{z}_i = e_i, \dot{x}_i = v_i = v_i^*(x) + e_i. \tag{17}$$

We consider the Lyapunov candidate:

$$\mathcal{L}(x, e, z) \triangleq \Phi(x) + \frac{1}{2}\sum_{i=1}^{N} \| e_i \|^2 + \frac{k_i}{2}\sum_{i=1}^{N} \| z_i \|^2. \tag{18}$$

The time derivative of the error energy:

$$\frac{d}{dt}\left(\frac{1}{2}\sum_{i=1}^{N} \| e_i \|^2\right) = \sum_{i=1}^{N} e_i^\top \dot{e}_i = \sum_{i=1}^{N} e_i^\top(-k_p e_i - k_i z_i) = -k_p \sum_{i=1}^{N} \| e_i \|^2 - k_i \sum_{i=1}^{N} e_i^\top z_i. \tag{19}$$

The time derivative of the integrator energy:

$$\frac{d}{dt}\left(\frac{k_i}{2}\sum_{i=1}^{N} \| z_i \|^2\right) = k_i \sum_{i=1}^{N} z_i^\top \dot{z}_i = k_i \sum_{i=1}^{N} z_i^\top e_i = k_i \sum_{i=1}^{N} e_i^\top z_i. \tag{20}$$

The cross terms $\pm k_i \sum e_i^\top z_i$ cancel exactly. Therefore, the derivative of $\mathcal{L}$ is:

$$\dot{\mathcal{L}} = \dot{\Phi}(x) - k_p \| e \|^2 \leqslant 0 \tag{21}$$

Therefore, in the second-order case, our method still converges.

2.3 Experimental Analysis

To complement the Lyapunov-based theoretical analysis, we conduct a series of experiments to empirically evaluate the stability of the proposed swarm formation algorithm under various perturbations and conditions. These experiments aim to demonstrate the system's convergence behavior, robustness to noise, and resilience to initial condition variations. Since the structural details

of complex 3D shapes are often difficult to visually discern, we adopt 2D shapes in our stability experiments to more clearly illustrate the formation process and algorithmic behavior.

2.3.1 Convergence from Random Initialization

Goal: Validate that the swarm converges from disordered states to structured formations consistently.
Experiment: Initialize the swarm in randomized 3D positions within a bounding volume significantly larger than the target shape. Track the evolution of the swarm's configuration over time. As shown in Fig 3 and Fig 4, in all scenarios, the swarm gradually converges to the desired target shape. Despite differences in the initial coverage rate, entering rate, and spatial uniformity across scenarios, the swarm consistently converges to nearly identical final configurations. Throughout this process, safety is preserved by enforcing inter-robot distance constraints that prevent collisions, even in dense or highly perturbed initial arrangements. These results demonstrate that the algorithm not only achieves shape convergence reliably, but also exhibits strong adaptivity and safety guarantees under a broad range of initialization conditions.

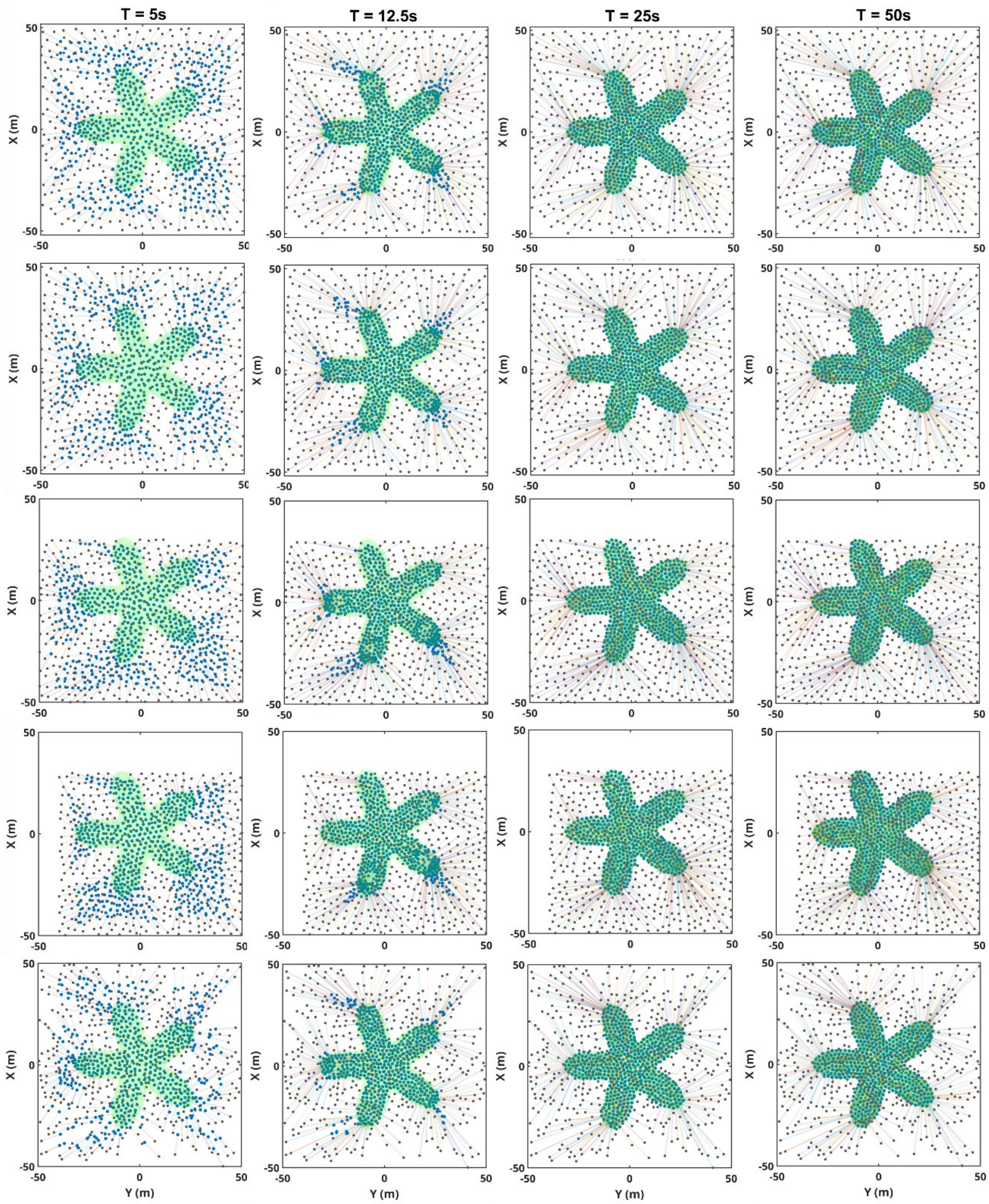

Fig 3 | Snapshots of the swarm at multiple timestamps (t= 5s, t = 12.5s, t = 25s, t= 50s). Each row corresponds to a different initialization strategy for 500 robots, gray points are initial positions of the swarm. Rows 1–2: Robots are randomly initialized in 3D space with a minimum inter-robot distance of 3 meters. Rows 3–4: Initial positions are restricted to anisotropic spatial bounds to induce uneven spatial distribution. Row 5: Robots are randomly initialized with a reduced minimum spacing of 2 meters.

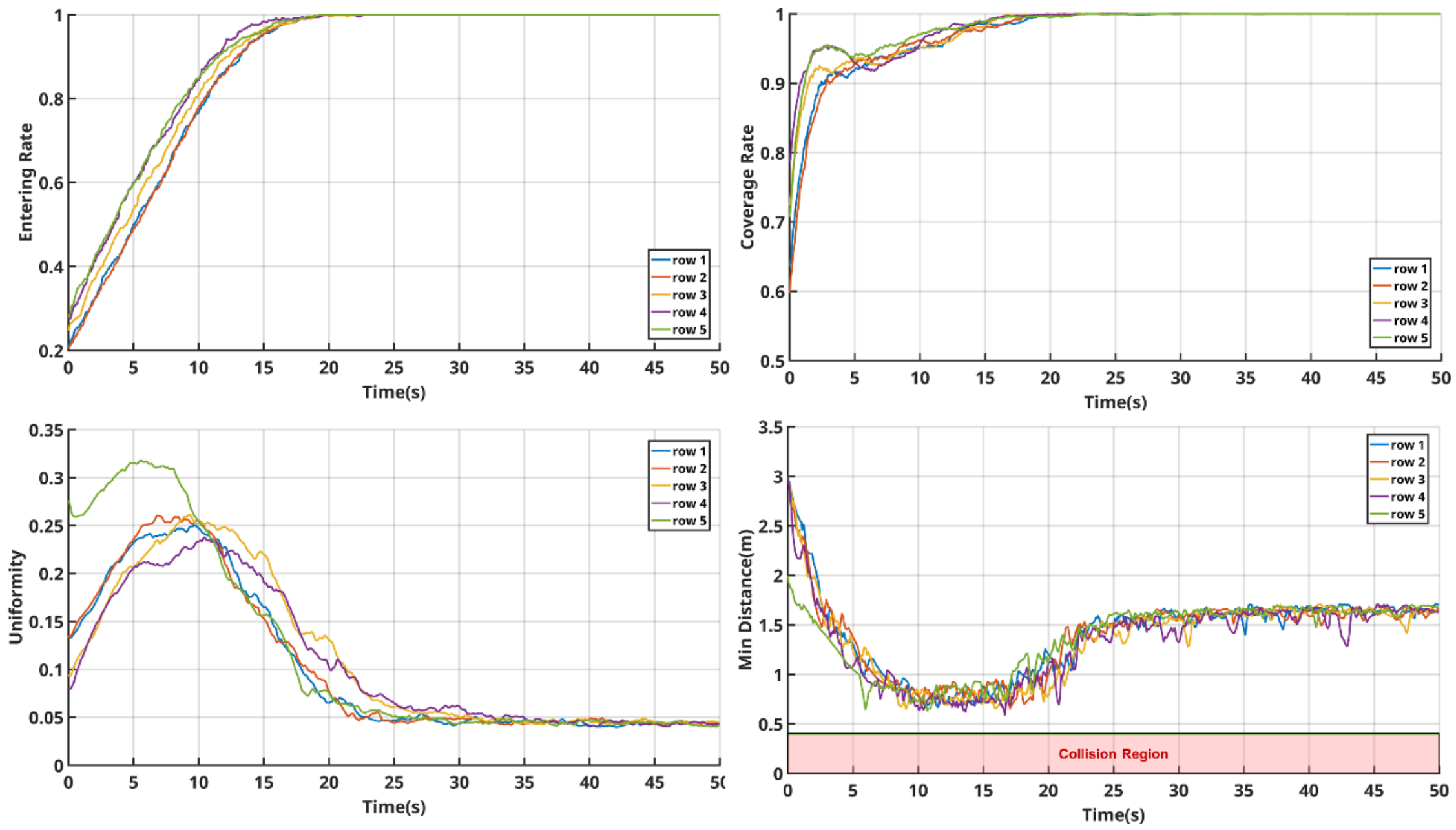

Fig 4 | Quantitative evaluation of swarm convergence under five different initialization settings. The plots track key metrics over time, including entering rate, coverage rate, uniformity, and minimum inter-robot distance. Despite noticeable differences in early-stage behavior due to varying initial conditions, all configurations ultimately achieve full coverage and shape entry, while maintaining safe inter-robot distances and improving spatial uniformity. These results confirm the algorithm's consistent stability, safety, and adaptability across diverse starting scenarios.

2.3.2 Robustness to Measurement Errors

Goal: Evaluate the impact of sensor and localization noise on the swarm's ability to stably form target shapes under realistic sensing conditions.

Experiment: Both Gaussian and uniform random noise are simultaneously injected into each robot's measurements of its own position and the positions of its neighbors. Formally, the observed (noisy) position vector $\tilde{x}_i$ used in control computation is given by:

$$\tilde{x}_i = x_i + \epsilon_i^{\mathcal{N}} + \epsilon_i^{\mathcal{U}} \tag{22}$$

where $\epsilon_i^{\mathcal{N}} \sim \mathcal{N}(0, \sigma^2 I)$ is the **Gaussian noise** term with zero mean and standard deviation σ, modeling random disturbances such as sensor fluctuations and environmental uncertainty. $\epsilon_i^{\mathcal{U}} \sim \mathcal{U}(-\delta, \delta)^2$ is the **uniform noise** term sampled independently in each dimension from a bounded interval. This simulates practical sensing disturbances such as cumulative drift, quantization effects, or non-Gaussian errors in distributed localization systems. Several trials are conducted under different noise intensities to evaluate the method's robustness.

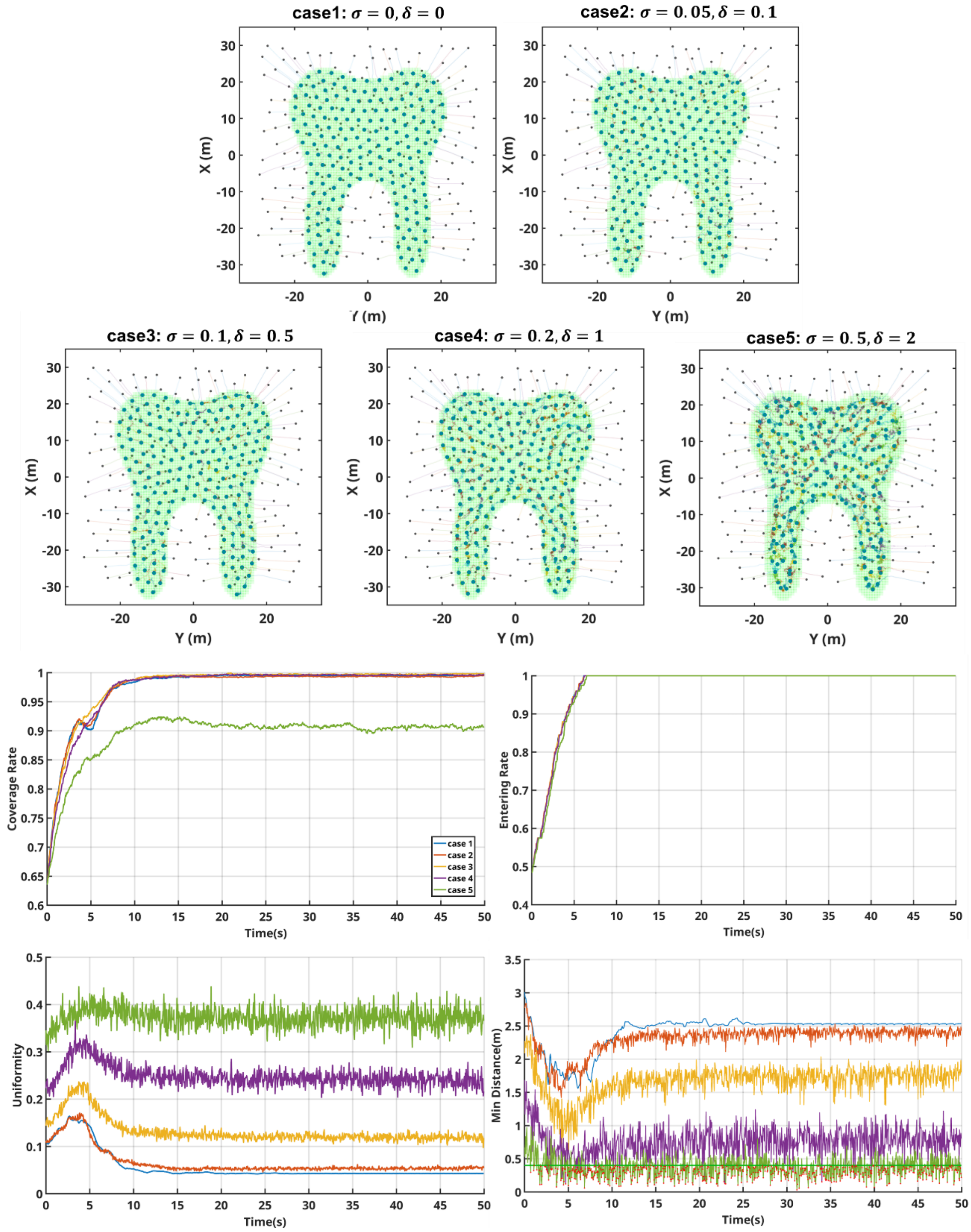

Fig 5 | Swarm performance under combined Gaussian and uniform measurement noise. Top: 200 robots' final formations under increasing noise levels. Bottom: Corresponding coverage rate, entering rate, uniformity, and minimum distance metrics over time.

Fig. 5 summarizes the impact of measurement noise on formation performance. As the noise level increases, the spatial uniformity degrades slightly, but the swarm still converges to the target shape. When the combined localization error remains within 0.5 m, the swarm is still able to successfully

complete the shape assembly with high accuracy.

2.3.3 Resilience to Local Failures

Goal: Evaluate the swarm's ability to maintain and restore formation integrity when a subset of robots are in failure or are removed during operation.

Experiment: A group of 18 drones is initialized to form a 3D spherical configuration. As show in Fig 6, at regular intervals, a pair of robots are removed from the system to simulate local failures or dropout events. No additional replanning or global coordination is introduced. The remaining drones autonomously adjust their positions in response to the topology change.

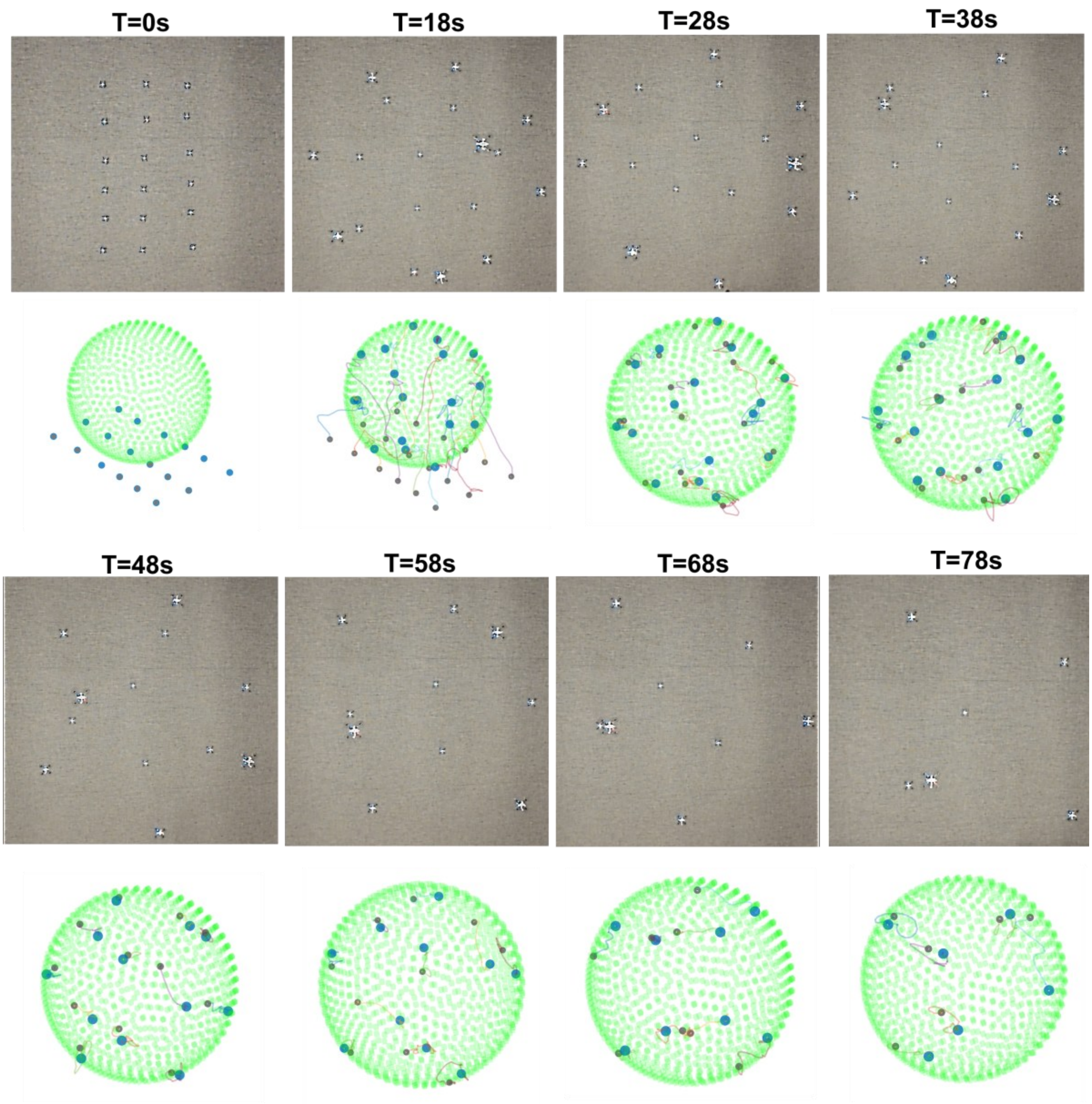

Fig. 6 | Resilience of the swarm to local failures during 3D spherical formation. A spherical formation of 18 drones remains coherent as pairs of drones are progressively removed. The remaining drones autonomously reorganize to restore the shape, demonstrating self-repair and formation stability.

3 Assembling Diverse 3D Shapes

3.1 Construction of Target 3D Shapes

To enable the design and testing of diverse 3D formation targets, we developed a block-based visual modeling tool inspired by the open-source Minecraft [错误!未找到引用源。] framework, as shown in Fig 7. The system provides an interactive interface where users can build 3D voxel-based shapes by stacking virtual blocks, similar to constructing structures in Minecraft. This allows intuitive specification of complex volumetric geometries using discrete spatial grids.

Once a desired shape is built, the system automatically extracts the coordinates of occupied voxels, which are then used as the target point cloud for the swarm formation algorithm. In addition to manual construction, the tool also supports importing existing 3D models in standard formats such as .stl and .obj, enabling convenient reuse of real-world or CAD-designed shapes.

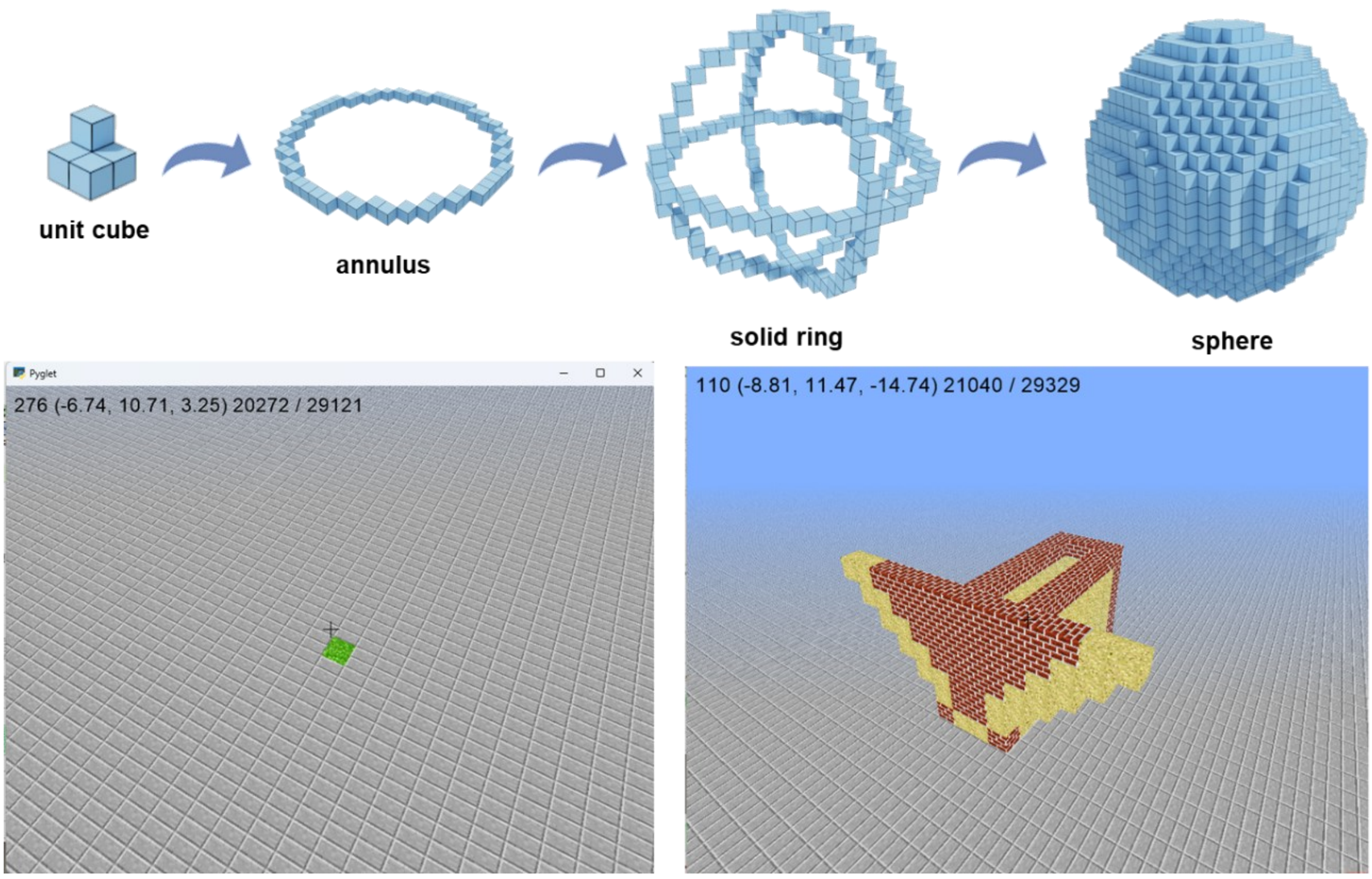

Fig. 7 | Visual interface for constructing 3D shapes. Users can intuitively build complex voxel-based shapes by stacking blocks in a Minecraft-inspired editor. The interface supports both manual design and shape evolution through simple operations, offering a beginner-friendly and flexible way to generate diverse 3D targets for swarm formation.

3.2 Geometric Swarm Assembly Reflecting Bubble-Raft Principles

To evaluate the generalization capability of the formation algorithm across different structural complexities, we categorize target 3D shapes into three representative geometric classes:

Surface-like structures: These shapes are defined primarily by their outer surfaces, with little or

no internal volume. Typical examples include spherical shells, hollow cubes, and open domes.

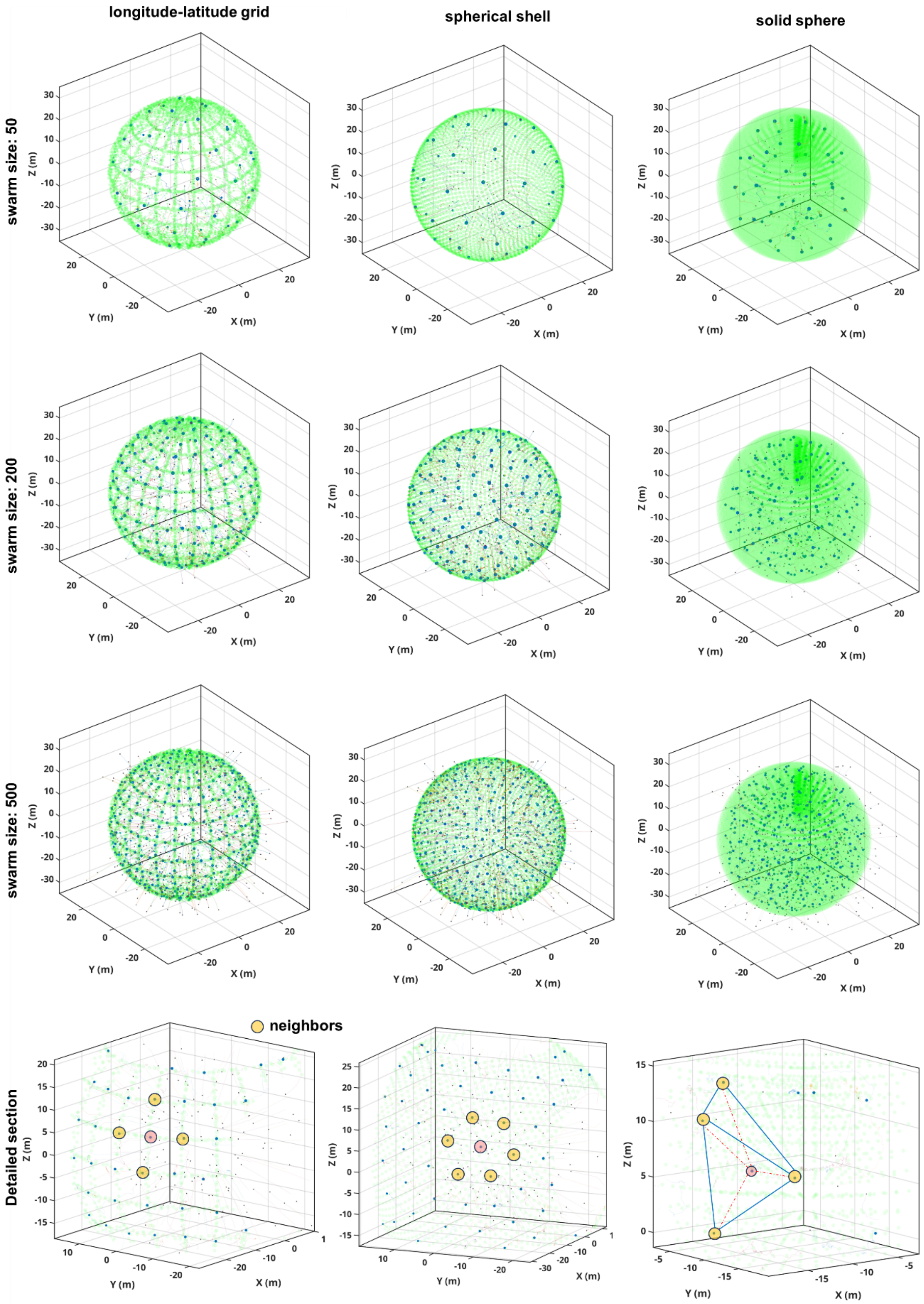

Fig. 8 | Assembly of swarm into three categories of 3D geometric structures. From left to right: a longitude-latitude grid, a spherical shell, and a solid sphere. Each row corresponds to a different swarm size

(500, 1000, 1500 robots). The bottom row visualizes local interactions and neighborhood structures for representative robots in each configuration, highlighting how topology influences spatial coordination and connectivity.

Assembly of such shapes requires the swarm to accurately conform to curved or planar boundaries while maintaining even distribution across the surface.

Line-like structures: These shapes are built around extended, narrow forms resembling curves, chains, or skeletal frames. Examples include spirals, lattices, or branching loops. These configurations test the algorithm's ability to maintain structural continuity and connectivity with minimal volume support.

Volume-filling (solid) structures: These are dense 3D shapes such as solid cubes, spheres, pyramids, or biologically inspired masses. Formation of such targets evaluates how well the swarm can manage interior packing, avoid collisions, and distribute uniformly in dense environments.

We design a set of representative shapes within each category, ranging from simple geometric primitives to more complex forms. By testing across this diverse set, we aim to validate the algorithm's adaptability to a broad class of 3D swarm configurations. We first construct a set of representative shapes that progressively span different classes of 3D spatial complexity. Specifically, we use a longitude-latitude grid structure to represent a line-like configuration, a spherical shell to represent a surface-based shape, and a solid sphere to represent a fully volumetric form. These three shapes share a common global geometry but differ in their internal distribution constraints and structural density.

As shown in Fig. 8, these three structures reflect fundamental modes of spatial organization in 3D swarm assemblies. In the longitude-latitude grid, robots are arranged along intersecting lines, and their local neighborhood relationships are determined by linear connectivity. The resulting configuration exhibits uniform inter-robot distances along the grid lines, with robots aligned in continuous curves.

In contrast, the spherical shell exhibits surface tiling behavior: each robot is typically surrounded by six neighbors, forming locally hexagonal patterns. This results in a globally curved but locally planar structure, where the smallest repeating unit is a set of equilateral triangles.

In the solid sphere, local structure becomes more complex due to full 3D volumetric packing. Robots tend to arrange themselves into locally tetrahedral configurations—each robot connected to four neighbors forming a regular tetrahedron. However, since a perfect space-filling tessellation of regular tetrahedra is mathematically impossible, the final structure consists of a mix of distorted polyhedra. This behavior closely resembles the internal organization of **3D bubble rafts** in nature, where local energy minimization leads to irregular yet stable polyhedral packing.

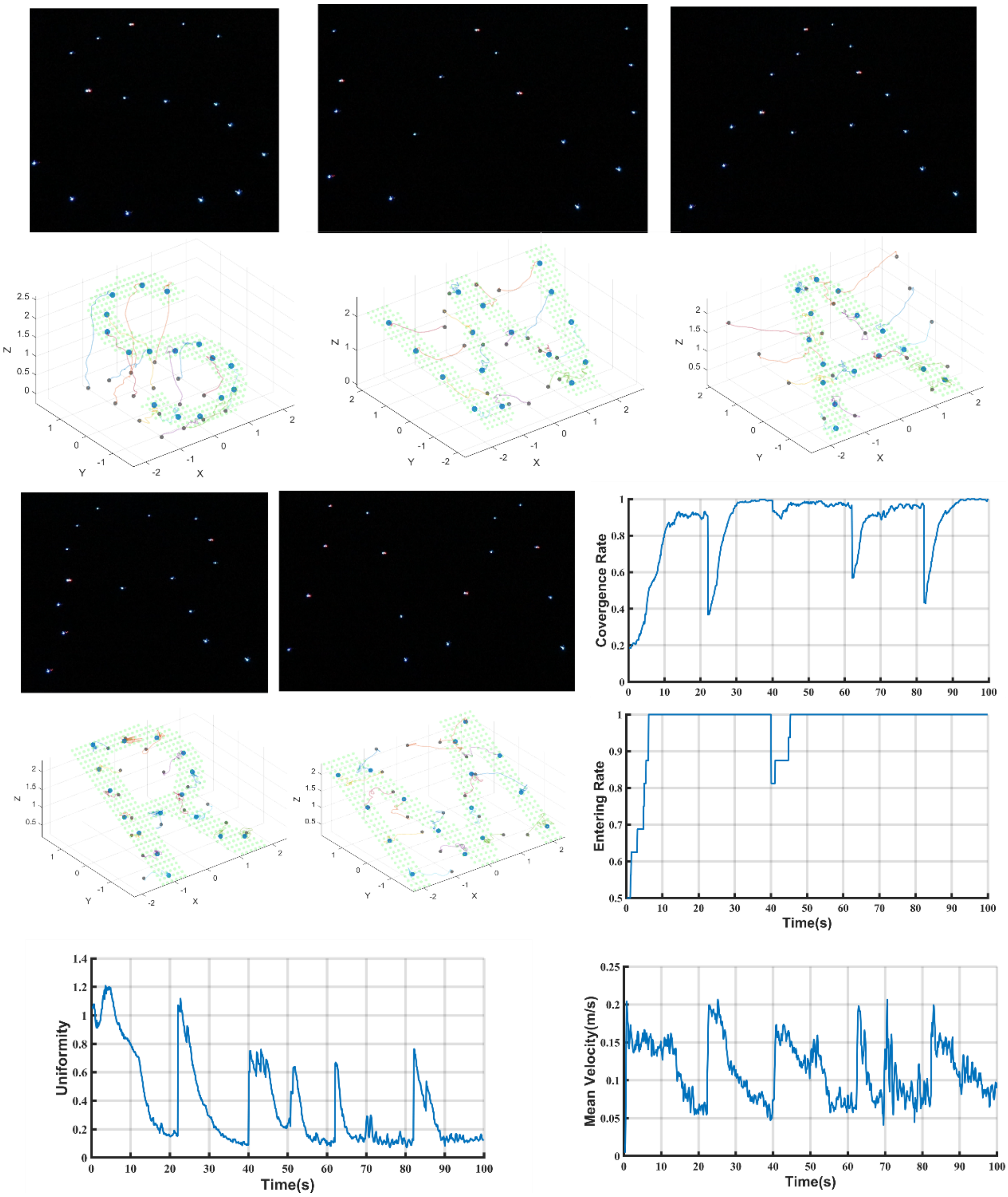

Fig. 9 | Real-world 3D drone formation transitions spelling "SWARM". The drone swarm sequentially transitions through five target letter shapes. Top: real flight snapshots and corresponding 3D reconstructions. Bottom: quantitative metrics including coverage rate, entering rate, uniformity, and mean velocity.

3.3 Real-World Demonstration of 3D Formation Transitions

To validate the algorithm's practicality and real-time performance in physical environments, we conduct a real-world drone experiment demonstrating dynamic 3D formation switching. In this setup, a group of quadrotor drones performs sequential transitions through a series of distinct 3D letter-shaped formations spelling the word **"SWARM"**.

Each letter formation (S, W, A, R, M) is defined as a set of spatial target points in 3D space. The

drones are assigned new target positions for each letter and must reconfigure their formation while maintaining inter-robot spacing. As shown in Fig 9, the transition between letters is performed smoothly, without external intervention, highlighting the system's ability to adaptively reorganize under changing high-level objectives.

3.4 Drone Show

To further demonstrate the expressive capacity and aesthetic potential of our shape assembly algorithm, we conducted a series of drone show experiments in the AirSim[错误!未找到引用源。] simulator, built on Unreal Engine. In these tests, swarms of drones were coordinated to form complex, recognizable 3D figures including a llama (alpaca), a double-helix DNA structure, and a three-dimensional rose. These examples go beyond geometric primitives to highlight the algorithm's ability to manage fine structural details, non-uniform densities, and layered depth within arbitrary shapes. Despite the increased visual complexity, the swarm maintains stable formations through the same decentralized control mechanism, ensuring safe inter-robot spacing and coherent global structure. As shown in Fig 10-12, the drone show illustrates the potential of our system for artistic displays and creative robotics, where both form and safety must be simultaneously guaranteed.

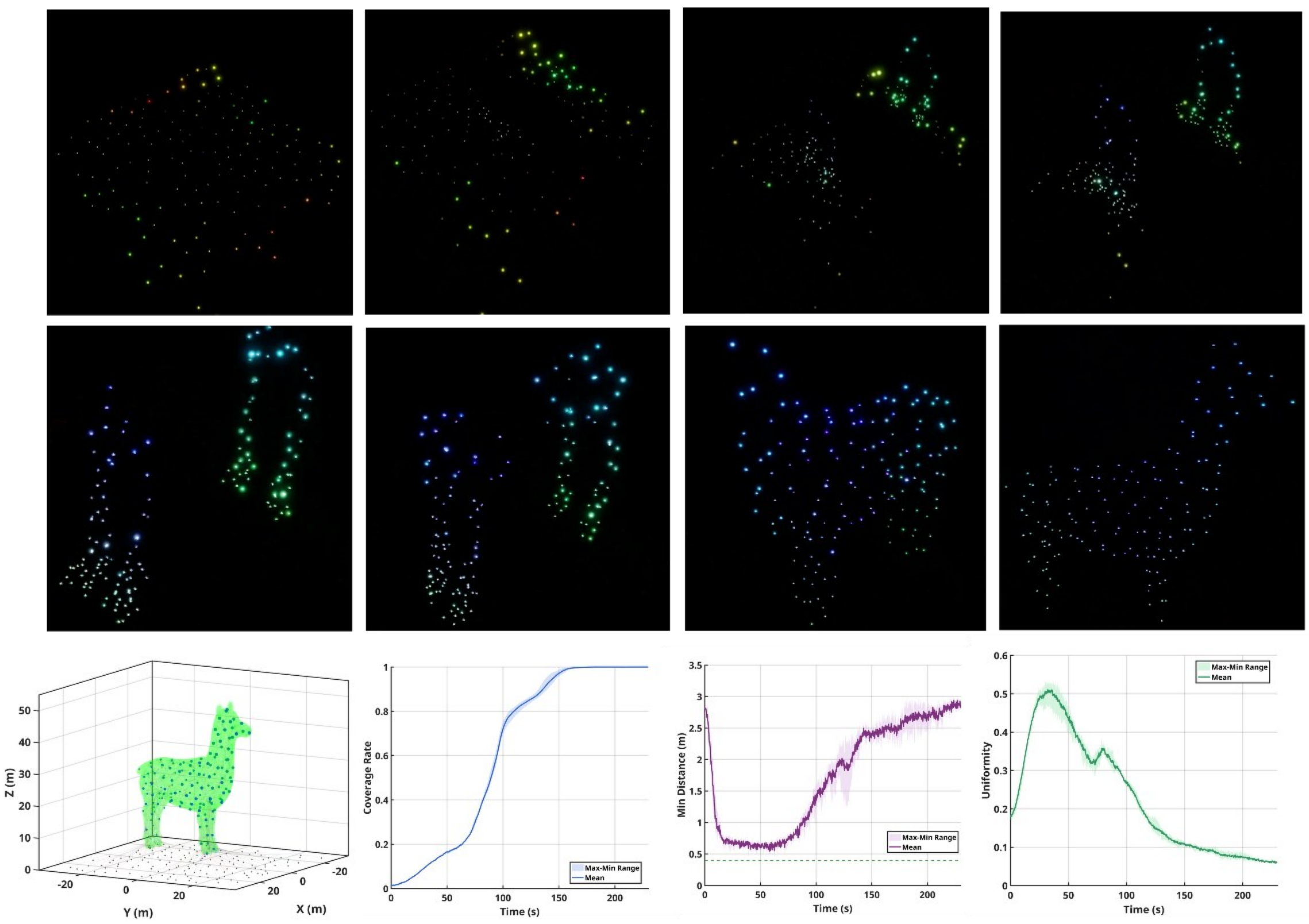

Fig 10 | A 200-drone swarm forming a 3D alpaca shape in simulation. Top: sequential snapshots of the formation process rendered in AirSim, showing the swarm gradually assembling into a recognizable alpaca. Bottom: 3D target shape model and performance metrics, including coverage rate, minimum inter-robot

distance, and spatial uniformity over time. Each metric curve includes the mean and max–min range computed from five independent trials with different initial positions.

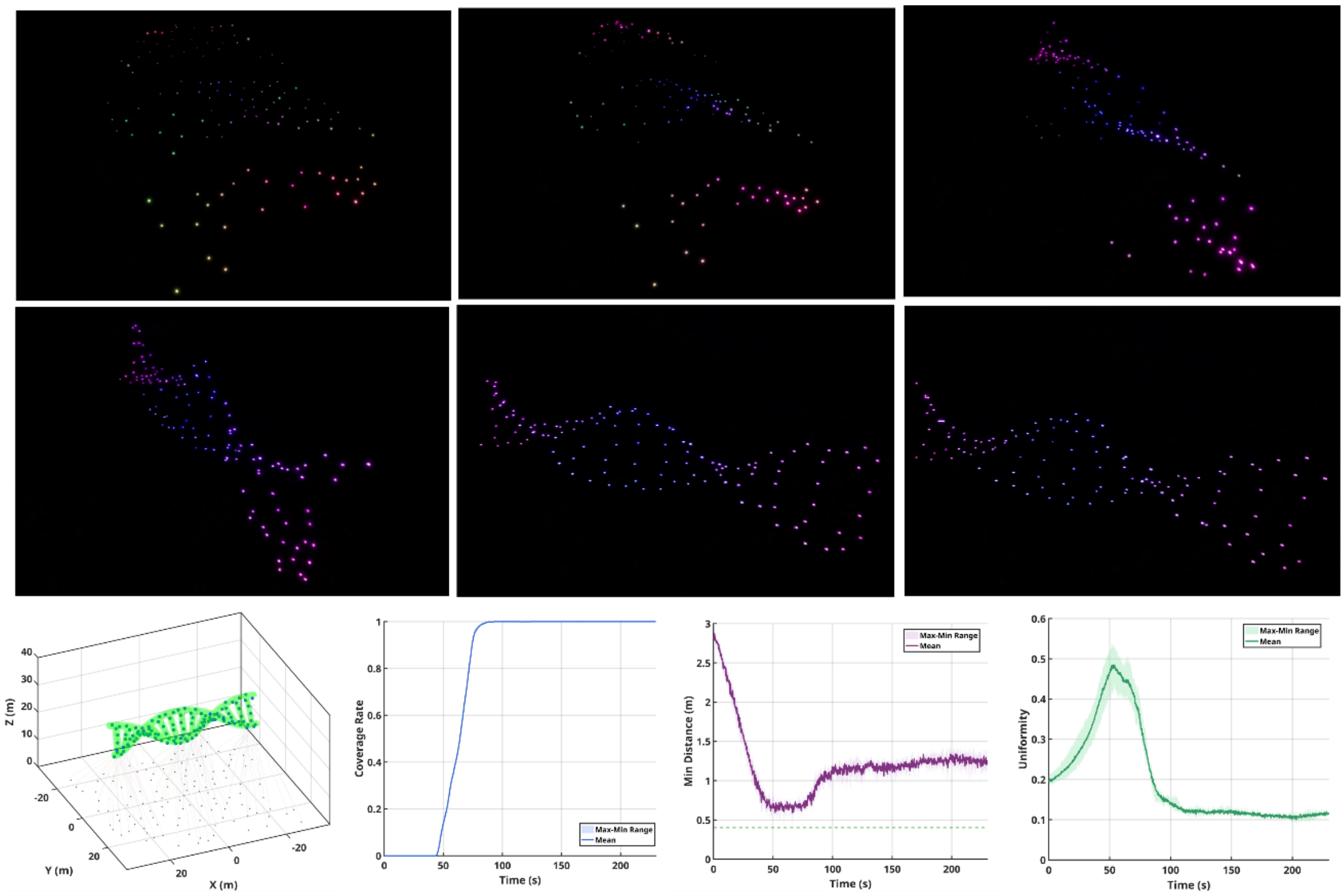

Fig. 11 | A 200-drone swarm forming a 3D DNA double helix. Top: formation process in simulation. Bottom: target model and metrics showing high coverage, safe spacing, and stable uniformity throughout the assembly from five independent trials with different initial positions.

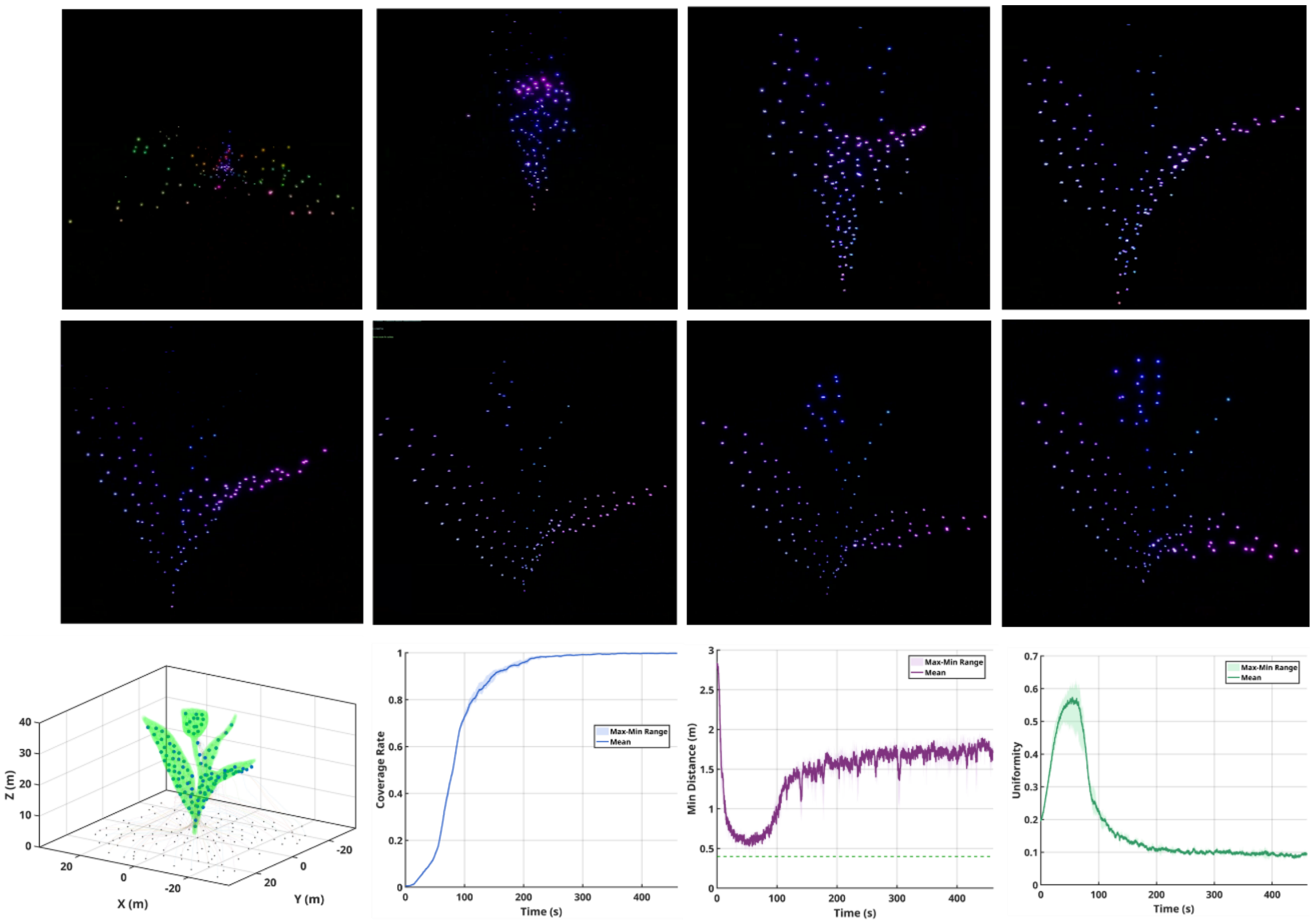

Fig. 12 | A 200-drone swarm forming a 3D rose shape.

4 More Comparison with Mean-shift Strategy

In this section, we focus our comparative analysis primarily on the Mean-shift strategy, which represents a well-established baseline for density-driven swarm formation. While we briefly included Image Moment and Graph Similarity in our initial benchmarking, we will not further analyze them here for two key reasons. First, Image Moment is largely limited to symmetric configurations, and does not generalize well to arbitrary or volumetric shapes[27]. Second, Graph Similarity incurs prohibitive computational costs due to its global structure matching[28], making it impractical for large-scale or real-time swarm applications.

In addition, the original Mean-shift implementation was developed in MATLAB and relies heavily on large-scale matrix computations to generate control commands—for instance, storing the target shape as a dense matrix and exhaustively computing each robot's sensing region. While conceptually elegant, this implementation is computationally inefficient for large-scale, real-time applications. In contrast, our framework employs KD-tree-based neighbor queries [错误!未找到引用源。] for fast local interaction processing, significantly improving runtime performance.

To enable a fair comparison, we re-implemented the Mean-shift algorithm within our own codebase. Our implementation faithfully reproduces the key modules described in the original paper, including shape negotiation, entering command, exploration command, and interaction command.

This adapted version was used in all comparative experiments shown in main text Fig. 3, ensuring consistency in the simulation environment and evaluation metrics. Both our method and the Mean-shift baseline were tested under the same infrastructure, and all source code has been made publicly available to support transparency and reproducibility.

4.1 Parameter Sensitivity

4.1.1 Effect of Sensing Range r_{sense}

We first evaluate the impact of sensing range—defined as the maximum distance within which a robot can perceive and interact with other robots—on the overall shape assembly performance. Our results show that when the sensing range exceeds 2 meters, our proposed method consistently achieves high performance across all tested configurations. In contrast, the Mean-shift strategy exhibits a performance peak when the sensing range is around 5 meters, beyond which its effectiveness drops significantly. As shown in Fig. 13, Mean-shift begins to form undesired localized clusters and fails to cover the full shape as the sensing range increases further.

This phenomenon can be explained by the behavior of Mean-shift under varying sensing scopes. At large sensing ranges, the robot's neighborhood is no longer a circular region but rather contains contour information of the target shape. In such cases, robots tend to move toward the geometric center of their sensing region. As Fig. 14 illustrates, this behavior results in local force balance near the interior center, inhibiting global dispersion and leading to aggregation.

By contrast, at small sensing ranges, most robots only perceive approximately circular neighborhoods. In this regime, Mean-shift behaves as intended: moving toward local density gradients and avoiding overcrowded areas. However, robots near the boundary of the shape experience asymmetric sensing regions (i.e., partial circles), which again causes biased movement toward the shape interior, reinforcing peripheral accumulation and degrading uniformity.

This analysis reveals an inherent limitation of the Mean-shift approach: its effectiveness is highly sensitive to the relationship between sensing range and shape geometry. If the sensing radius exceeds the narrowest cross-section of the shape, the algorithm loses its gradient-following behavior and collapses into local modes. Conversely, overly small ranges may prevent shape completion due to limited coordination. In contrast, our algorithm adapts across a broader range of sensing distances without relying on shape symmetry or isotropic neighborhoods, demonstrating greater robustness and scalability.

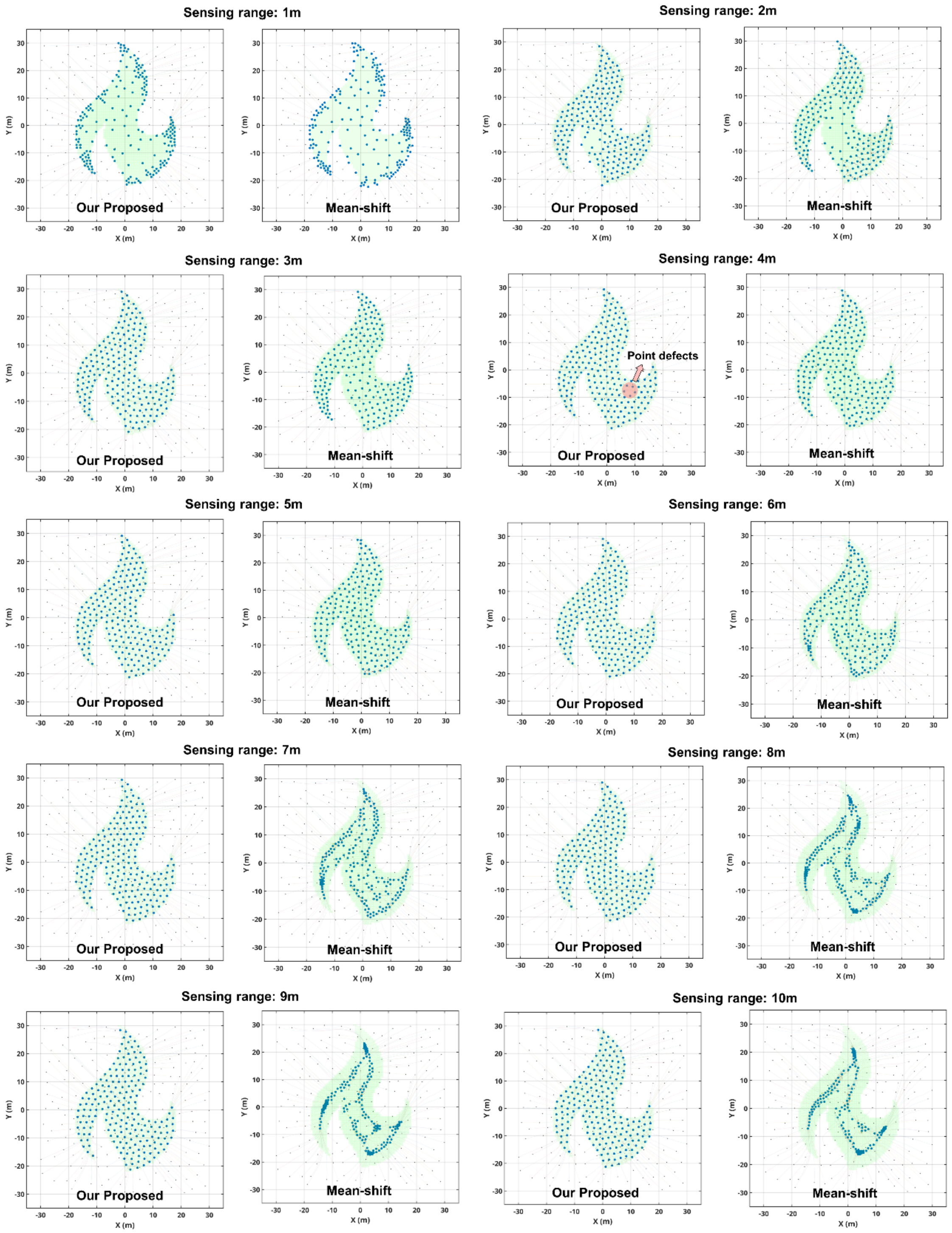

Fig. 13 | Shape assembly performance under different sensing ranges (1 m to 10 m) using 200 robots (r_{avoid} = 1.2 m). Each row compares the results of our proposed method (left) and the Mean-shift strategy (right) under increasing sensing range. While both methods perform well at moderate sensing ranges, Mean-shift shows degraded performance when the range exceeds 5 m, leading to local clustering and incomplete coverage. In contrast, our method maintains stable and accurate shape assembly across all tested settings.

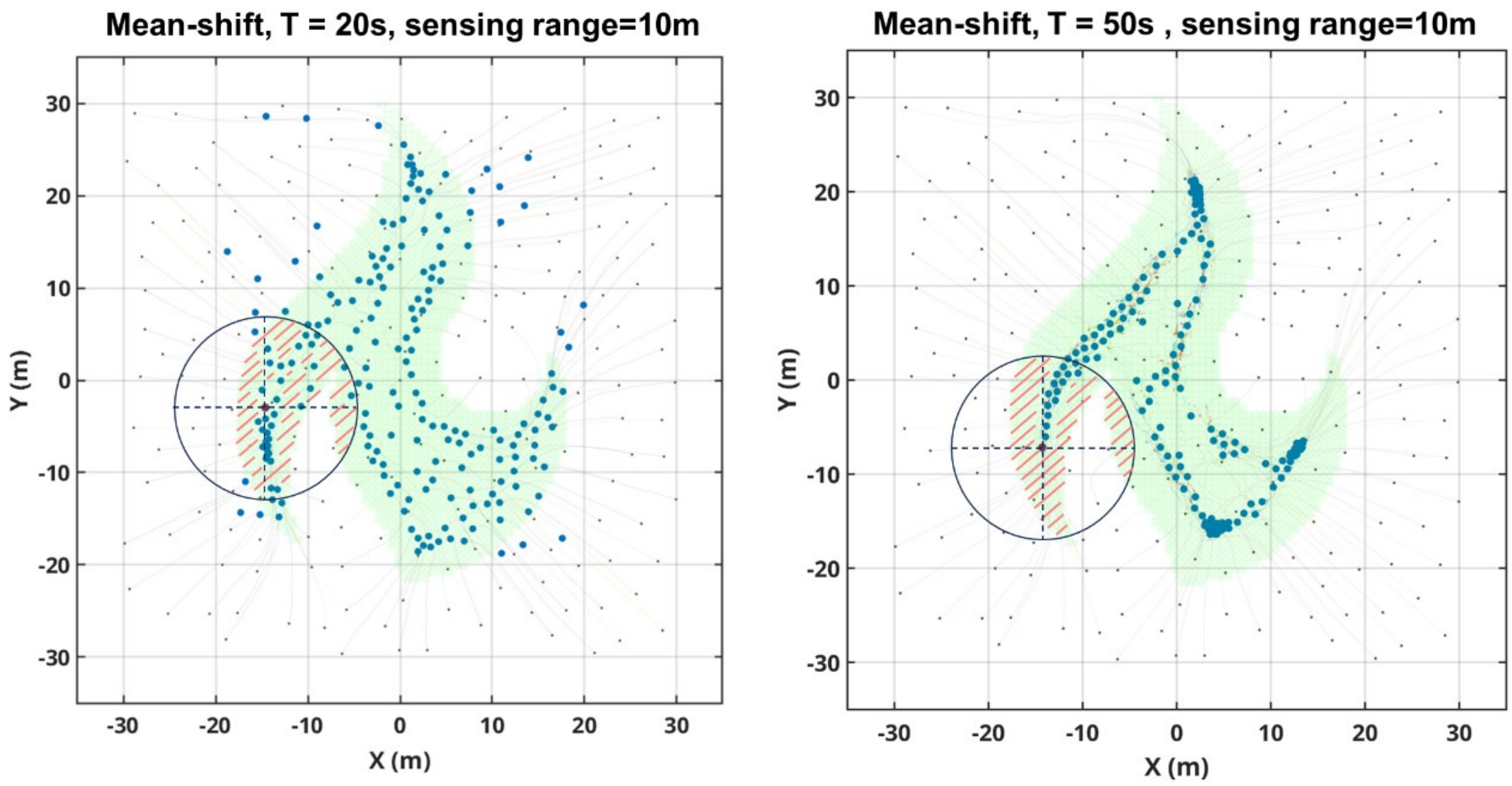

Fig 14 | The reason why Mean-shift failed shape assembly. At a sensing range of 10 m, robots perceive non-uniform, contour-shaped regions after excluding neighbors' obstacle zones (highlighted in red shading). The robot at the center of this red region experiences balanced directional inputs from all sides and thus remains stationary. As more robots fall into similar local equilibrium states, the system tends to converge prematurely toward these local centers, resulting in spatial aggregation and incomplete shape coverage.

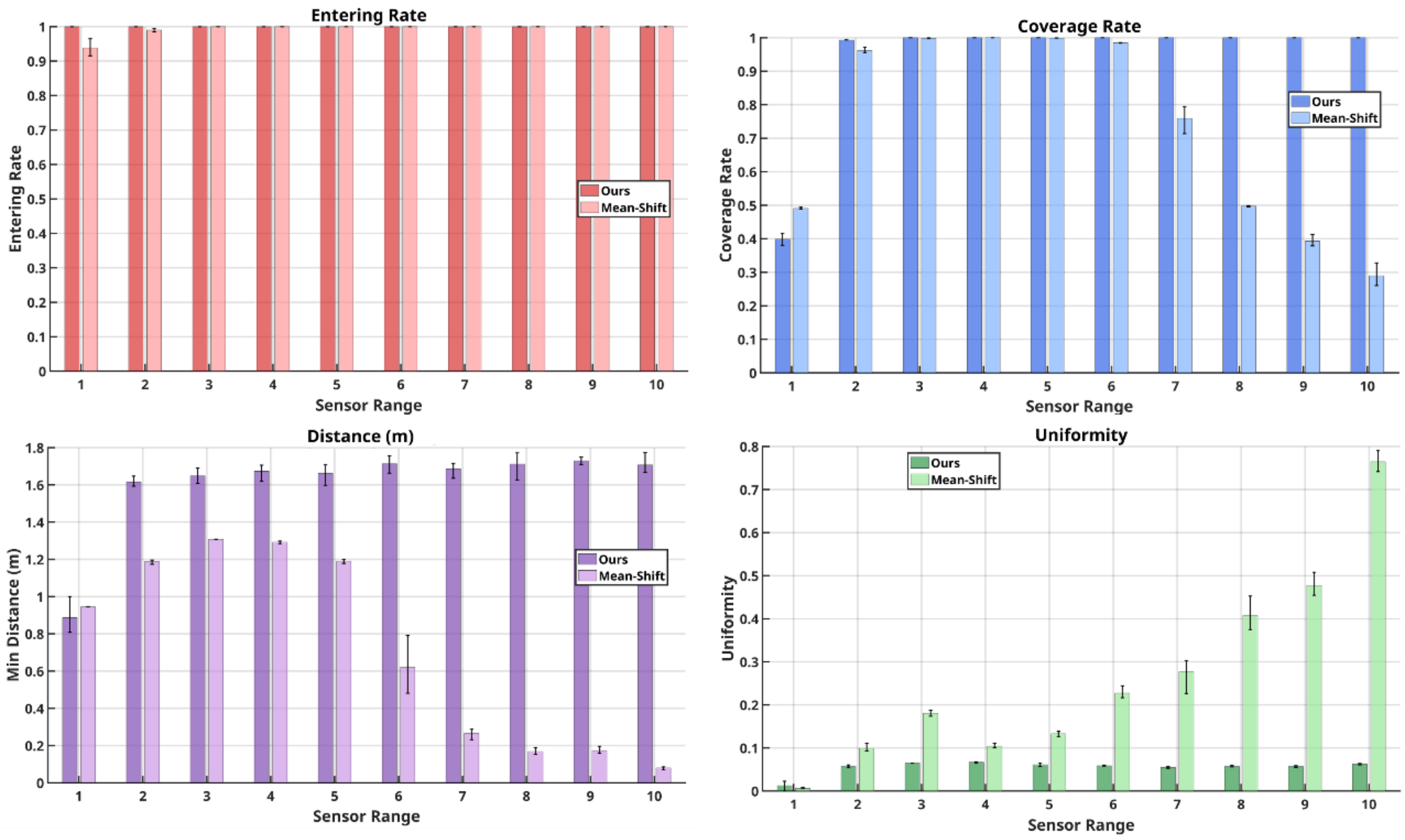

Fig 15 | Quantitative comparison between our method and Mean-shift under different sensing ranges (1 m–10 m). Metrics include entering rate (top left), coverage rate (top right), minimum inter-robot distance (bottom left), and spatial uniformity (bottom right) at convergence. Distance indicates the average minimum spacing between neighboring robots within the formed shape. Each condition was tested with 3 randomized initializations, and bars show the mean with max–min range.

4.1.2 Effect of Obstacle avoidance distance r_{avoid}

To evaluate the effect of obstacle avoidance distance r_{avoid} on formation quality and safety, we compare our method with the Mean-shift strategy under varying avoidance constraints ranging from 0 m to 2 m.

Our method consistently maintains a high-quality formation across the entire range of r_{avoid}, from 0 m to 2 m. Notably, when $r_{avoid} > 0.8$, our approach guarantees a minimum inter-robot distance exceeding 0.4 m, thereby ensuring safety without compromising formation integrity. This robustness arises from our design, which explicitly considers obstacle repulsion in the target assignment phase and maintains uniform coverage even under strict avoidance constraints.

In contrast, the Mean-shift strategy relies on emergent density differences induced by repulsion between robots. When the avoidance distance is small, these density differences are insufficient to drive robots to adequately cover the desired formation, resulting in poor formation completeness. Conversely, when r_{avoid} is large, densely packed neighboring robots severely constrain the available candidate points in the target formation. This often leads to target depletion and results in robots converging toward the centroid, causing positional conflicts and formation collapse.

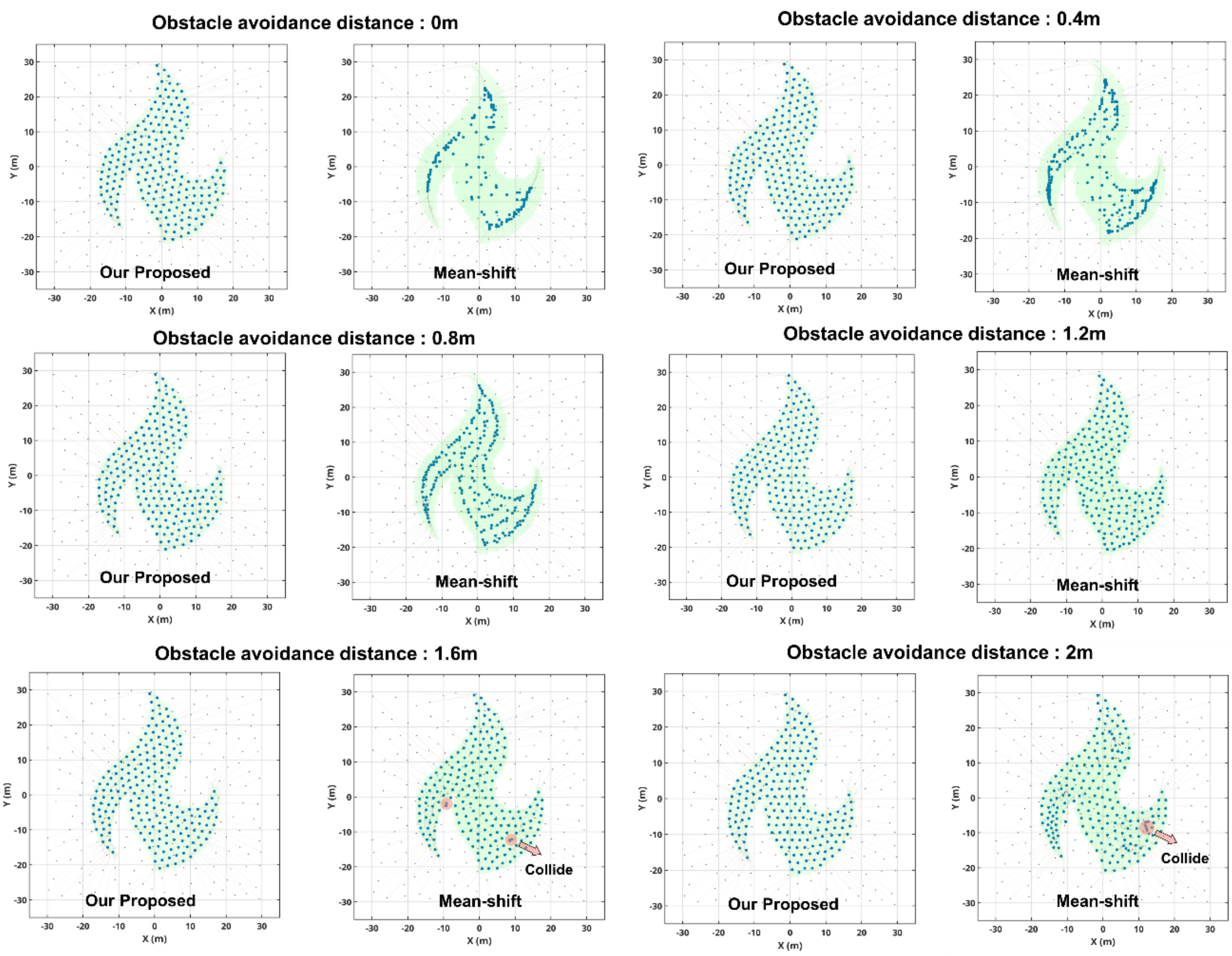

Fig. 16 | Shape assembly performance under different obstacle avoidance distances. We compare our method and the Mean-shift strategy across a range of obstacle avoidance distances (0 m to 2 m) with a fixed sensing range of 5 m. While our method consistently achieves accurate shape formation under all conditions,

Mean-shift only performs well when the avoidance distance reaches 2 m.

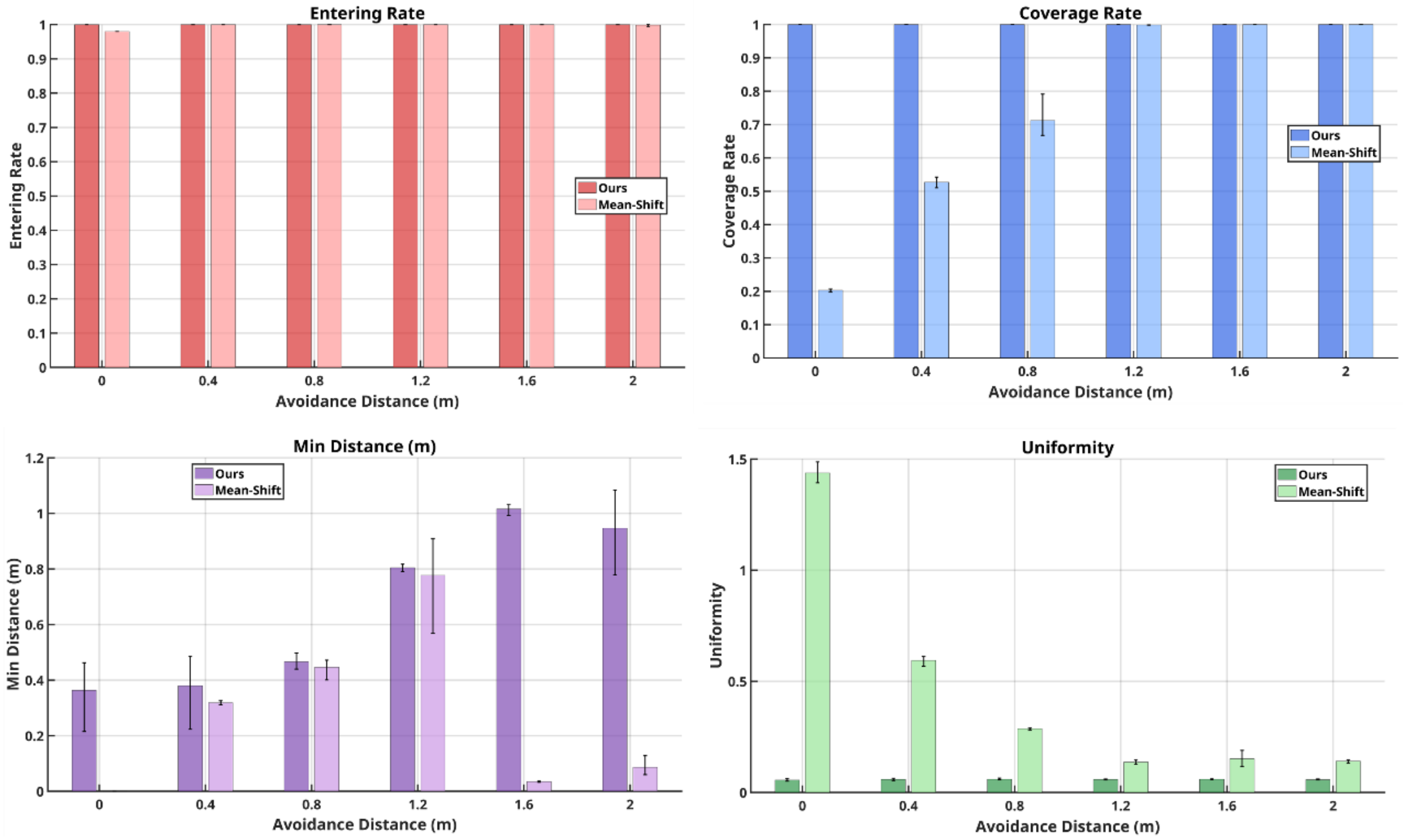

Fig. 17 | Quantitative comparison of swarm performance under different obstacle avoidance distances. We compare our method and the Mean-shift strategy across avoidance distances from 0 m to 2 m (with 0.4 m intervals). Metrics include entering rate, coverage rate, minimum inter-robot distance, and spatial uniformity.

4.1.3 Parameter Sensitive of Target Point's Density and Sensing Range

To empirically evaluate how the computational cost of the region exploration directive v^{re} scales with (i) the target-point density and (ii) the sensing range, we conduct timing benchmarks in simulation using 200 robots assembling a sphere of radius 20 m. The target shape is discretized into M target points, and each robot queries target points within its sensing range r_{sense} using a KD-tree radius query (SciPy KD-tree) to avoid full scans over all points. We report the average per-robot computation time per control iteration of v^{re}, averaged over 10 runs on a single CPU core (Intel i9-12700H).

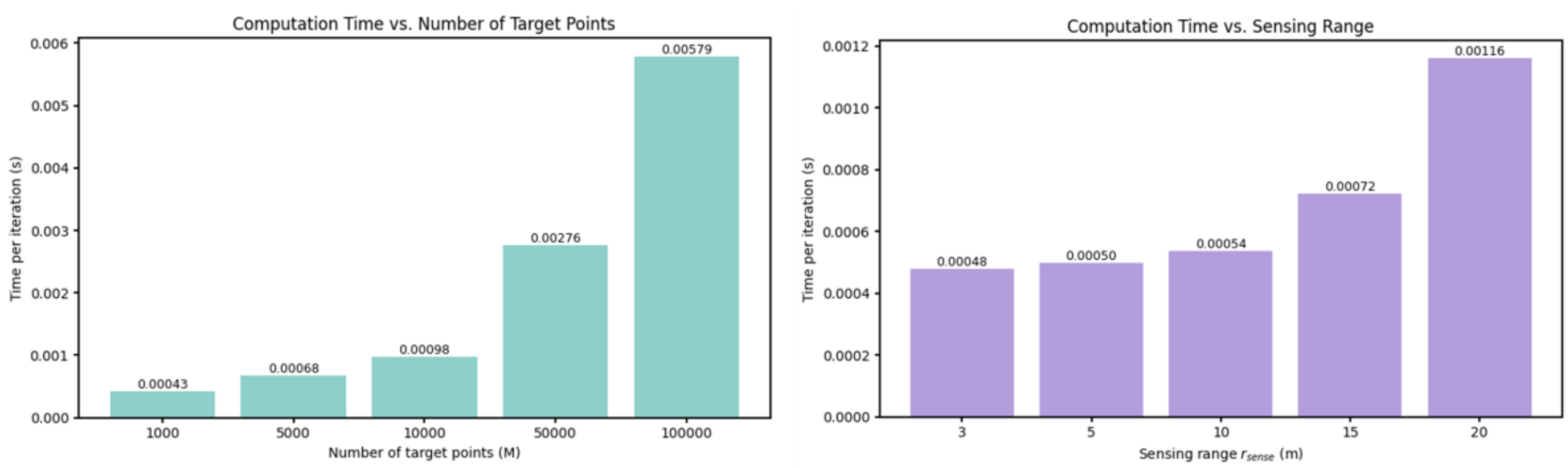

Fig. 18 | Parameter sensitivity of computation time for v^{re}. Computation time vs. number of target points (left), Computation time vs. sensing range(right).

Target-point density sensitivity. We vary the total number of target points $M \in \{1000,5000,10000, 50000,100000\}$ while keeping $r_{\text{sense}} = 10$ m fixed. As shown in Fig. 18(a), the per-iteration time increases from 0.00043 s (1k points) to 0.00579 s (100k points). This trend is consistent with the analysis that the dominant term is proportional to the expected number of local points within the sensing volume, i.e., scaling approximately linearly with the target-point density inside the sensing ball. In practice, across our experiments in main paper, we typically set the number of target points to approximately 5,000, which already provides a sufficiently dense discretization for uniform shape assembly. Even for large-scale swarms with up to 500 robots, 5,000 target points are enough to produce visually and quantitatively uniform formations

Sensing-range sensitivity. We vary the sensing range $r_{\text{sense}} \in \{3,5,10,15,20\}$m while keeping $M = 10{,}000$fixed. Fig. 14(b) shows that the per-iteration time increases from 0.00048s at 3m to 0.00116 s at 20 m. This increase matches the expectation that the number of queried points grows with the sensing volume ($\propto r_{\text{sense}}^3$), while the KD-tree overhead remains logarithmic in M. Overall, the results confirm that v^{re}remains computationally lightweight, and its runtime is primarily governed by the local point count within the sensing range.

4.1.4 Parameter Sensitive of Gaussian Kernel

We evaluate the sensitivity of the Gaussian kernel bandwidth σused in the Gaussian-weighted local destination computation. Fig 19 shows results with $\sigma \in \{0.5,1,2,4,8\}$(scaled w.r.t. the sensing range). Overall, the difference across σ is not significant: all settings lead to successful shape formation with similar final coverage and distribution quality, indicating that the system is not highly sensitive to the exact value of σ.

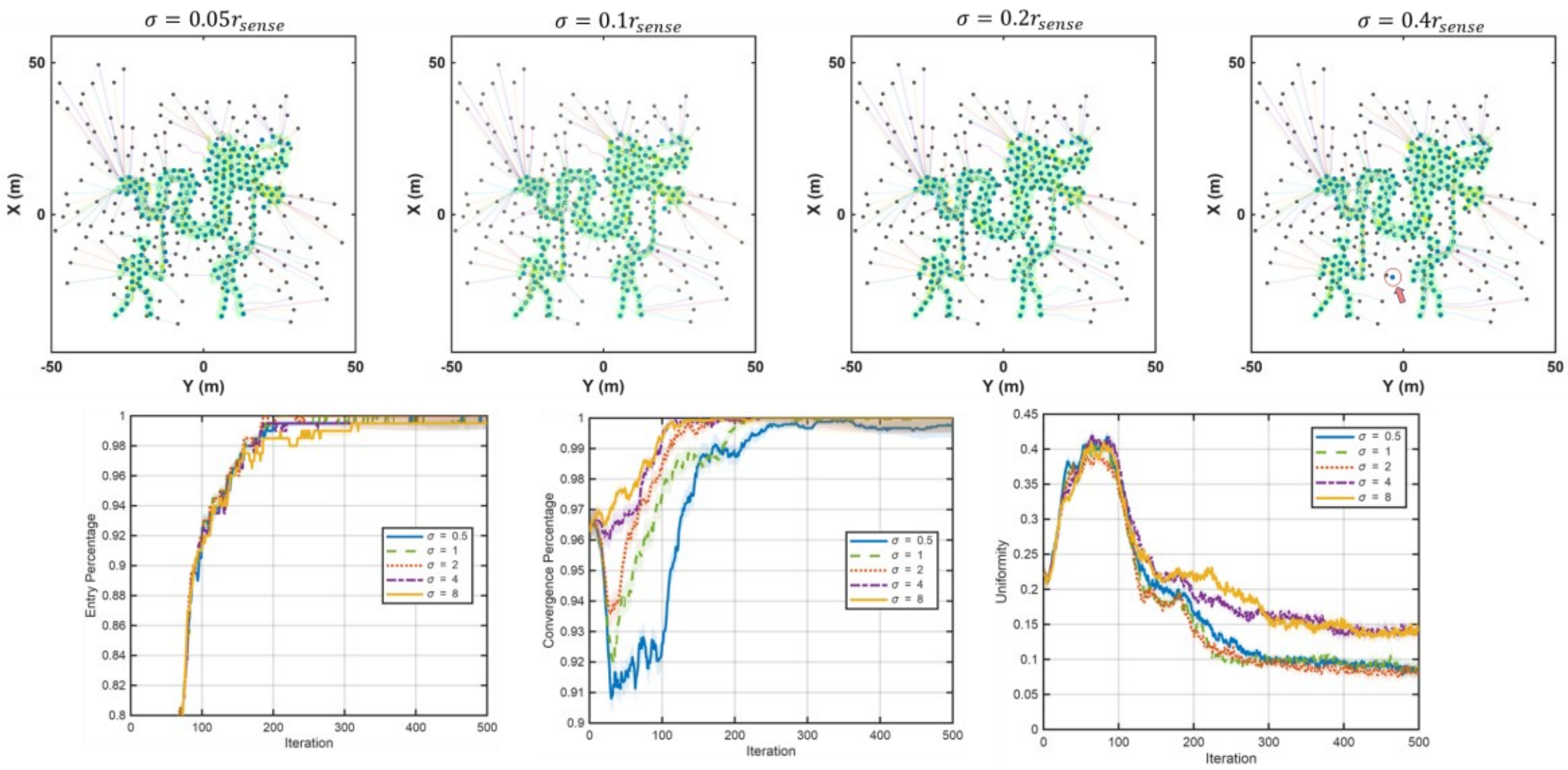

Fig. 19 | Parameter sensitivity of Gaussian Kernel Bandwidth. Shape generation (First row). Entry percentage, convergence percentage and uniformity during the process(Second row).

The main effect of $\sigma$is on the transient behavior. When $\sigma$is larger, a wider set of target points contributes to the local destination, so the destination becomes closer to the center of the robot's associated region. This

usually produces a stronger pulling effect (because more contributing points are farther away), and the swarm can reach the target shape slightly faster. However, a very large σmay also make the robot influenced by points across a broader area of a non-convex shape, which can occasionally lead to small local inconsistencies. In our results, for example, when $\sigma = 4$ and $\sigma = 8$, we observe a rare case where one robot ends up slightly outside the target shape. Importantly, this behavior is uncommon and does not change the overall convergence trend or the final macroscopic performance. In all experiment, we usually set it to $0.2 * r_{sense}$.

4.2 Assembly Performance on Challenging Shapes

To evaluate the capability of different strategies in forming complex, unevenly distributed shapes, we tested both our method and the Mean-shift approach on two highly challenging target patterns: a landscape figure and a dragon dance formation. These patterns include regions of varying density, disconnected substructures, and non-convex boundaries, which present significant challenges to any self-assembly algorithm.

The Mean-shift method demonstrated clear limitations when handling these shapes. Due to its reliance on local density gradients modulated by a fixed obstacle avoidance distance and sensing radius, it struggles to adapt to regions with drastically different spatial requirements. In narrow or sparse segments of the pattern, the density-based redistribution mechanism becomes ineffective, often resulting in large gaps or overcrowded clusters. Moreover, in cases where the target shape is composed of disjoint parts, the robots fail to reach certain isolated subregions, further degrading the overall shape fidelity. These issues are clearly reflected in Figs. 20–21. Additionally, when the initial robot distribution is offset from the shape center, as shown in Fig. 22, the Mean-shift method also fails to autonomously adjust the global positioning of the swarm.

In contrast, our proposed method successfully assembled both complex shapes with high coverage and uniformity. Unlike Mean-shift, our approach does not rely on fixed sensing or avoidance radii. Instead, it utilizes a global target allocation mechanism coupled with a uniform diffusion-inspired spreading strategy, enabling robots to adaptively navigate and fill areas of varying density and shape topology. This allows the swarm to naturally handle narrow passages, wide open spaces, and disconnected segments without explicit tuning of repulsion parameters. As a result, our method maintains formation integrity even in the most geometrically demanding scenarios.

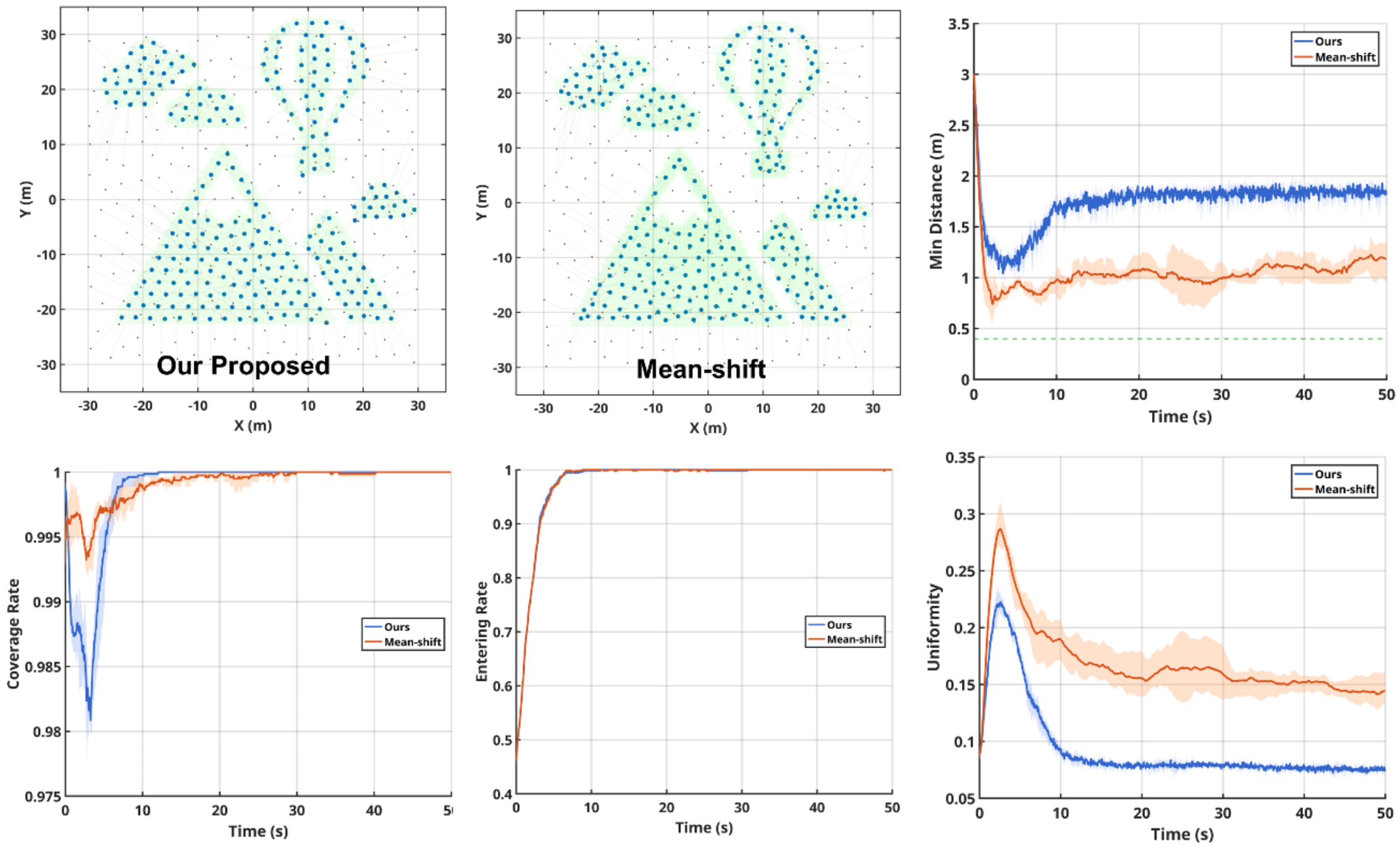

Fig. 20 | Comparison of shape assembly performance between our proposed method and the Mean-shift strategy on a complex landscape pattern. Results are averaged over 5 independent trials for each method. Shaded areas indicate the range between the maximum and minimum values observed across trials.

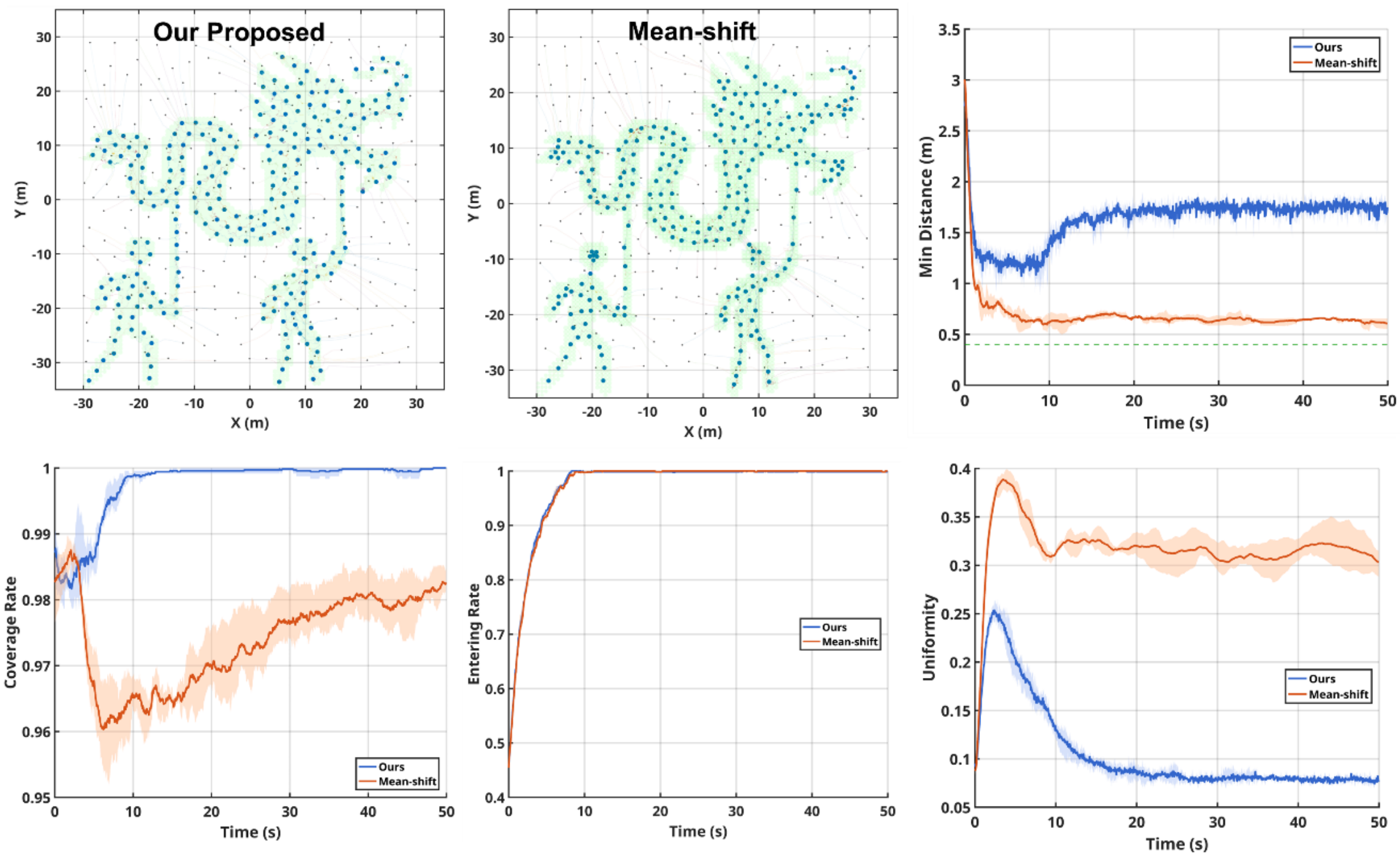

Fig 21 | Shape assembly performance on a complex dragon-dance pattern using our method and the Mean-shift strategy. Results are averaged over 5 independent trials for each method.

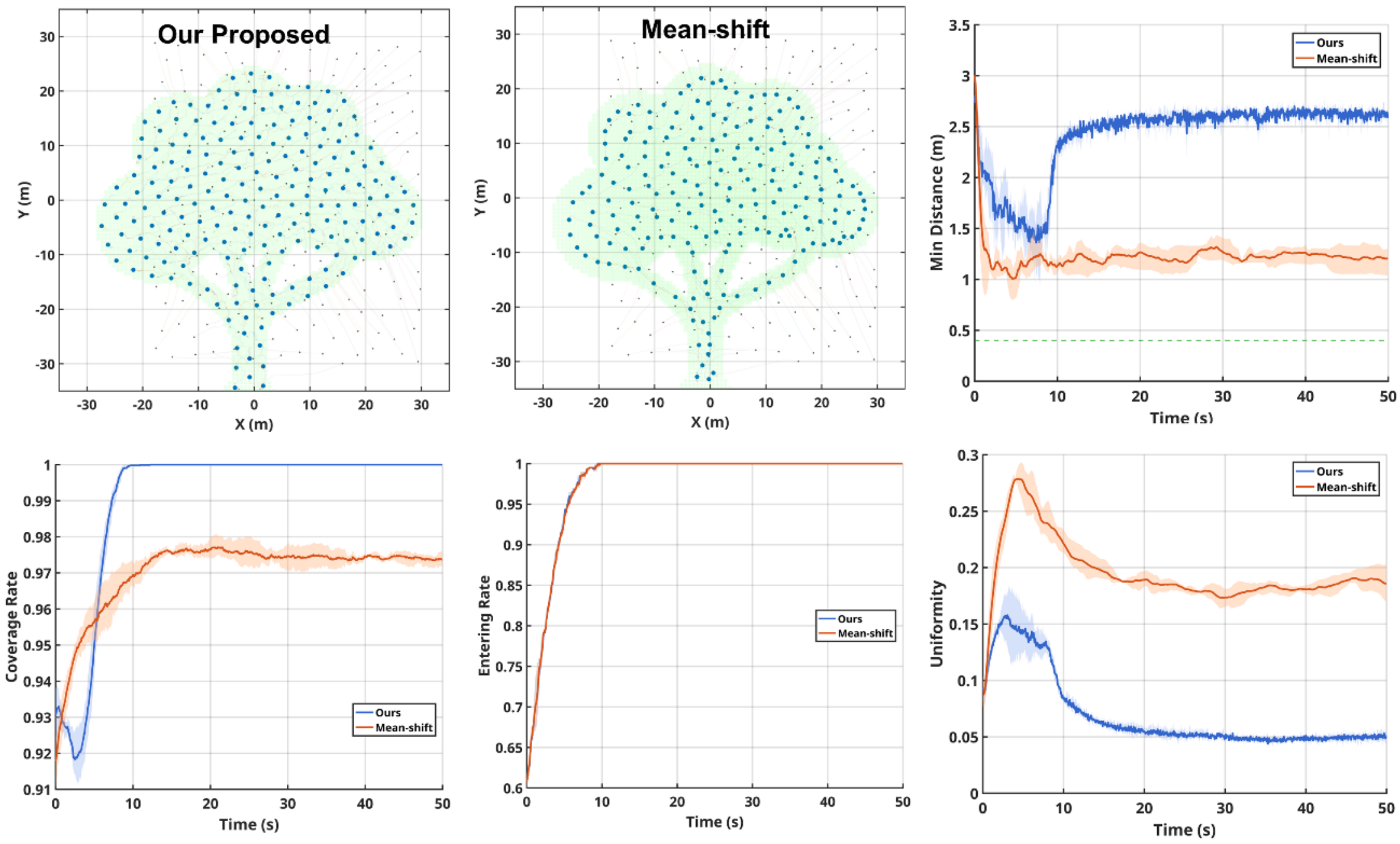

Fig. 22 | Shape assembly performance on a complex tree pattern using our method and the Mean-shift strategy. Robots are initially placed with a spatial offset from the shape center. Results are averaged over 5 independent trials for each method. Shaded areas indicate the range between the maximum and minimum values observed across trials.

5 Additional Experiments in Obstacles Environment

5.1 The model

5.1.1 In Airsim Simulator

To validate our algorithm in a more realistic setting, we implement swarm formation experiments using the Airsim simulator. Each UAV in the simulation is modeled with a size of 0.3 m × 0.3 m, which approximates the scale of small quadrotors used in practice. To perceive the surrounding environment, each UAV is equipped with a simulated LiDAR sensor. The LiDAR is configured with a RotationsPerSecond of 20 and PointsPerSecond of 10000, which provides basic obstacle awareness, as shown in Fig 21. However, it is important to note that this LiDAR setup is significantly less capable than commercial-grade LiDARs commonly used in real-world robotics, and thus represents a conservative sensing model. Each UAV localizes itself using simulated GPS data, and inter-robot communication is achieved through a custom communication interface that simulates message passing between robots.

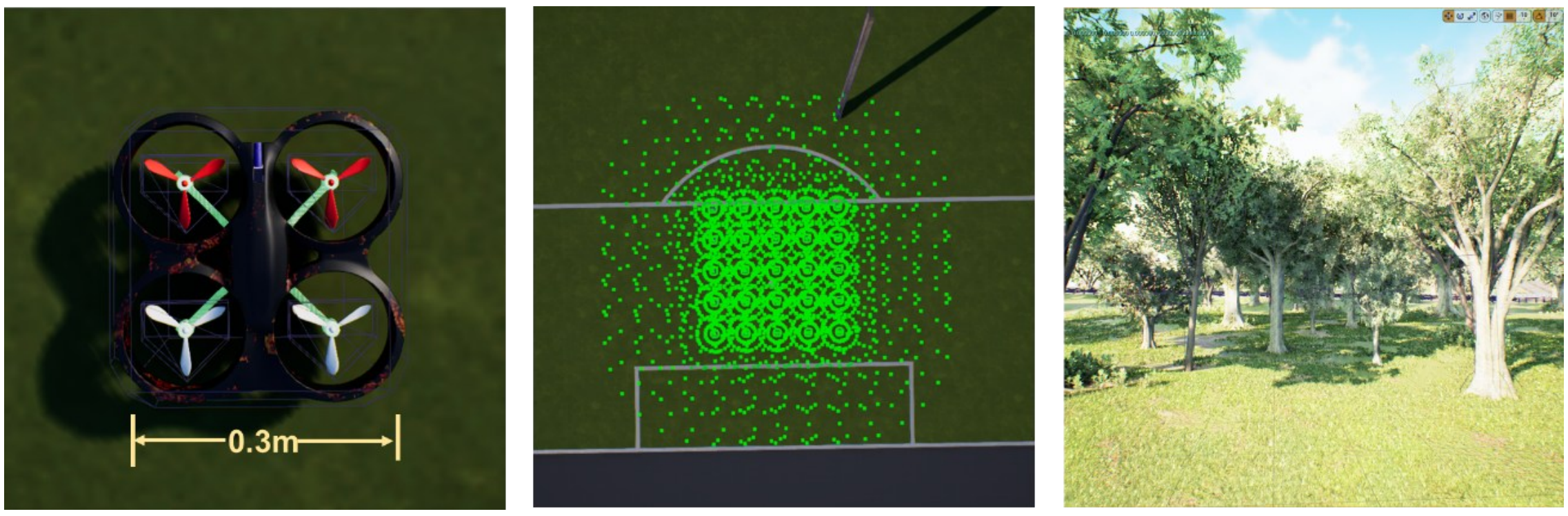

Fig. 23 | Simulation setup in the AirSim environment. (Left) A top-down view of a simulated drone with a size of 0.3 m × 0.3 m. Each drone is equipped with a simulated LiDAR sensor for obstacle perception. (Middle) Initial swarm configuration in an open environment. Green dots represent LiDAR sampling points generated by the drones. (Right) Realistic forest-like environment in AirSim used to simulate obstacle-rich scenarios.

5.1.2 In Real World

The real world experiment involves several key components: a Nokov Motion Capture system, multiple Crazyradio PA wireless communication modules, Crazyflie drones, and a workstation.

Nokov Motion Capture system: The experimental area measures 6 × 8 × 3 meters. The Nokov Motion Capture system is deployed to capture the precise positions of all drones within this space. It operates at a sampling frequency of 20 Hz, and the position data is transmitted in real-time to the workstation.

Crazyradio PA: Wireless communication is facilitated by six Crazyradio PA modules. Every three Crazyflie drones share the same frequency but are assigned unique physical addresses to avoid communication interference. Each Crazyradio PA handles communication with its assigned group of drones, delivering positioning information from the motion capture system as well as velocity control commands.

Crazyflie drones: The number of Crazyflie drones in the experiments ranges from 9 to 18. Each drone is equipped with four reflective markers to enable accurate localization, and LED indicators on each drone display its operational status, such as ready, locked, or low battery.

Workstation: All flight control commands are computed by a dedicated workstation equipped with an Intel i9-13900H CPU running at 2.6 GHz and 48 GB of RAM. Since the drones do not have onboard computers, control instructions are calculated on the workstation and then transmitted wirelessly to the drones in real time.

5.2 Swarm Navigation through Constrained Obstacles in Simulation

To further assess the performance and adaptability of our swarm formation algorithm, we simulate scenarios where the UAV swarm must navigate through constrained obstacle environments.

A representative case involves passing through narrow corridors, which challenge the swarm’s ability to maintain formation and avoid collisions under spatial limitations.

Fig 24 shows time-lapse snapshots of a UAV swarm navigating through a corridor in simulation. The snapshots illustrate the swarm's coordinated motion, obstacle avoidance, and formation maintenance at various timestamps. Fig 25 provides additional insights into the swarm's navigation performance. Fig 26 focuses on the mechanism of our swarm obstacle avoidance algorithm. In Figure 26c, the UAV with an orange shaded region does not have a corresponding local target position because its associate region is entirely inaccessible due to proximity to obstacles. Consequently, its shape assembly velocity is primarily controlled by the entering velocity, directing it toward the nearest reachable point. In contrast, the UAV with a green shaded region is located within the corridor and has a safe associate region, resulting in a local target position represented by a red cross. This demonstrates how our algorithm dynamically adjusts the UAVs' velocities and target positions based on their environmental context to ensure safe and efficient navigation through constrained obstacle environments.

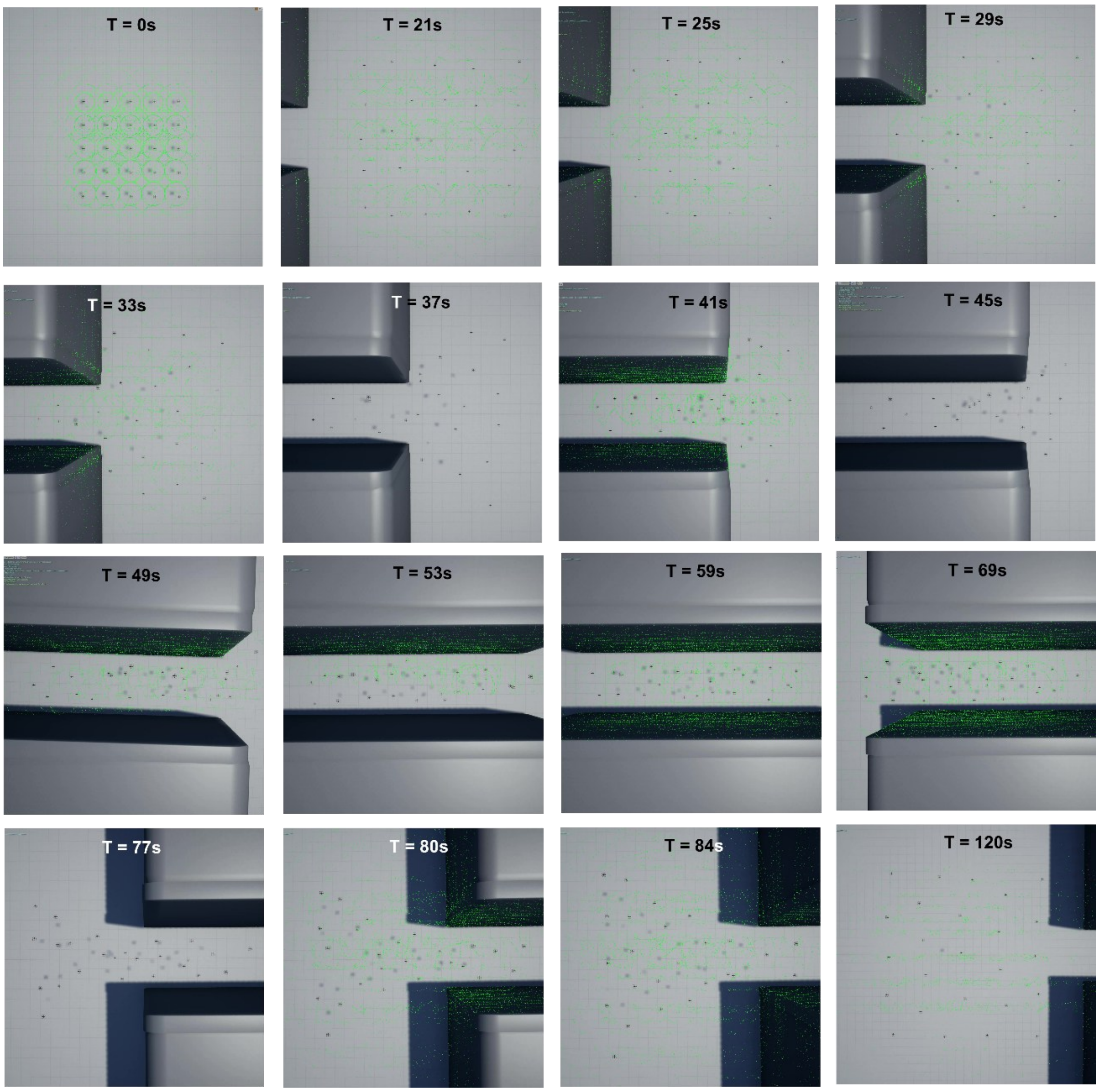

Fig. 24 | Time-lapse snapshots of a UAV swarm navigating through a narrow corridor in simulation at 2m/s. A group of 25 simulated quadrotors starts in a compact grid formation (T=0 s) and progressively flies through a corridor-like obstacle environment. The corridor width is approximately 4 m, requiring the swarm to adjust its configuration to avoid collisions. Each green dot represents a LiDAR sampling point perceived by the UAVs. The snapshots illustrate the swarm's coordinated motion, obstacle avoidance, and formation maintenance at various timestamps.

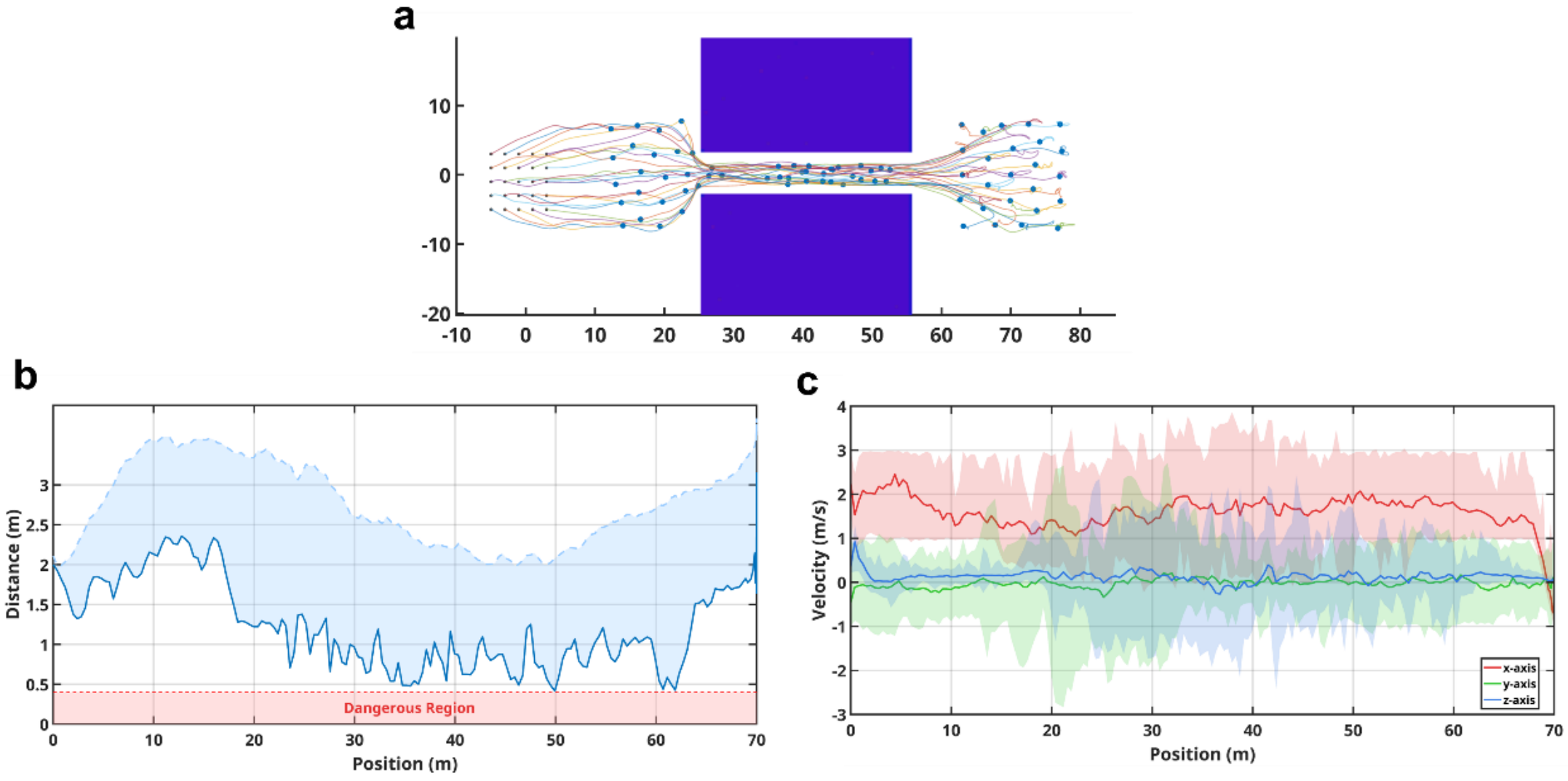

Fig 25 | Swarm Navigation Performance in Constrained Corridor Environment. a Trajectories of a UAV swarm navigating through a corridor obstacle environment. The purple square represents the corridor obstacle, and the colored lines with dots indicate the flight paths of UAVs. b Distance intervals between neighboring UAVs during swarm navigation. The shaded area represents the range of distances between neighboring UAVs. c Velocity profiles of UAVs during navigation. The colored lines represent the mean velocity along different axes and shaded areas indicate the maximum and minimum velocities.

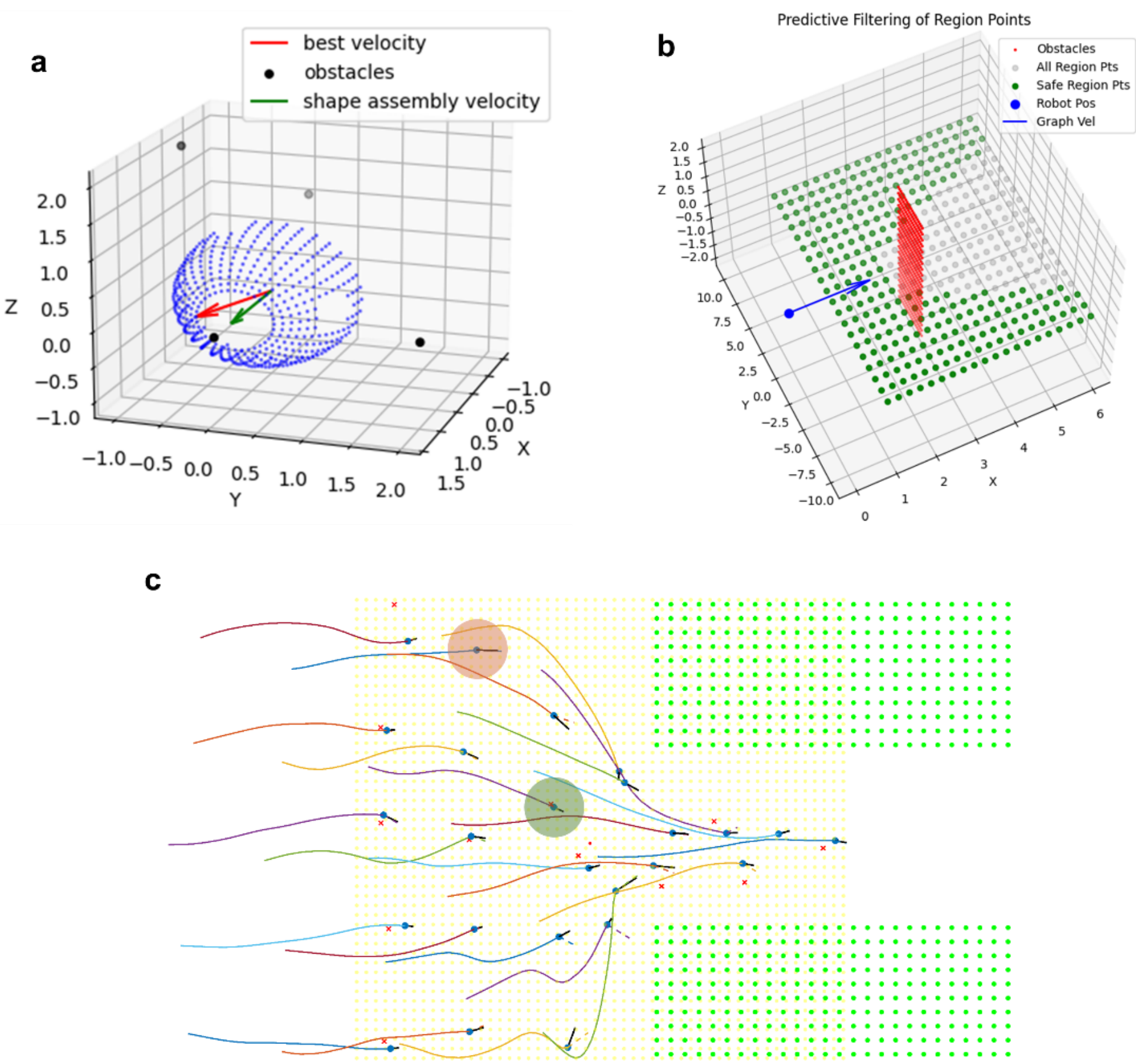

Fig 26 | Swarm Obstacle Avoidance Algorithm Mechanism. a Illustration of the shape assembly velocity deflection method. The black dots represent obstacles. A 60-degree cone surface centered on the UAV forms the candidate velocity set. Velocities pointing toward obstacles are excluded. The remaining candidate velocities are evaluated by calculating the weighted sum of angles with the previous velocity and the shape assembly velocity to select the optimal velocity (red arrow). The green arrow indicates the shape assembly velocity, and the blue dots represent candidate velocities. **b** Predictive filtering of region points. Green dots represent safe associate region points, red dots represent obstacle points, and gray dots are unreachable target points occluded by obstacles. Only green dots are used for target position calculation. The blue dot indicates the robot position, and the red sheet represents the obstacle-associated region. **c** Velocity distribution of UAVs navigating through a corridor. Blue dots represent UAVs, black lines indicate the optimal direction (v_{best}), colored dashed lines represent shape assembly velocities, and red crosses mark target positions computed using safe associate regions. The green grid represents safe navigation regions.

5.3 Swarm Navigation through Forest in Simulation

The trajectory visualization in Fig. 27 demonstrates that the proposed swarm navigation strategy enables all 49 drones to traverse a densely cluttered forest environment safely and efficiently. The swarm maintains smooth and coordinated motion despite the high obstacle density, which reflects the robustness of both local obstacle avoidance and inter-agent coordination mechanisms. Notably, the minimum obstacle distance remains consistently above the safety threshold throughout the trajectory, indicating that the collision avoidance algorithm performs reliably even under tight spatial constraints.

Fig. 28 presents a scenario where a UAV swarm faces significant challenges due to a densely packed forest environment. The forest, shown in the top-left corner, presents a nearly impassable terrain for the drones at their designated formation height. As seen in the lower-left corner, the trajectory of the swarm is visualized in a 3D plot, where the drones' paths are constrained by the dense obstacles in the horizontal plane. Due to the lack of feasible paths on the ground level, the swarm algorithm directs the drones to explore higher altitudes in search of a clear path. The path and trajectory variation indicate the exploration process as the drones adapt to the changing terrain, confirming that the swarm can maintain safe and coordinated movement even in complex, obstacle-rich environments.

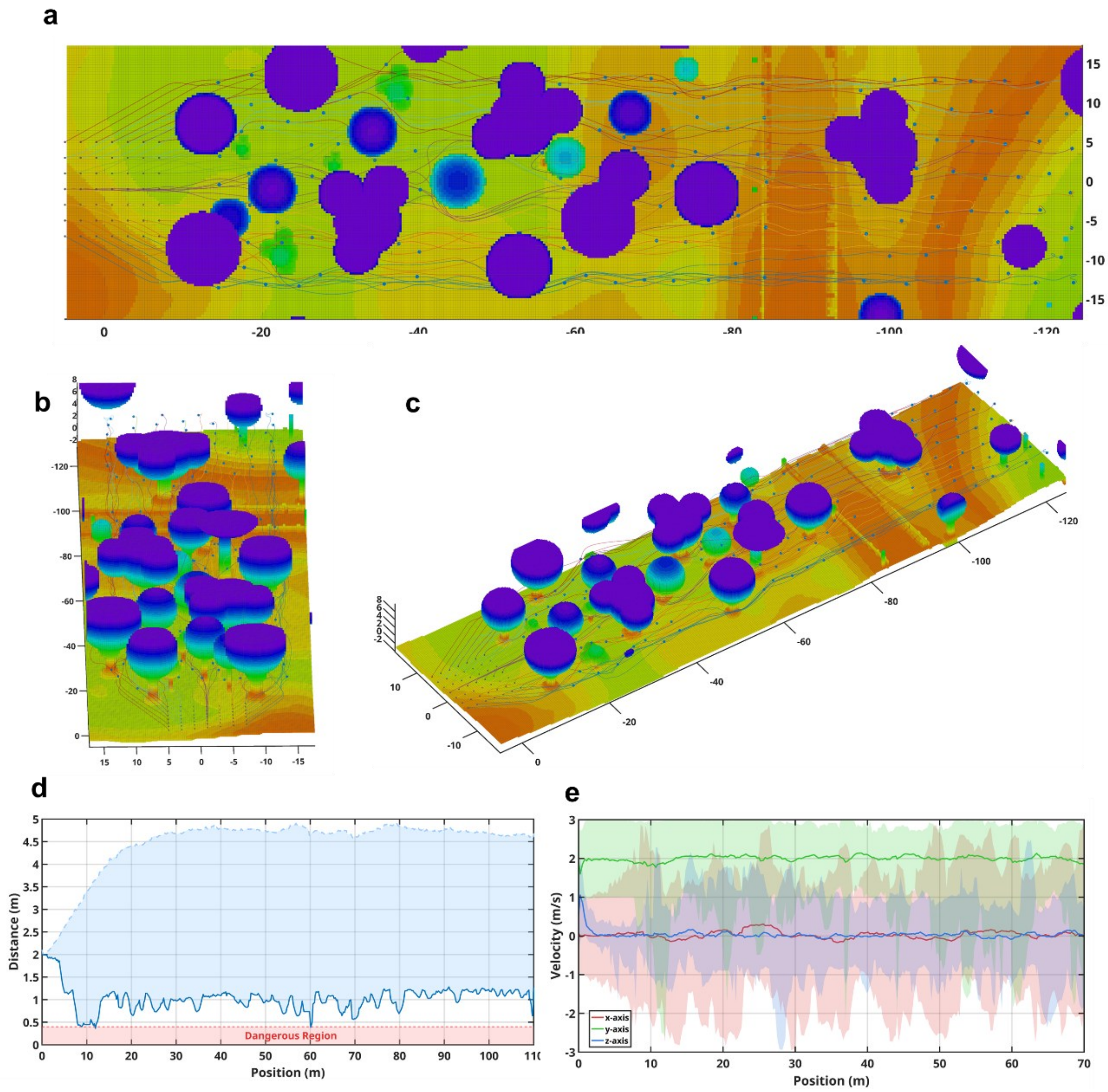

Fig 27 | 49 drones flying through forest at 2m/s in Airsim simulator. a Top view of the 3D trajectory of 49 drones navigating through a densely cluttered forest area. **b** Side view. **c** 3D perspective of the swarm trajectory. **d** Minimum distance to obstacles as a function of traveled distance. The swarm maintains a safe clearance above the danger threshold (red dashed line), the blue shaded area represents the distribution of inter-robot distances **e** Velocity profile of the swarm along x, y, and z axes.

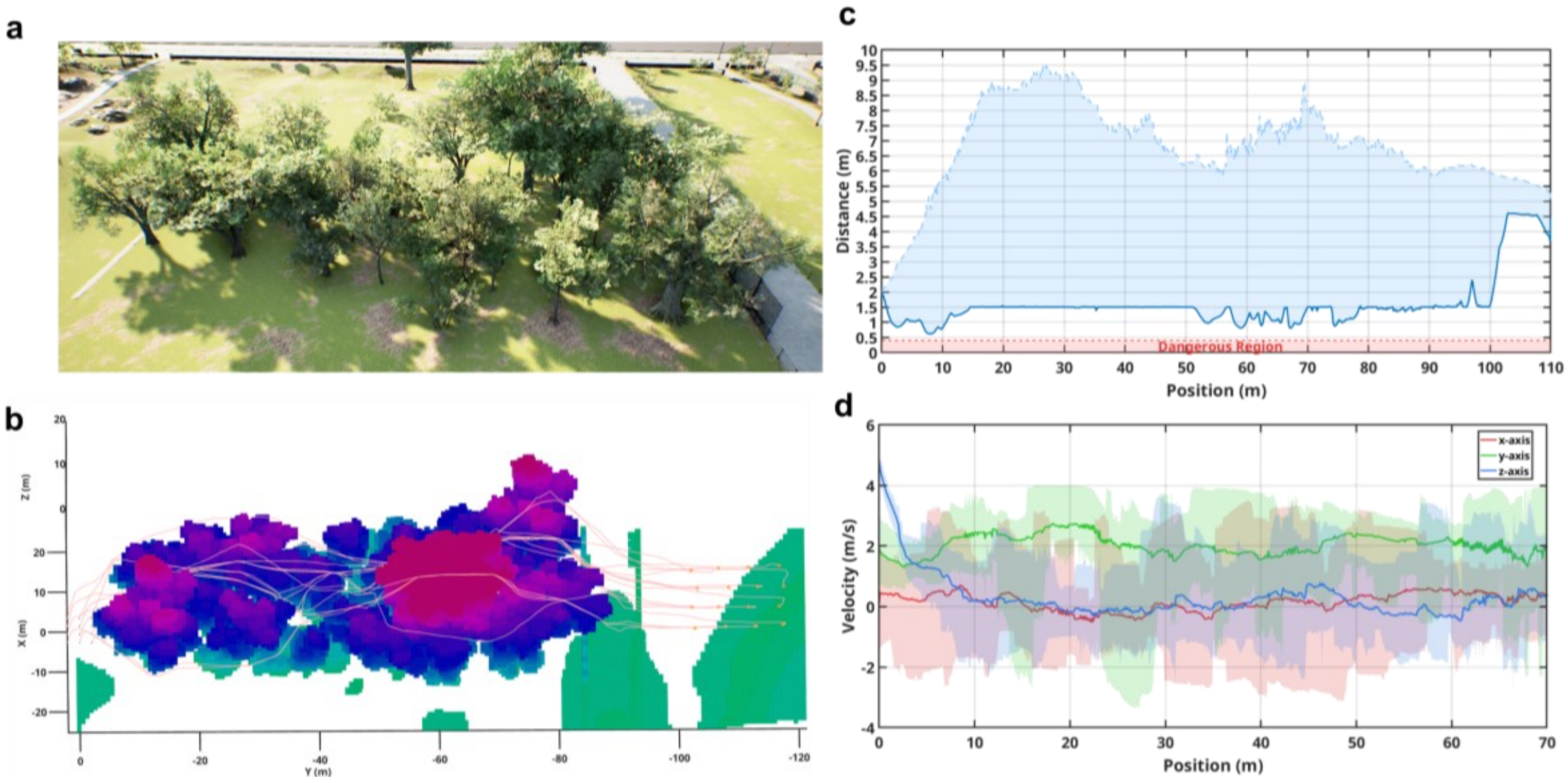

Fig 28 | 16 drones flying through forest at 2m/s in Airsim simulator.

6 Distributed Deployment of Large Language Models in Robot Swarm

6.1 The Performance and Experimental Results of the Distributed Model

Our method groups the robots and assigns a subset of the large-language model's parameters to each group. Robots in the same group share the same parameters and thus have identical inputs and outputs, so in our hardware-in-the-loop experiments as shown in Fig c each group of robots is represented by a single Raspberry Pi 4B acting as the onboard computer for that group.

To evaluate distributed deployment of large-language model on a robot swarm, we ran StableLM-3B-4E1T, Llama2-7B and Llama2-13B over five Raspberry Pi 4B and measured the latency per generated token. As shown in Fig. 29a, increasing the number of groups from one to four gradually reduces the latency for all models. When a fifth device is added, the latency of Llama2-7B shows a slight increase, indicating that further speedup is limited by communication overhead. Fig b compares our collaborative scheme with Llama.cpp Message Passing Interface (MPI) on 4 Raspberry Pi for Llama2-7B in 8-bit and 16-bit formats and Llama2-13B in 8-bit format. In Llama.cpp MPI the model is split by layers and each token state is passed from one device to the next, each device runs its own block of layers in sequence. In a robot swarm connected over Wi-Fi, this design makes robots spend most of their time waiting for the previous layer's output instead of performing local matrix multiplications. Our method instead splits the weight tensors inside each layer across robots, so that they can perform the matrix multiplications in parallel. As a result, in all three model settings our per-token latency is around half of that of Llama.cpp MPI.

To evaluate how different model sizes support task-level autonomy, we designed a five-stage search-

and-rescue mission and let Llama2-7B and Llama2-13B control the same swarm through our high-level shape-assembly interface in the AirSim simulator. The swarm consists of 16 robots represented

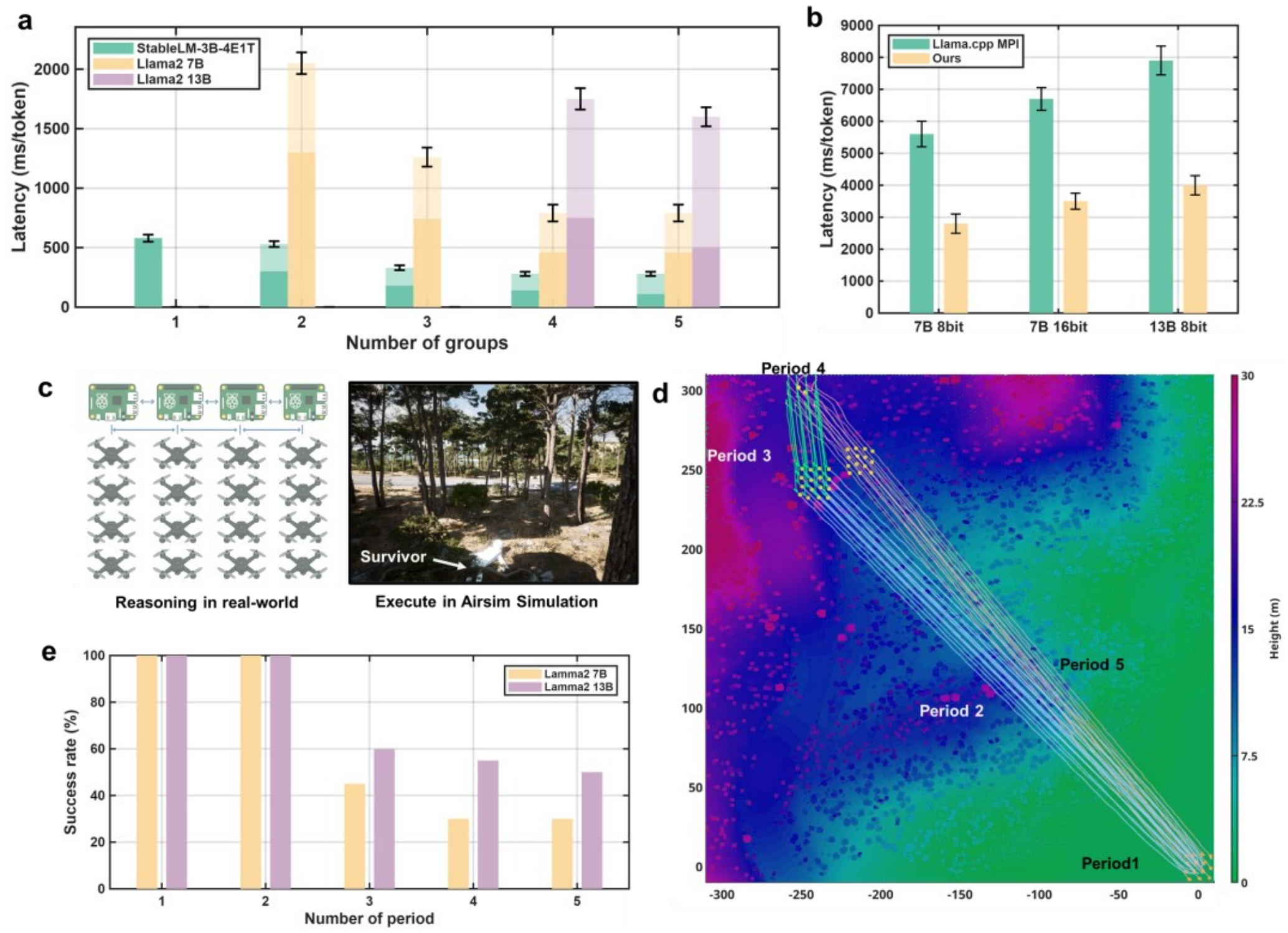

Fig 29 | Distributed deployment of large-language models and task performance. **a** Per-token latency of three LLMs. **b** Latency comparison between our method and Llama.cpp MPI**.** **c** Experiment setup in hardware-in-the-loop simulation environment. **d** Example five-stage search-and-rescue mission. **e** Success rate of each mission period over 20 trials for Llama2-7B and Llama2-13B.

by 4 Raspberry Pi and the swarm executes the mission in five periods: 1) forming a prescribed search formation, 2) flying to a target region, 3) searching the region to find the survivor, 4) detaching two robots to stay and deliver supplies, and 5) guiding the remaining robots back to the starting point. The LLM issues only high-level commands, while the underlying shape-assembly controller executes these commands on individual robots. Fig d shows a typical successful run with five distinct phases of motion. Fig e reports the success rate of each stage over twenty trials. Both models complete the first two stages almost perfectly, since they mainly require interpreting simple instructions and invoking fixed primitives. The success rate drops sharply at the third “search” stage, especially for the 7B model, because this stage requires the model to autonomously propose new waypoints, remember previously visited areas and adjust the search strategy over time. The 13B model handles this long-horizon reasoning more reliably and also performs better in the later “detach” and “return” stages. These results indicate that our distributed inference framework can execute complex multi-stage missions, and that larger models provide clear benefits when long-term memory and planning are required.

6.2 Method of Partitioning Large Language Models in Robot Swarms

We adopt a tensor-parallel partitioning scheme that splits the weight matrices of the large language model across the swarm. Concretely, for each linear weight matrix $W \in R^{d_{in} \times d_{out}}$ in the LLM, we partition it across P tensor-parallel groups using a standard tensor-parallel scheme (column-parallel or row-parallel depending on the layer), robots in group p store the same $W^{(p)}$, as illustrated in Fig7d. For column-parallel layers, we partition the matrix into P column blocks $W^{(p)} \in R^{d_{in} \times d_{out}/P}$.

Given the embedded prompt $X \in \mathbb{R}^{N \times d_{\text{in}}}$, inside each Transformer block, the robots perform two rounds of intermediate-result communication: one to assemble the attention outputs $O^{(p)}$and one to assemble the MLP outputs $M^{(p)}$. As shown in Fig7c, for the attention process, the input first passes through the Root Mean Square Layer Normalization (RMSNorm), for simplicity we still denote the normalized feature by X. Robots in group p compute their local queries, keys and values as:

$$Q^{(p)} = XW_Q^{(p)}\ , \quad K^{(p)} = XW_K^{(p)}\ , \quad V^{(p)} = XW_V^{(p)}. \tag{19}$$

Rotary position embeddings (RoPE) are applied to $Q^{(p)}$and $K^{(p)}$to obtain $\tilde{Q}^{(p)}$and $\tilde{K}^{(p)}$. Each group then evaluates its attention output:

$$A^{(p)} = \text{softmax}\left(\frac{\tilde{Q}^{(p)}\tilde{K}^{(p)T}}{\sqrt{d_h}}\right), \quad O^{(p)} = A^{(p)}V^{(p)}. \tag{20}$$

All robots broadcast their local $O^{(p)}$ over the wireless network, and the swarm concatenates these blocks along the feature dimension to reconstruct the full attention output $O = [\ O^{(1)}, \dots, O^{(P)}\ ]$. After this communication step, every robot holds the same full O.

The MLP process operates on the full attention output O. It uses three weight matrices $W_{\text{up}}, W_{\text{gate}}, W_{\text{down}}$, where W_{up} and W_{gate} are partitioned column-wise so that group p stores $W_{\text{up}}^{(p)}$ and $W_{\text{gate}}^{(p)}$, while W_{down} is partitioned row-wise so that group p stores $W_{\text{down}}^{(p)}$. Robots in group p compute:

$$u^{(p)} = OW_{\text{up}}^{(p)}, g^{(p)} = OW_{\text{gate}}^{(p)},$$

$$h^{(p)} = \text{SiLU}\big(g^{(p)}\big) \odot u^{(p)}, M^{(p)} = h^{(p)}W_{\text{down}}^{(p)}, \tag{21}$$

where SiLU denotes the sigmoid linear unit and $M^{(p)}$ is the shard-wise MLP output. As in standard

tensor parallelism**[15]**, all robots perform a sum-reduction (broadcast + summation) over their local $M^{(p)}$ to obtain the full MLP output $M = \sum_{p=1}^{P} M^{(p)}$, which is then used as the input X of the next Transformer block.

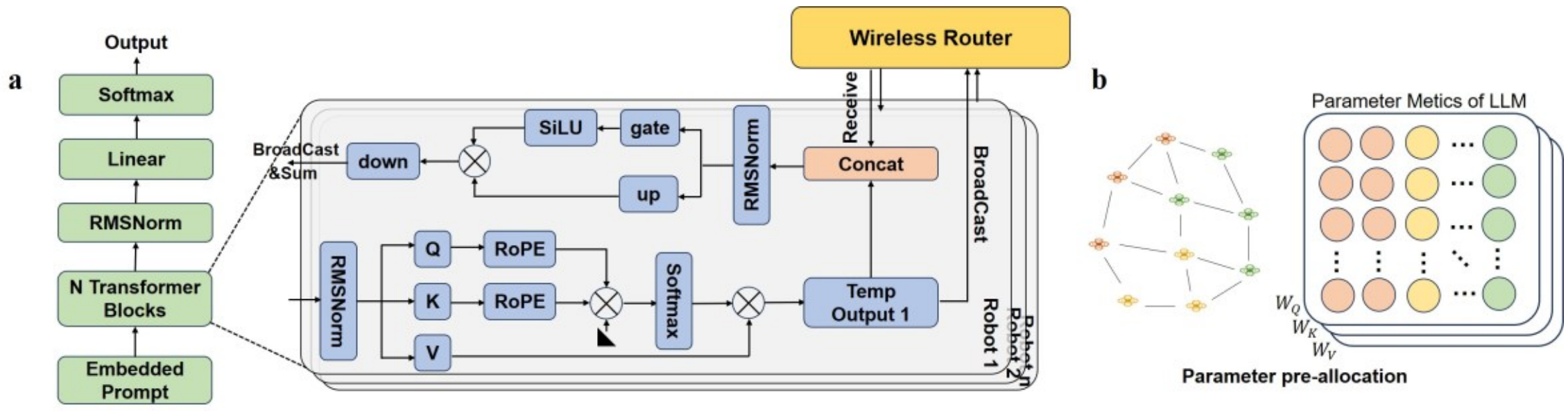

Fig. 30 | Distributed tensor-parallel LLM inference in a robot swarm. (a) Each Transformer block is executed collaboratively: attention shards are exchanged and concatenated, while the MLP "down" output is aggregated via broadcast-and-sum over Wi-Fi. (b) Model parameters are pre-allocated as tensor shards to robot groups for parallel matrix multiplications during inference.

With this tensor-parallel execution inside each Transformer block, the swarm can run end-to-end autoregressive inference in a distributed manner, enabling high-level closed-loop control as follows. The user first provides the mission description and a graph-based target shape, together with the initial swarm state. The LLM then performs collaborative inference across the swarm, maintaining and updating its internal planning state based on sensor-derived feedback, including textual summaries and extracted features. At each iteration, it selects a sequence of high-level actions from the available tool modules, such as formation generation, target commands, and swarm management. These actions are executed on the robots in the real world, producing updated positions and LiDAR observations, which are fed back to the distributed LLM for state checking and replanning. This perception–decision–action loop enables the swarm to autonomously form shapes, navigate unknown obstacle environments, and adapt its objectives online.

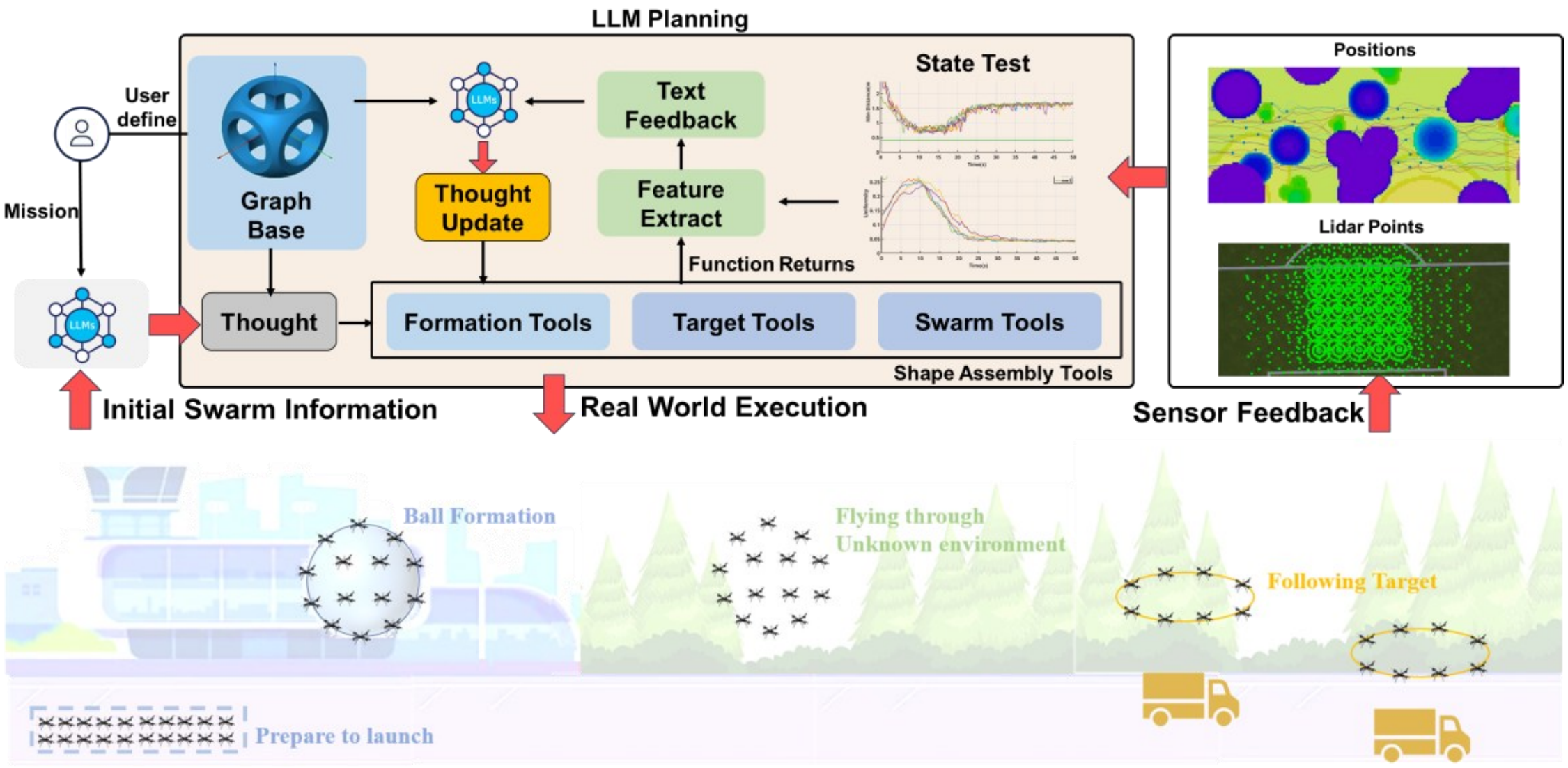

Fig. 31 | Workflow of the LLM-integrated swarm system. Control process of a large language model with event-trigger feedback.

Prompt

You are an AI planner for a drone swarm in a mountain search-and-rescue mission. You must output a sequence of function calls to complete the mission:

- formation(<shape>) where <shape> is either sphere or square
- sperate() splits the current swarm into two swarms to bring supply to survivor
- publish_destination([x,y,z], [x,y,z], ...) publishes one or more destination waypoints for the active swarm to follow in order.

Each round, you will receive the current system status:

- Formation status: "pending" / "complete“
- Coordinates of the trapped person: (x, y, z) if found, else None
- Current swarm location: (x, y, z) indicating the swarm's current position
- Swarm target location: (x, y, z) indicating the target destination
- destination_arrival: True/False (whether the swarm has arrived at the target destination)
- Task_history: The actions that have already been carried out.

Swarm configuration:

- Initial swarm center coordinates: [0, 0, 0]

Mission:

A person is trapped within a square area centered at (-250, 250), with a 100-meter range. Your task is to find the trapped person, leave two drone to communicate with them, and have the remaining drones return to base while mapping the path at a low altitude (6m) to create a rescue route for the rescuers.

Supplementary References

[1] Sun G, Zhou R, Ma Z, et al. Mean-shift exploration in shape assembly of robot swarms[J]. Nature Communications, 2023, 14(1): 3476.

[2] Li X, Zhou R, Sun G. Adaptive shape formation against swarm-scale variants in robot swarms[J]. IEEE Robotics and Automation Letters, 2024, 9(8): 6776-6783.

[3] Zhang Y, Zhou R, Li X, et al. Mean-shift shape formation of multi-robot systems without target assignment[J]. IEEE Robotics and Automation Letters, 2024, 9(2): 1772-1779.

[4] Vásárhelyi, G., Virágh, C., Somorjai, G., Nepusz, T., Eiben, A. E., & Vicsek, T. Optimized flocking of autonomous drones in confined environments. Science Robotics, 3, eaat3536 (2018)

[5] Yu C, Zhang D, Zhang Q. GRF-based predictive flocking control with dynamic pattern formation[C]//2024 IEEE International Conference on Robotics and Automation (ICRA). IEEE, 2024: 16788-16794.
[6] Cheng J, Cheng W, Nagpal R. Robust and self-repairing formation control for swarms of mobile agents[C]//AAAI. 2005, 5(2005).
[7] Zhang Z, Piao S. Vein-based coalitions for multi-agent pattern formation tasks[J]. IEEE Robotics and Automation Letters, 2022, 7(4): 12379-12386.
[8] Wang H, Rubenstein M. Shape formation in homogeneous swarms using local task swapping[J]. IEEE Transactions on Robotics, 2020, 36(3): 597-612.
[9] Chu W, Zhang W, Zhao H, et al. Massive shape formation in grid environments[J]. IEEE Transactions on Automation Science and Engineering, 2022, 20(3): 1745-1759.
[10] Liu, C. L., Ridgley, I. L. D., Elwin, M. L., Rubenstein, M., Freeman, R. A., & Lynch, K. M. Self-Healing Distributed Swarm Formation Control Using Image Moments. IEEE Robotics and Automation Letters, 9, 6216-6223, (2024).
[11] Soria, E., Schiano, F., & Floreano, D. Predictive control of aerial swarms in cluttered environments. Nature Machine Intelligence, 3, 545-554 (2021)
[12] Slavkov I, Carrillo-Zapata D, Carranza N, et al. Morphogenesis in robot swarms[J]. Science Robotics, 2018, 3(25): eaau9178.
[13] Qu Y, Xin B, Wang Q, et al. Automated Discovery of Efficient Behavior Strategies for Distributed Shape Formation of Swarm Robots by Genetic Programming[J]. IEEE Transactions on Automation Science and Engineering, 2023, 21(4): 7599-7615.
[14] Breitenmoser A, Schwager M, Metzger J C, et al. Voronoi coverage of non-convex environments with a group of networked robots[C]//2010 IEEE international conference on robotics and automation. IEEE, 2010: 4982-4989.
[15] Cao K, Chen Y, Gao S, et al. Multi-Robot Formation Control Based on CVT Algorithm and Health Optimization Management[J]. Applied sciences, 2022, 12(2): 755.
[16] Zhou X, Wen X, Wang Z, et al. Swarm of micro flying robots in the wild[J]. Science Robotics, 2022, 7(66): eabm5954.
[17] Agarwal S, Akella S. Simultaneous optimization of assignments and goal formations for multiple robots[C]//2018 IEEE international conference on robotics and automation (ICRA). IEEE, 2018: 6708-6715.
[18] Bragg (Sir), L., & Nye, J. F. A dynamical model of a crystal structure. Proceedings of the Royal Society A: Mathematical and Physical Sciences, 190, 474-481(1947).
[19] Liu H, Cao G. Effectiveness of the Young-Laplace equation at nanoscale[J]. Scientific reports, 2016, 6(1): 23936.
[20] Khestanova, E., Guinea, F., Fumagalli, L. et al. Universal shape and pressure inside bubbles appearing in van der Waals heterostructures. Nat Commun 7, 12587 (2016).

[21] Verrelli, David I., Peter TL Koh, and Anh V. Nguyen. "Particle–bubble interaction and attachment in flotation." *Chemical Engineering Science* 66.23 (2011): 5910-5921.

[22] Cox, S., Morgan, F., & Graner, F. (2012). Are large perimeter- minimizing two-dimensional clusters of equal-area bubbles hexagonal or circular?. Proceedings of the Royal Society A: Mathematical, Physical and Engineering Sciences, 469. https://doi.org/10.1098/rspa.2012.0392.

[23] Zhou HX. Protein folding in confined and crowded environments. Arch Biochem Biophys. 2008 Jan 1;469(1):76-82. doi: 10.1016/j.abb.2007.07.013. Epub 2007 Aug 1. PMID: 17719556; PMCID: PMC2223181.

[24] Polyak, B. , & Shcherbakov, P. . (2017). Lyapunov functions: an optimization theory perspective. Ifac Papersonline, 50(1), 7456-7461.

[25] Q. Du et al. Centroidal Voronoi Tessellations: Applications and Algorithms, 1999.

[26] Peng Chen, Yongmei Chen, and Beibei Jin. 2018. A new soft assignment K-means algorithm. In Proceedings of the 2nd International Conference on Machine Learning and Soft Computing (ICMLSC '18). Association for Computing Machinery, New York, NY, USA, 57–61. https://doi.org/10.1145/3184066.3184073

[27] Chiang, A., & Liao, S. (2017). Image analysis with symmetry properties of Legendre moments. 2017 2nd International Conference on Image, Vision and Computing (ICIVC), 386-390. https://doi.org/10.1109/ICIVC.2017.7984583.

[28] Riesen, K., & Bunke, H. (2009). Approximate graph edit distance computation by means of bipartite graph matching. Image Vis. Comput., 27, 950-959. https://doi.org/10.1016/j.imavis.2008.04.004.

[29] Shoeybi, M., Patwary, M., Puri, R., LeGresley, P., Casper, J., & Catanzaro, B. Megatron-LM: Training Multi-Billion Parameter Language Models Using Model Parallelism. arXiv:1909. 08053 (2020).

[30] Ainslie, J., Lee-Thorp, J., de Jong, M., Zemlyanskiy, Y., Lebrón, F., & Sanghai, S. GQA: Training Generalized Multi-Query Transformer Models from Multi-Head Checkpoints. arXiv:2305.13245 (2023).

[31] Shazeer, N. Fast Transformer Decoding: One Write-Head is All You Need. arXiv:1911.02150 (2019).
