# Supplementary material for: Bubble-raft inspired shape-assembly in flying robot swarm for uniform formation and obstacle traversal
Source: Commun Eng. 2026 Jul 2;5:178. doi: 10.1038/s44172-026-00715-3 (PMC13696712; doi:10.1038/s44172-026-00715-3)
Supplement: Supplementary file 3 [file 44172_2026_715_MOESM3_ESM.pdf]

Description of Additional Supplementary Files:

File name: Supplementary Video 1

Description: Complex shape-assembly for 3D shapes—Transformation from spherical to DNA-shaped formation

File name: Supplementary Video 2

Description: Formation transition between letters “SWARM”

File name: Supplementary Video 3

Description: Shape-recovery in dynamic swarm size

File name: Supplementary Video 4

Description: Comparison with three state-of-the-art methods

File name: Supplementary Video 5

Description: Flying through unknown dense environment using lidar in simulation

File name: Supplementary Video 6

Description: Obstacles Avoidance in dense environment in real world

File name: Supplementary Video 7

Description: Autonomous search and rescue via LLM-Guided swarm shape assembly
